# Supplementary material for: Transancestral mapping and genetic load in systemic lupus erythematosus
Source: Nat Commun. 2017 Jul 17;8:16021. doi: 10.1038/ncomms16021 (PMC5520018; doi:10.1038/ncomms16021)

SI GUIDE

Title of file for HTML: Supplementary Information
Description: Supplementary Figures.

Supplementary Data

Title of file for HTML: Supplementary Data 1
Description: Sample counts by SLE status

Title of file for HTML: Supplementary Data 2
Description: Complete list of associations by ancestry and tier (nonHLA).

Title of file for HTML: Supplementary Data 3
Description: Posterior probabilities for HLA imputation

Title of file for HTML: Supplementary Data 4
Description: Classical HLA associations.

Title of file for HTML: Supplementary Data 5
Description: HLA stepwise model results

Title of file for HTML: Supplementary Data 6
Description: SNP associations, post-adjustment for HLA alleles.

Title of file for HTML: Supplementary Data 7
Description: SLE risk for DRB1 homozygotes and heterozygotes

Title of file for HTML: Supplementary Data 8
Description: Secondary top meta-analysis and transancestral-mapping Regions.

Title of file for HTML: Supplementary Data 9
Description: Meta-analysis regions with p-valueFDR <0.001.

Title of file for HTML: Supplementary Data 10
Description: Deviations in average risk-allele admixture.

Title of file for HTML: Supplementary Data 11
Description: Binomial test of CEU vs YRI admixture deviations.

Title of file for HTML: Supplementary Data 12
Description: cisEQTLs for regions in Tier 1 and Tier 2

Title of file for HTML: Supplementary Data 13
Description: Most likely functional SNPs based on RegulomeDB scores

Title of file for HTML: Supplementary Data 14
Description: Prioritized SLE risk genes.

Title of file for HTML: Peer Review File
Description:

Supplementary Figure 1. Admixture by ancestry.

a. European Ancestry

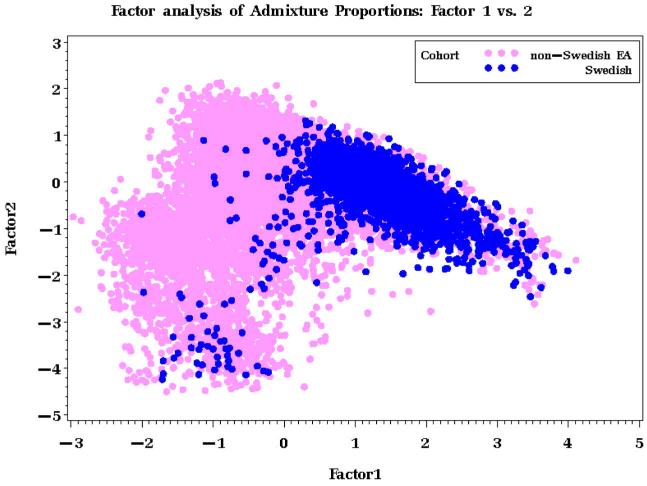

b. African American

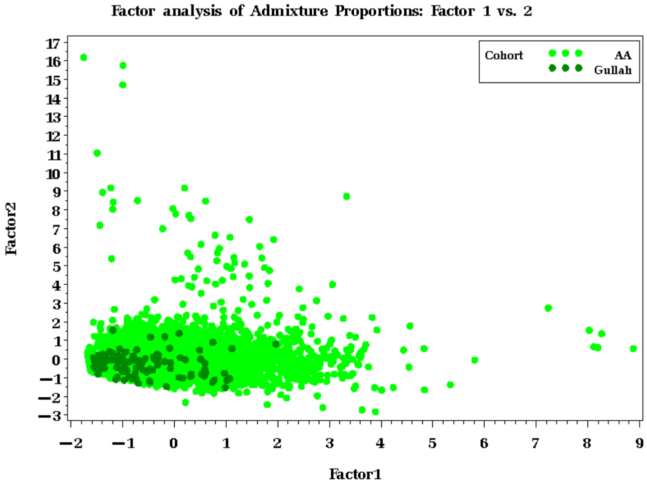

c. Hispanic Ancestry

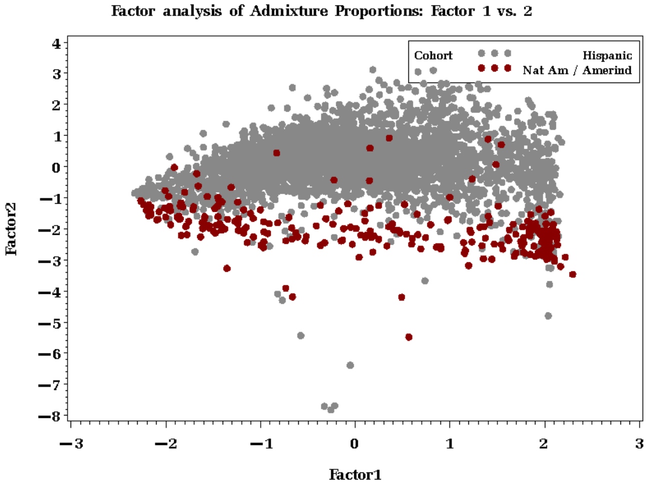

Supplementary Figure 2. PP-plot by ancestry.

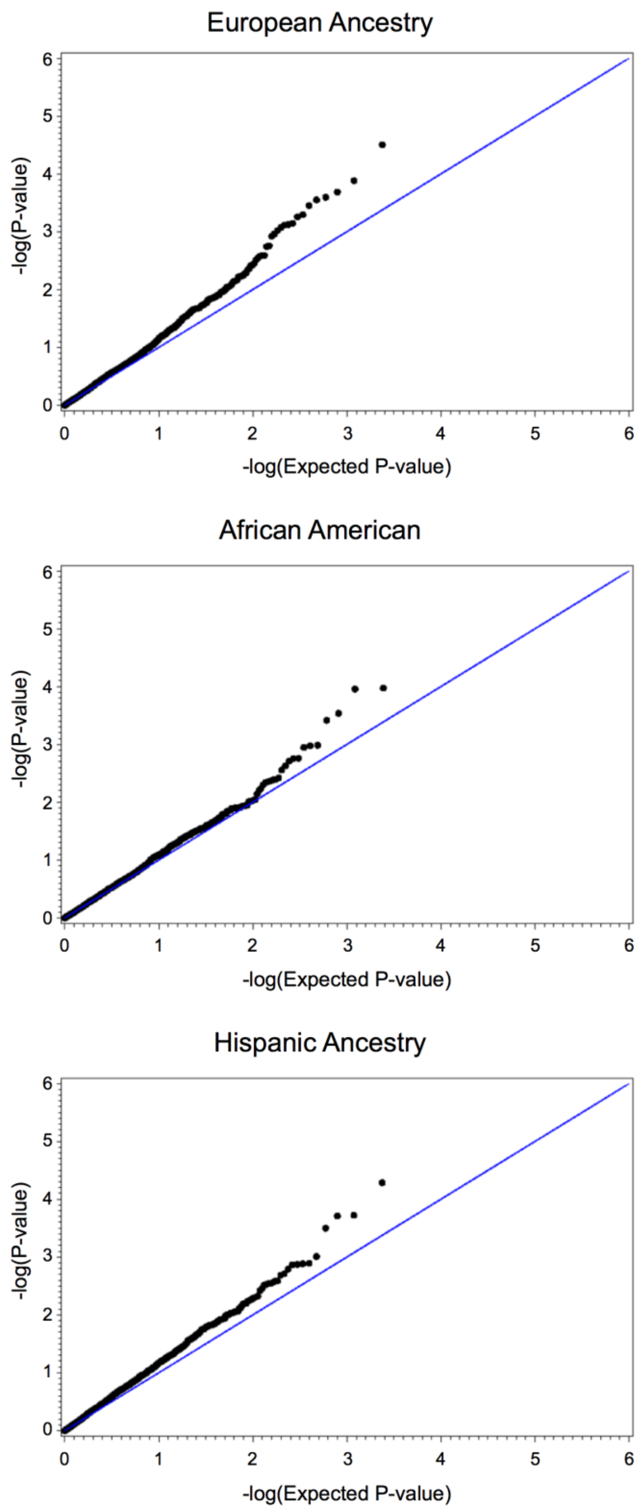

Supplementary Figure 3. Power by ancestry (α=5x10^{-8}).

a. European Ancestry

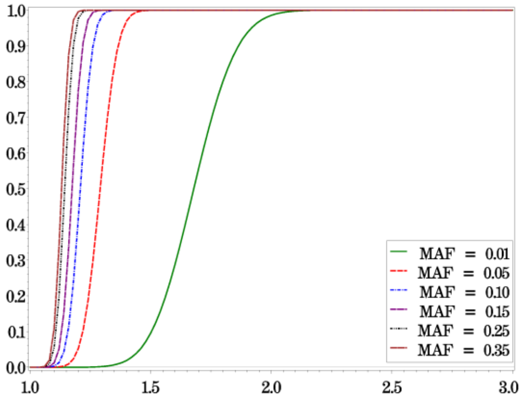

b. African American

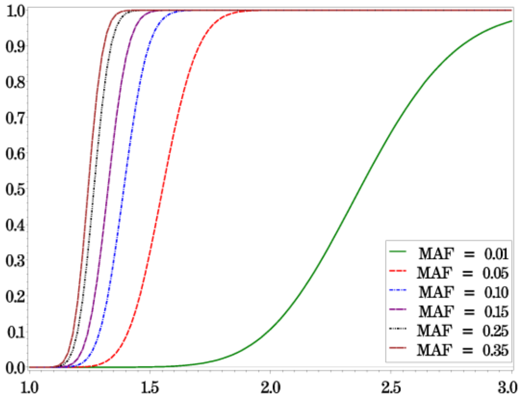

c. Hispanic Ancestry

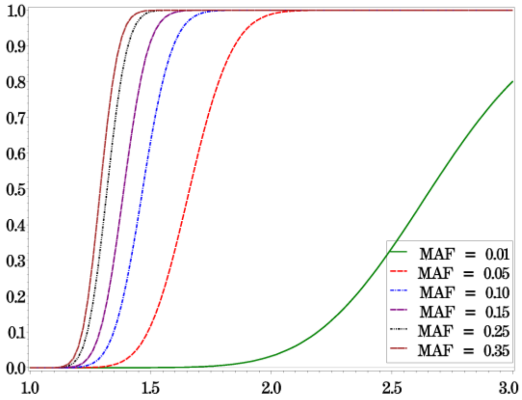

d. Meta-analysis .

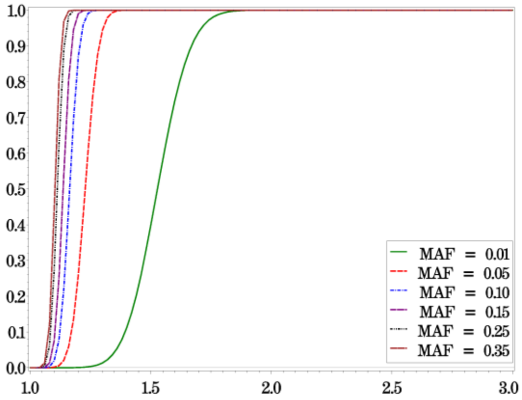

Supplementary Figure 4. Meta-analysis of chromosome X region of association (female only).

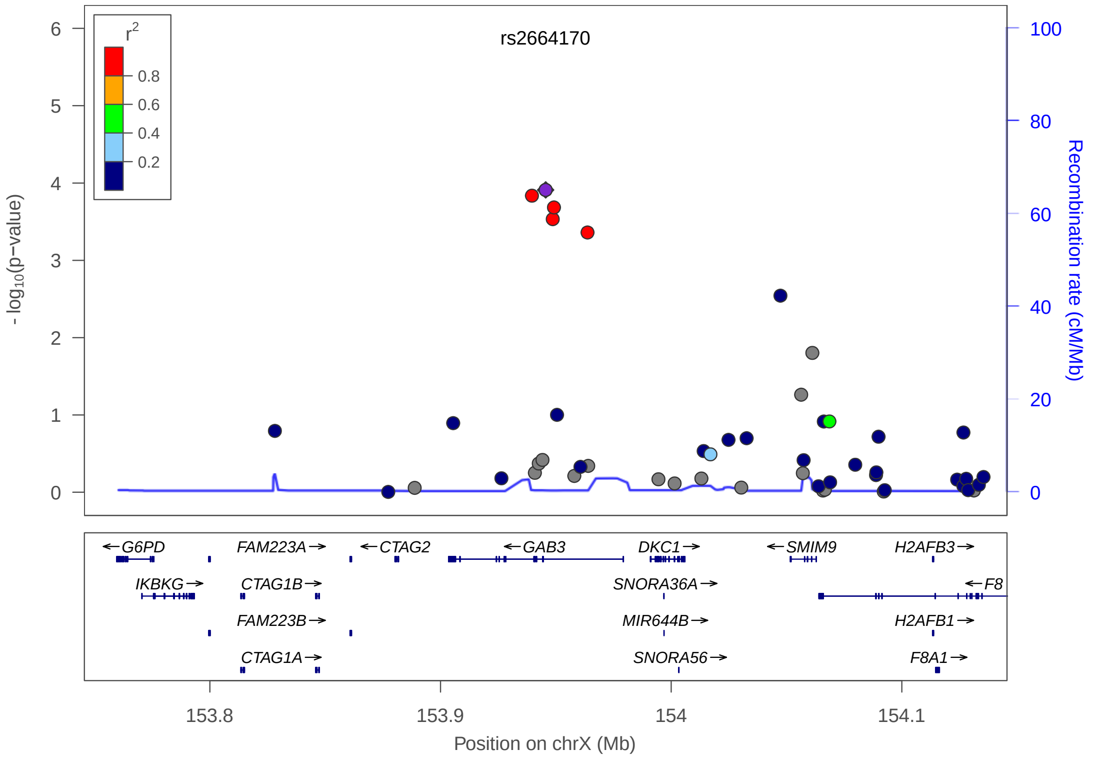

Supplementary Figure 5. Posterior probability distributions for best guess HLA imputed alleles.

a. European Ancestry

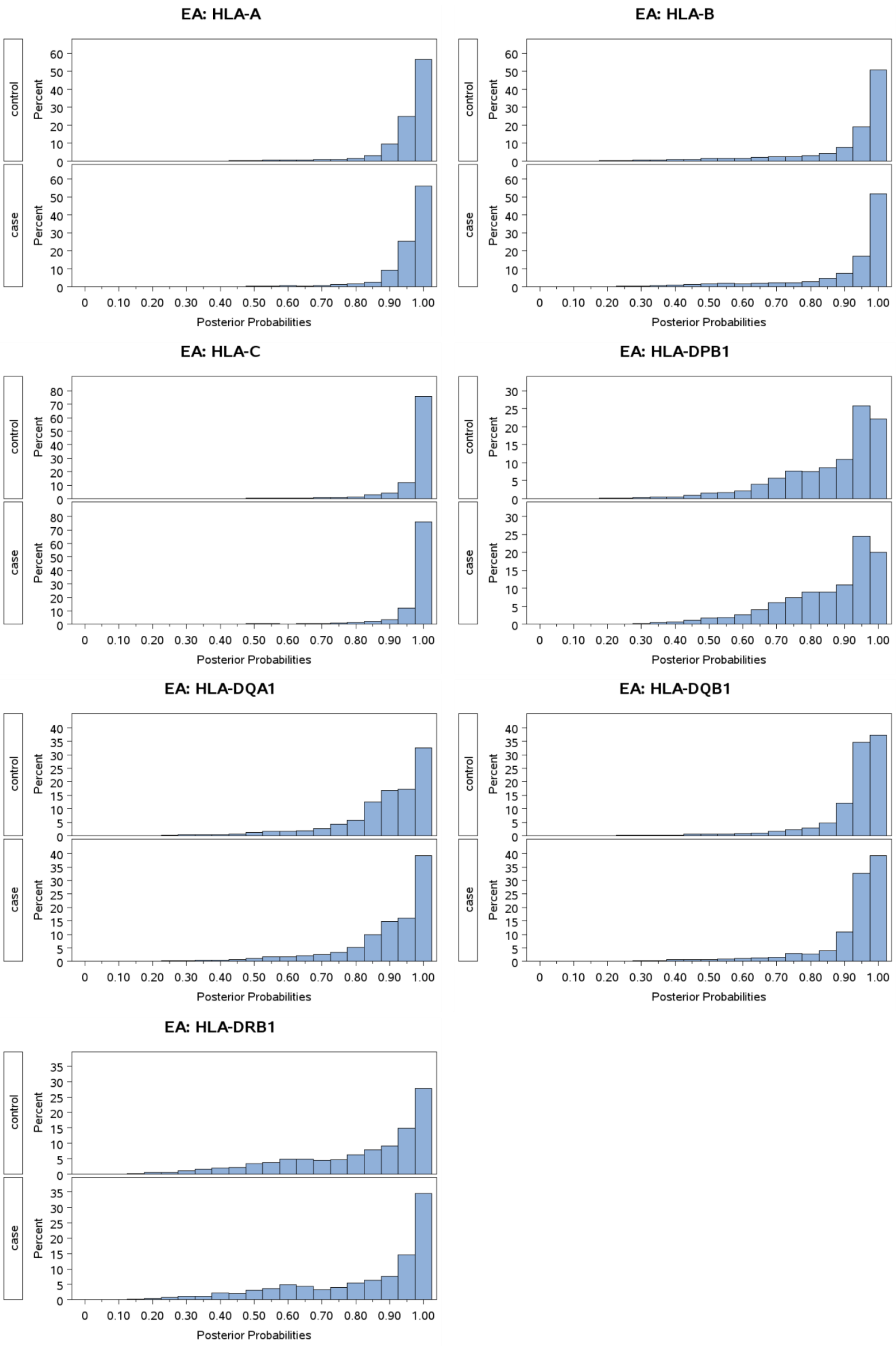

b. African American

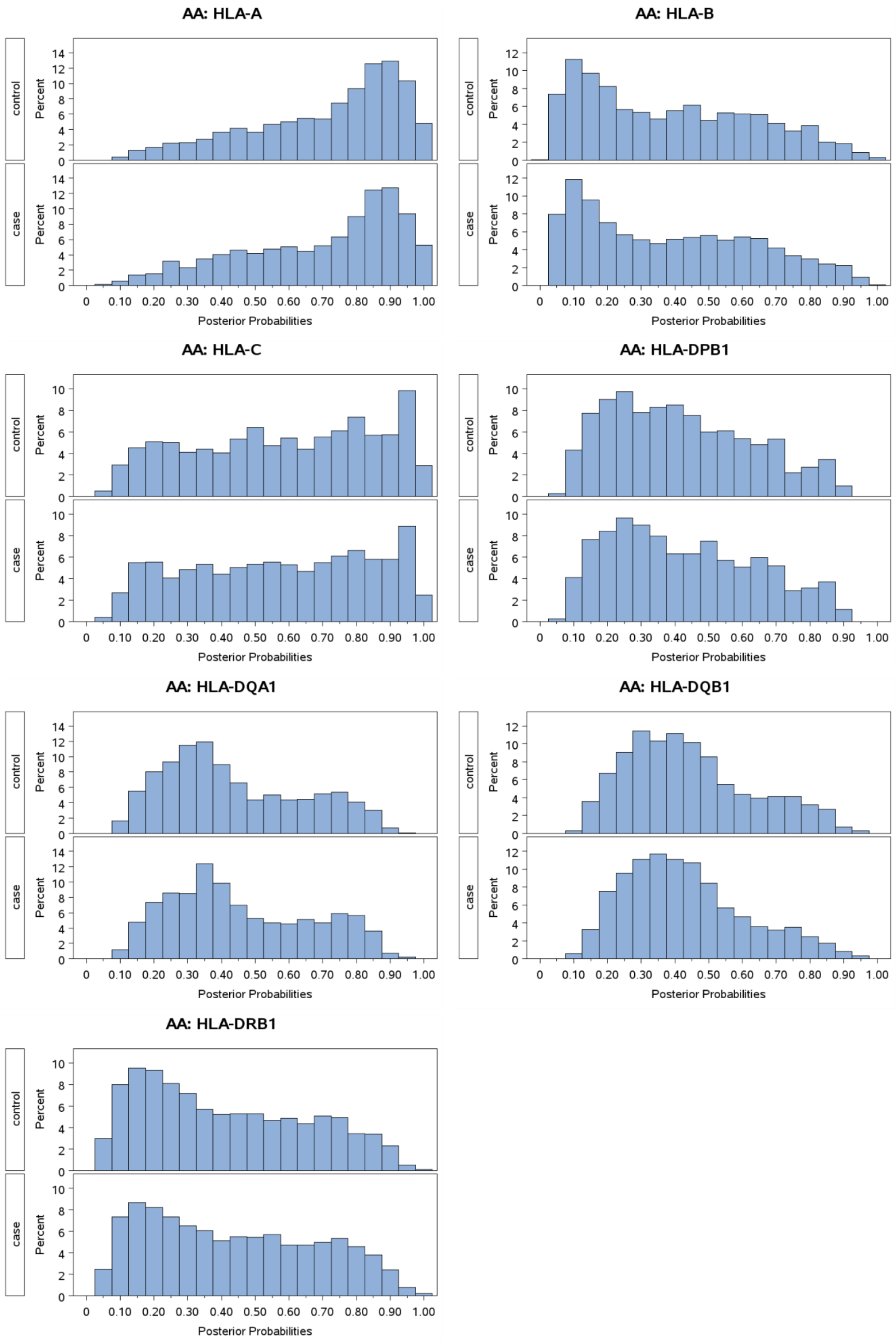

c. Hispanic Ancestry

HA: HLA-A

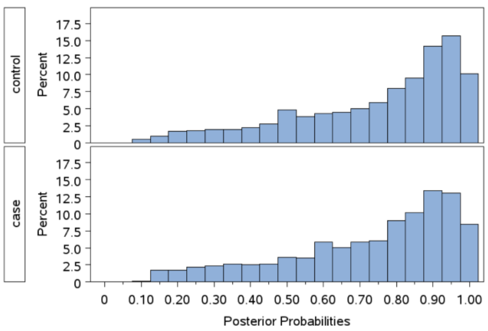

HA: HLA-B

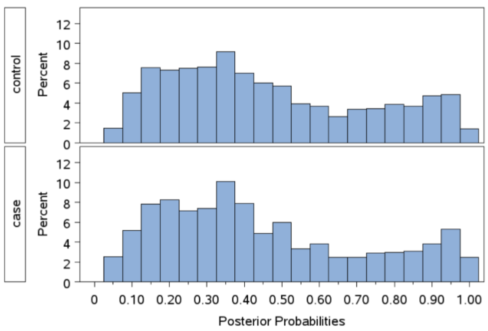

HA: HLA-C

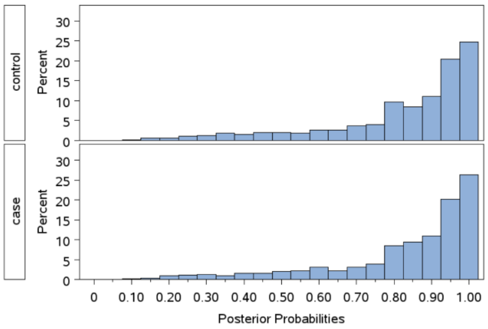

HA: HLA-DPB1

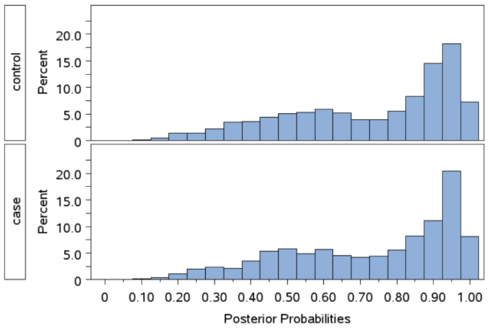

HA: HLA-DQA1

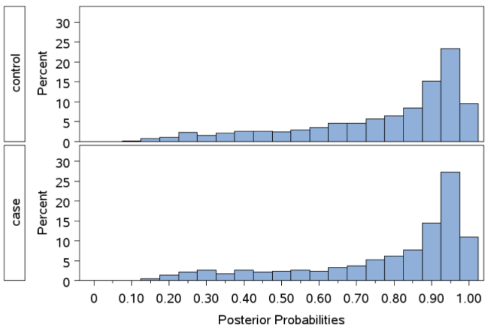

HA: HLA-DQB1

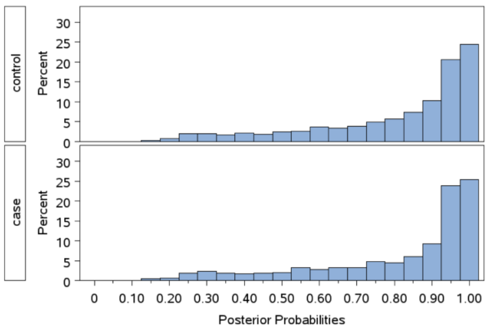

HA: HLA-DRB1

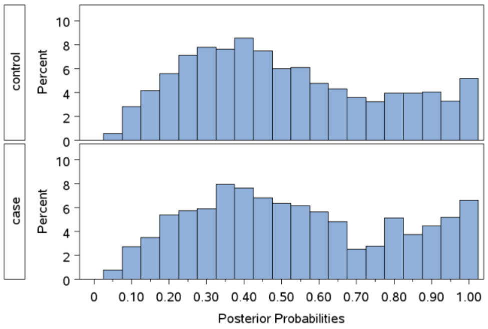

Supplementary Figure 6. Plots of SNP associations meeting genome-wide significance, post-adjustment for Classical HLA alleles.

a. European Ancestry

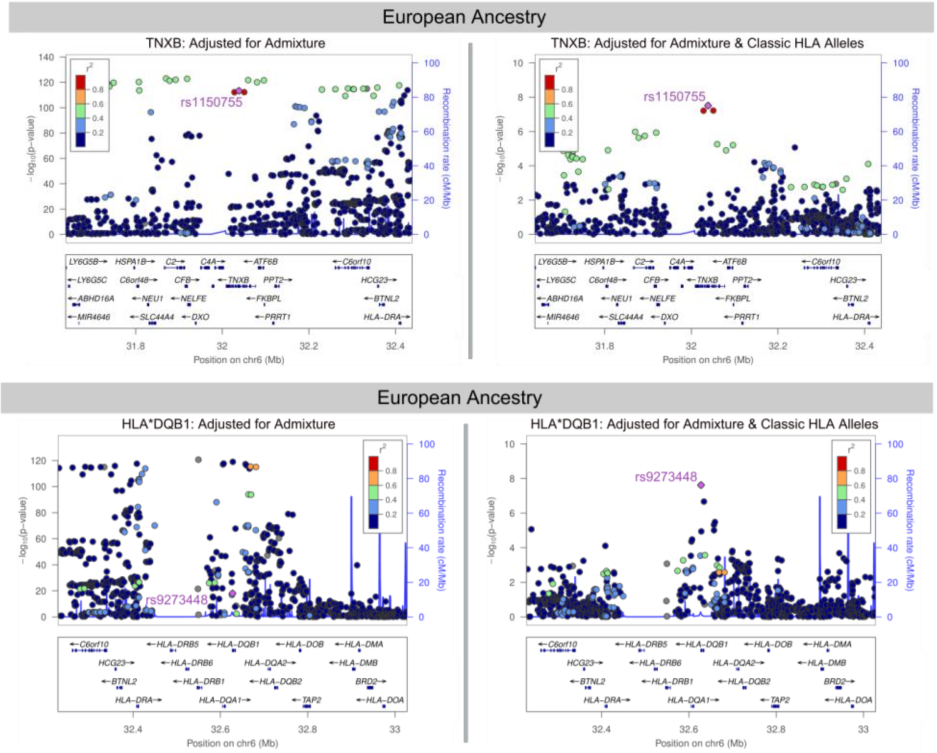

b. Meta Analysis

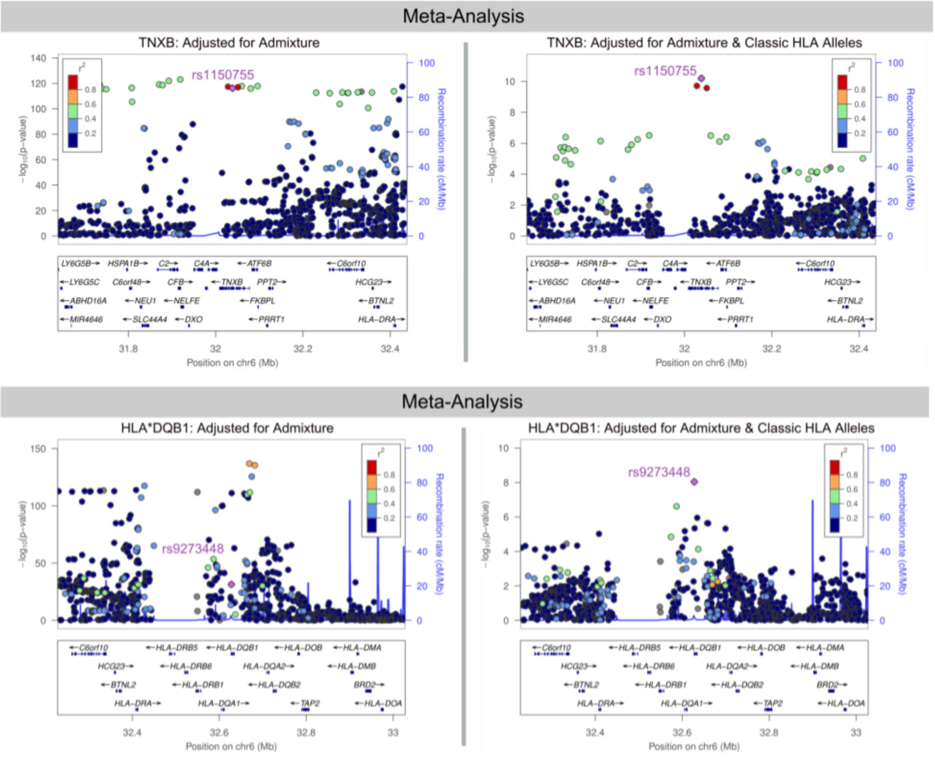

Supplementary Figure 7. Clustering of HLA alleles by amino acid sequence similarity. For each HLA gene, the odds ratios for each cohort are overlain if the SLE association p-value was less than 0.01. Alleles that were present in the multi-locus model from the stepwise procedure are also denoted. This process aims to identify clusters with shared SLE risk or not-risk odds ratios across the three cohorts. Such clusters help identify potential amino acid sequences contributing to SLE risk.

a. HLA-A

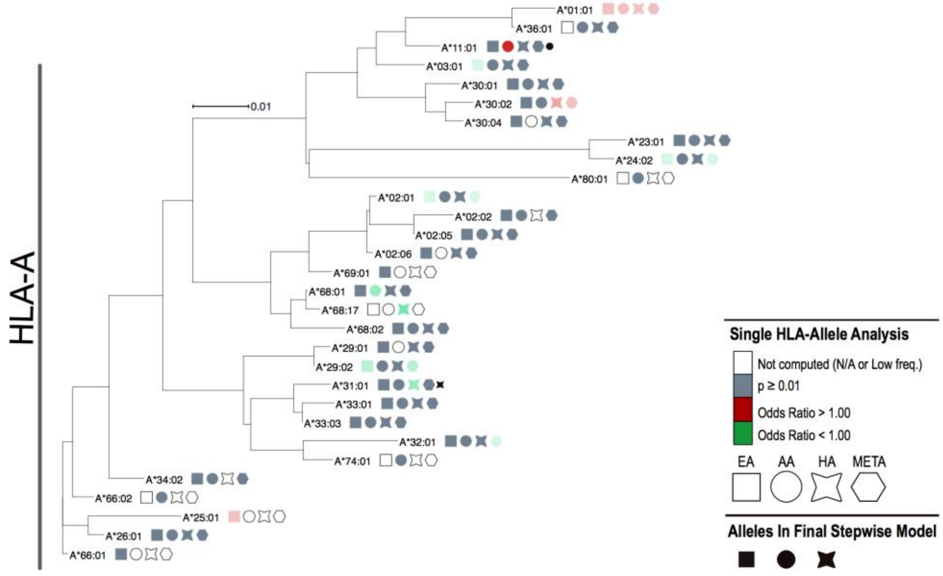

b. HLA-B

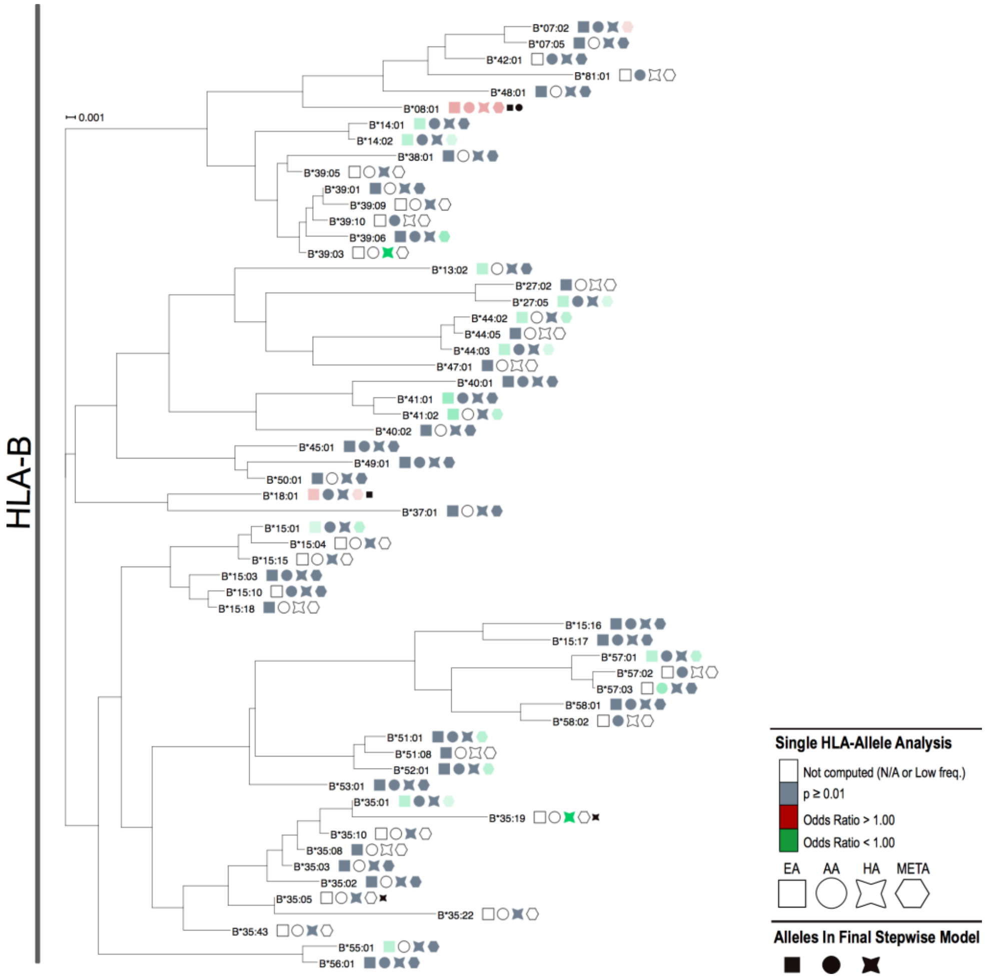

c. HLA-C

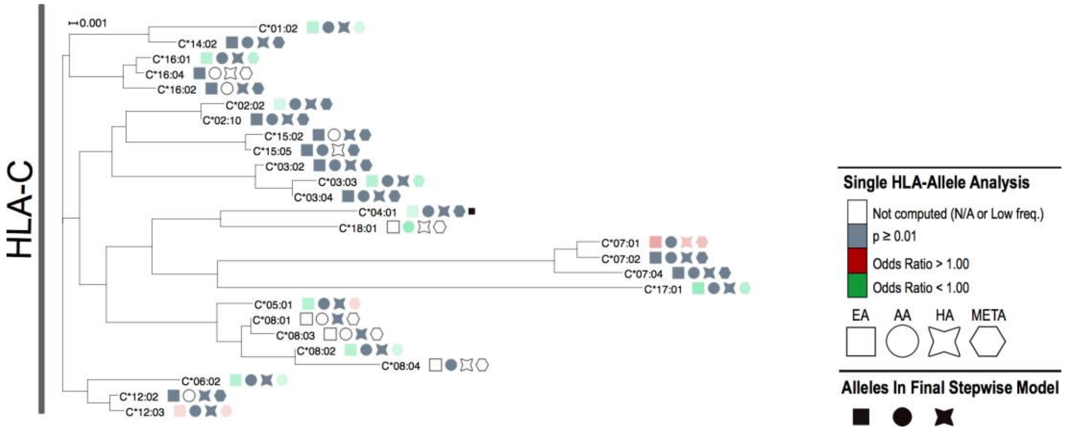

d. HLA-DPB1

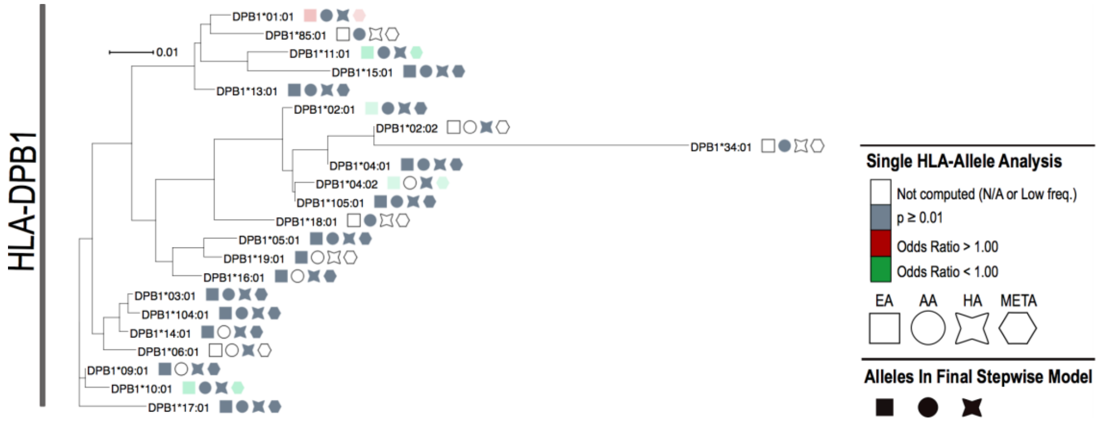

Supplementary Figure 8. Amino acids within DRB1*15 SLE risk cluster. (a) Multiple sequence alignment performed by CLUSTAL-omega of HLA-DRB1 alleles with a Percent Identity score of at least 95 with HLA-DRB1*15:01 or 15:03, two alleles associated with SLE risk in at least three populations (Figure 3). Numbering begins after signal peptide. Amino acids unique to the two risk alleles (positions -1, 47, and 71) are highlighted in purple. Positions in which the two risk alleles share an amino acid with less than half of the non-risk peptides in this cluster are highlighted in green. (b) Three dimensional ribbon model of HLA-DR protein. This structure is based on Protein Data Bank entry 3pdo. Amino acid positions noted in (a) are highlighted in red. (c) An enlarged view of the HLA-DRβ subunit, rotated for another view of noted amino acids in binding pocket.

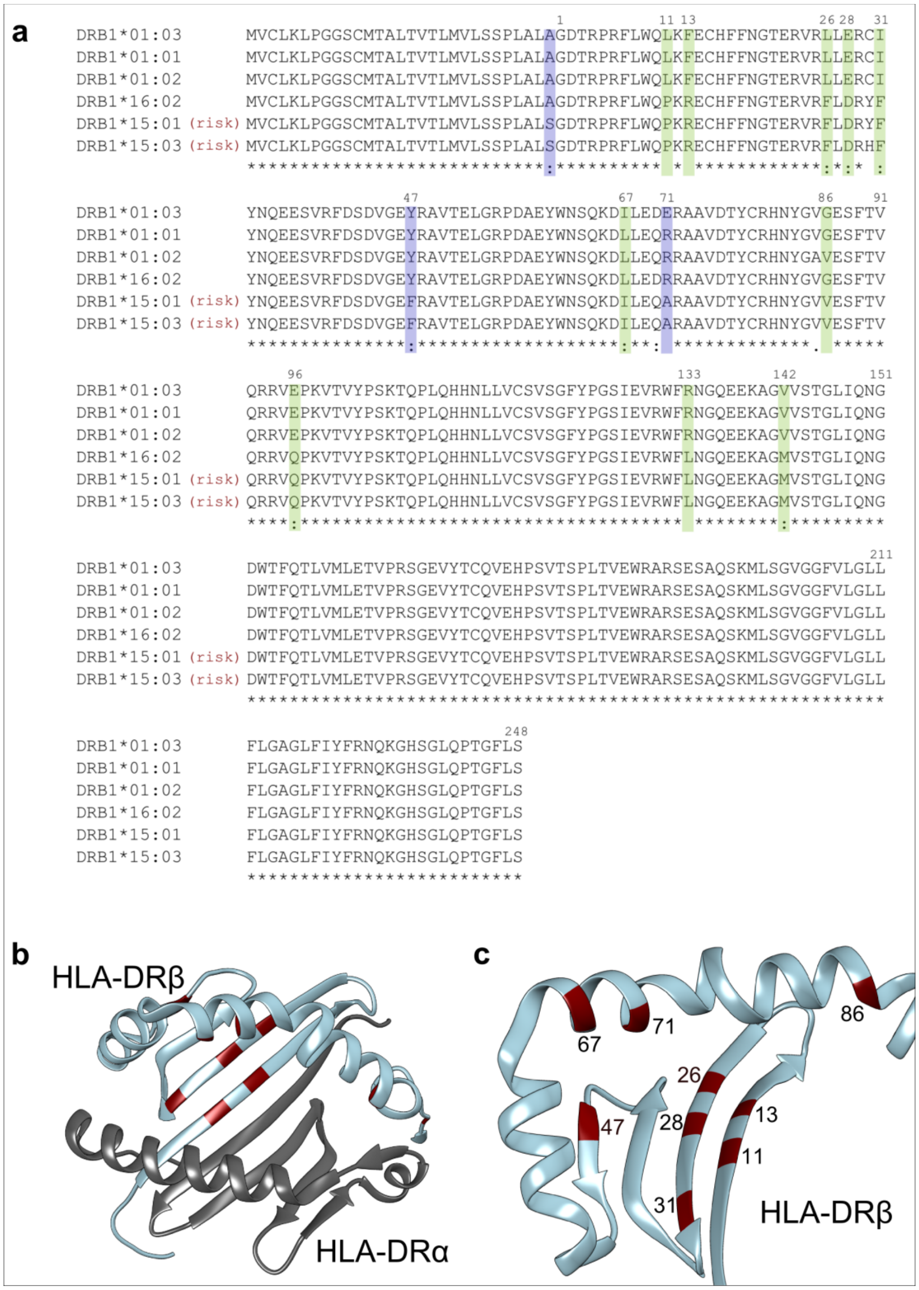

Supplementary Figure 9. Three dimensional ribbon model of classical HLA alleles. Three dimensional ribbon model of classical HLA alleles representative of binding-pocket amino acids that distinguish among risk and non-risk alleles in the DRB1*01; 16; 15 cluster (Figure 3a, Supplementary figure 10.) Non-polar, hydrophobic Phe47 Ala71 (Pocket 7) residues are unique to the two risk alleles (DRB1*15:01 and 15:03) within this cluster. Contrastingly, the non-risk alleles (DRB1*01:03, 01:01, 01:02, 16:02) exhibit a polar Tyr47 and either a polar Arg71 or polar Glu71. Focusing on amino acids sequences with at least 95% identity, allows for the ability to isolate potential regions of biochemical influence on SLE risk. In this case, SLE risk alleles DRB1*15:01 and 15:03 are unique from non-risk alleles via a hydrophobic space in DRB1 pocket 7. Proteins were modeled with SWISS-MODEL using Protein Data Bank Entry 3pdo.1.B as the template. 3-D visualizations were created via Chimera.

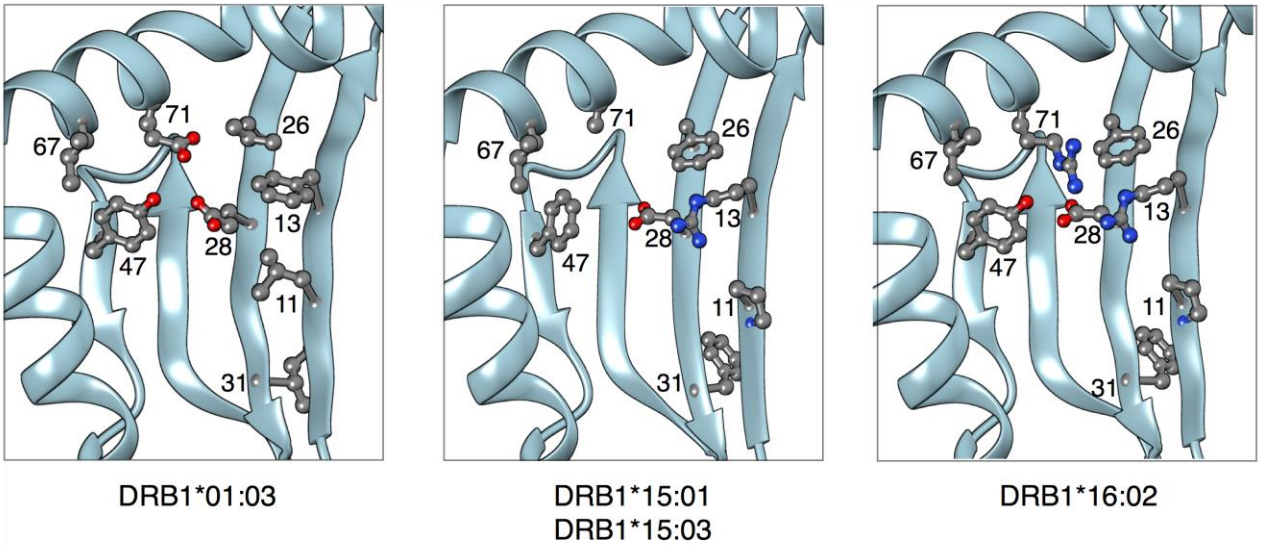

Supplementary Figure 10. DRB1*03 cluster sequence alignment. (a) Multiple sequence alignment performed by CLUSTAL-omega of HLA-DRB1 alleles with a Percent Identity score of at least 95 with SLE risk allele, HLA-DRB1*03:01. Numbering begins after signal peptide. DRB1*03:01 uniquely holds a polar Tyr26, compared to the non-polar Phe26 of the non-risk alleles within this cluster. Positions in which the risk allele shared an amino acid with 50% or less of the non-risk peptides in this cluster are highlighted in green. Most of these amino acids spanned the designated 'Shared Epitope Region'. (b) Three-dimensional representation of the DR and DRB1 protein. Amino acids of interest from the sequence alignment are highlighted in the models which were created using Chimera.

```
                                                 1                        26   31
a DRB1*14:01        MVCLRLPGGSCMAVLTVTLMVLSSPLALAGDTRPRFLEYSTSECHFFNGTERVRFLDRYF
  DRB1*03:01 (risk) MVCLRLPGGSCMAVLTVTLMVLSSPLALAGDTRPRFLEYSTSECHFFNGTERVRYLDRYF
  DRB1*03:02        -----------------------------GDTRPRFLEYSTSECHFFNGTERVRFLERYF
  DRB1*14:02        MVCLRLPGGSCMAVLTVTLMVLSSPLALAGDTRPRFLEYSTSECHFFNGTERVRFLERYF
  DRB1*13:01        MVCLRLPGGSCMAVLTVTLMVLSSPLALAGDTRPRFLEYSTSECHFFNGTERVRFLDRYF
  DRB1*11:03        MVCLRLPGGSCMAVLTVTLMVLSSPLALAGDTRPRFLEYSTSECHFFNGTERVRFLDRYF
  DRB1*11:01        MVCLRLPGGSCMAVLTVTLMVLSSPLALAGDTRPRFLEYSTSECHFFNGTERVRFLDRYF
  DRB1*11:04        MVCLRLPGGSCMAVLTVTLMVLSSPLALAGDTRPRFLEYSTSECHFFNGTERVRFLDRYF
                                                 *************************:*:***

                         37                                70 73   77             91
  DRB1*14:01        HNQEEFVRFDSDVGEYRAVTELGRPAAEHWNSQKDLLERRRAEVDTYCRHNYGVVESFTV
  DRB1*03:01 (risk) HNQEENVRFDSDVGEFRAVTELGRPDAEYWNSQKDLLEQKRGRVDNYCRHNYGVVESFTV
  DRB1*03:02        HNQEENVRFDSDVGEYRAVTELGRPDAEYWNSQKDLLEQKRGRVDNYCRHNYGVGESFTV
  DRB1*14:02        HNQEENVRFDSDVGEYRAVTELGRPDAEYWNSQKDLLEQRRAAVDTYCRHNYGVGESFTV
  DRB1*13:01        HNQEENVRFDSDVGEFRAVTELGRPDAEYWNSQKDILEDERAAVDTYCRHNYGVVESFTV
  DRB1*11:03        YNQEEYVRFDSDVGEFRAVTELGRPDEEYWNSQKDFLEDERAAVDTYCRHNYGVVESFTV
  DRB1*11:01        YNQEEYVRFDSDVGEFRAVTELGRPDEEYWNSQKDFLEDRRAAVDTYCRHNYGVGESFTV
  DRB1*11:04        YNQEEYVRFDSDVGEFRAVTELGRPDEEYWNSQKDFLEDRRAAVDTYCRHNYGVVESFTV
                    :**** *********:*********  *:******:** .*. **.******** *****

                                                                              151
  DRB1*14:01        QRRVHPKVTVYPSKTQPLQHYNLLVCSVSGFYPGSIEVRWFRNGQEEKTGVVSTGLIHNG
  DRB1*03:01 (risk) QRRVHPKVTVYPSKTQPLQHHNLLVCSVSGFYPGSIEVRWFRNGQEEKTGVVSTGLIHNG
  DRB1*03:02        QRRVHPKVTVYPSKTQPLQHHNLLVCSVSGFYPGSIEVRWFRNGQEEKTGVVSTGLIHNG
  DRB1*14:02        QRRVHPKVTVYPSKTQPLQHHNLLVCSVSGFYPGSIEVRWFRNGQEEKTGVVSTGLIHNG
  DRB1*13:01        QRRVHPKVTVYPSKTQPLQHHNLLVCSVSGFYPGSIEVRWFRNGQEEKTGVVSTGLIHNG
  DRB1*11:03        QRRVHPKVTVYPSKTQPLQHHNLLVCSVSGFYPGSIEVRWFRNGQEEKTGVVSTGLIHNG
  DRB1*11:01        QRRVHPKVTVYPSKTQPLQHHNLLVCSVSGFYPGSIEVRWFRNGQEEKTGVVSTGLIHNG
  DRB1*11:04        QRRVHPKVTVYPSKTQPLQHHNLLVCSVSGFYPGSIEVRWFRNGQEEKTGVVSTGLIHNG
                    ********************:***************************************

                                                                              211
  DRB1*14:01        DWTFQTLVMLETVPRSGEVYTCQVEHPSVTSPLTVEWRARSESAQSKMLSGVGGFVLGLL
  DRB1*03:01 (risk) DWTFQTLVMLETVPRSGEVYTCQVEHPSVTSPLTVEWRARSESAQSKMLSGVGGFVLGLL
  DRB1*03:02        DWTFQTLVMLETVPRSGEVYTCQVEHPSVTSPLTVEWRARSESAQSKMLSGVGGFVLGLL
  DRB1*14:02        DWTFQTLVMLETVPRSGEVYTCQVEHPSVTSPLTVEWRARSESAQSKMLSGVGGFVLGLL
  DRB1*13:01        DWTFQTLVMLETVPRSGEVYTCQVEHPSVTSPLTVEWRARSESAQSKMLSGVGGFVLGLL
  DRB1*11:03        DWTFQTLVMLETVPRSGEVYTCQVEHPSVTSPLTVEWRARSESAQSKMLSGVGGFVLGLL
  DRB1*11:01        DWTFQTLVMLETVPRSGEVYTCQVEHPSVTSPLTVEWRARSESAQSKMLSGVGGFVLGLL
  DRB1*11:04        DWTFQTLVMLETVPRSGEVYTCQVEHPSVTSPLTVEWRARSESAQSKMLSGVGGFVLGLL
                    ************************************************************

                                            248
  DRB1*14:01        FLGAGLFIYFRNQKGHSGLQPRGFLS
  DRB1*03:01 (risk) FLGAGLFIYFRNQKGHSGLQPRGFLS
  DRB1*03:02        FLGAGLFIYFRNQKGHSGLQPRGFLS
  DRB1*14:02        FLGAGLFIYFRNQKGHSGLQPRGF--
  DRB1*13:01        FLGAGLFIYFRNQKGHSGLQPRGFLS
  DRB1*11:03        FLGAGLFIYFRNQKGHSGLQPRGFLS
  DRB1*11:01        FLGAGLFIYFRNQKGHSGLQPRGFLS
  DRB1*11:04        FLGAGLFIYFRNQKGHSGLQPRGFLS
                    ************************
```

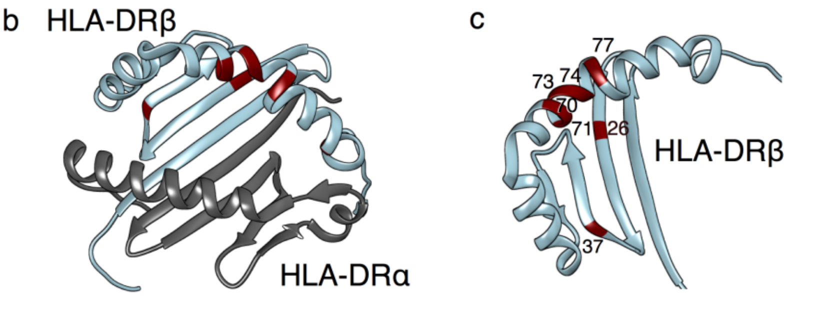

Supplementary Figure 11. Sequence Alignment of DQA1*01 cluster. Sequence alignment comparing SLE risk allele DQA1*01:02 to non-risk alleles with 95% sequence identity (DQA1*01:03, *01:01, *01:04, and *01:05). Met207 is the only amino acid unique to risk allele DQA1*01:02 from it's non-risk neighbors. Positions in which the risk allele shared an amino acid with 50% or less of the non-risk peptides in this cluster are highlighted in green (Gln34).

```
                                                1                               34  37
DQA1*01:03       MILNKALLLGALALTTVMSPCGGEDIVADHVASCGVNLYQFYGPSGQFTHEFDGDEQFYV
DQA1*01:02(risk) MILNKALLLGALALTTVMSPCGGEDIVADHVASCGVNLYQFYGPSGQYTHEFDGDEQFYV
DQA1*01:01       MILNKALLLGALALTTVMSPCGGEDIVADHVASCGVNLYQFYGPSGQYTHEFDGDEEFYV
DQA1*01:04       MILNKALLLGALALTTMMSPCGGEGIVADHVASCGVNLYQFYGPSGQYTHEFDGDEEFYV
DQA1*01:05       MILNKALLLGALALTTMMSPCGGEGIVADHVASCGVNLYQFYGPSGQYTHEFDGDEEFYV
                 ****************:******* **********************:********:***

                                                                            97
DQA1*01:03       DLEKKETAWRWPEFSKFGGFDPQGALRNMAVAKHNLNIMIKRYNSTAATNEVPEVTVFSK
DQA1*01:02(risk) DLERKETAWRWPEFSKFGGFDPQGALRNMAVAKHNLNIMIKRYNSTAATNEVPEVTVFSK
DQA1*01:01       DLERKETAWRWPEFSKFGGFDPQGALRNMAVAKHNLNIMIKRYNSTAATNEVPEVTVFSK
DQA1*01:04       DLERKETAWRWPEFSKFGGFDPQGALRNMAVAKHNLNIMIKRYNSTAATNEVPEVTVFSK
DQA1*01:05       DLERKETAWRWPEFSKFGGFDPQGALRNMAVAKHNLNIMIKRYNSTAATNEVPEVTVFSK
                 ***:********************************************************

                                                                           157
DQA1*01:03       SPVTLGQPNTLICLVDNIFPPVVNITWLSNGHAVTEGVSETSFLSKSDHSFFKISYLTFL
DQA1*01:02(risk) SPVTLGQPNTLICLVDNIFPPVVNITWLSNGQSVTEGVSETSFLSKSDHSFFKISYLTFL
DQA1*01:01       SPVTLGQPNTLICLVDNIFPPVVNITWLSNGQSVTEGVSETSFLSKSDHSFFKISYLTFL
DQA1*01:04       SPVTLGQPNTLICLVDNIFPPVVNITWLSNGQSVTEGVSETSFLSKSDHSFFKISYLTFL
DQA1*01:05       SPVTLGQPNTLICLVDNIFPPVVNITWLSNGQSVTEGVSETSFLSKSDHSFFKISYLTFL
                 *******************************::***************************

                                                                 207       217
DQA1*01:03       PSADEIYDCKVEHWGLDQPLLKHWEPEIPAPMSELTETVVCALGLSVGLVGIVVGTVFII
DQA1*01:02(risk) PSADEIYDCKVEHWGLDQPLLKHWEPEIPAPMSELTETVVCALGLSVGLMGIVVGTVFII
DQA1*01:01       PSADEIYDCKVEHWGLDQPLLKHWEPEIPAPMSELTETVVCALGLSVGLVGIVVGTVFII
DQA1*01:04       PSADEIYDCKVEHWGLDQPLLKHWEPEIPAPMSELTETVVCTLGLSVGLVGIVVGTVFII
DQA1*01:05       PSADEIYDCKVEHWGLDQPLLKHWEPEIPAPMSELTETVVCALGLSVGLVGIVVGTVFII
                 *****************************************:*******:**********

                             217
DQA1*01:03       QGLRSVGASRHQGPL
DQA1*01:02(risk) QGLRSVGASRHQGPL
DQA1*01:01       QGLRSVGASRHQGPL
DQA1*01:04       QGLRSVGASRHQGPL
DQA1*01:05       QGLRSVGASRHQGPL
                 ***************
```

Supplementary Figure 12. Sequence alignment of DQA1*05 cluster. Sequence alignment comparing SLE risk allele DQA1*05:01 to non-risk alleles with 95% sequence identity (DQA1*05:05 and *05:03). Thr-13 and Ala159 are the only differences among these three alleles are highlighted below.

```
                          -13            1                                  37
DQA1*05:05        MILNKALMLGTLALTTVMSPCGGEDIVADHVASYGVNLYQSYGPSGQYTHEFDGDEQFYV
DQA1*05:03        MILNKALMLGALALTTVMSPCGGEDIVADHVASYGVNLYQSYGPSGQYTHEFDGDEQFYV
DQA1*05:01 (risk) MILNKALMLGALALTTVMSPCGGEDIVADHVASYGVNLYQSYGPSGQYTHEFDGDEQFYV
                  **********:*************************************************

                                                                            97
DQA1*05:05        DLGRKETVWCLPVLRQFRFDPQFALTNIAVLKHNLNSLIKRSNSTAATNEVPEVTVFSKS
DQA1*05:03        DLGRKETVWCLPVLRQFRFDPQFALTNIAVLKHNLNSLIKRSNSTAATNEVPEVTVFSKS
DQA1*05:01 (risk) DLGRKETVWCLPVLRQFRFDPQFALTNIAVLKHNLNSLIKRSNSTAATNEVPEVTVFSKS
                  ************************************************************

                                                                            157
DQA1*05:05        PVTLGQPNILICLVDNIFPPVVNITWLSNGHSVTEGVSETSFLSKSDHSFFKISYLTLLP
DQA1*05:03        PVTLGQPNILICLVDNIFPPVVNITWLSNGHSVTEGVSETSFLSKSDHSFFKISYLTLLP
DQA1*05:01 (risk) PVTLGQPNILICLVDNIFPPVVNITWLSNGHSVTEGVSETSFLSKSDHSFFKISYLTLLP
                  ************************************************************

                                                                            217
DQA1*05:05        SAEESYDCKVEHWGLDKPLLKHWEPEIPAPMSELTETVVCALGLSVGLVGIVVGTVFIIR
DQA1*05:03        SSEESYDCKVEHWGLDKPLLKHWEPEIPAPMSELTETVVCALGLSVGLVGIVVGTVFIIR
DQA1*05:01 (risk) SAEESYDCKVEHWGLDKPLLKHWEPEIPAPMSELTETVVCALGLSVGLVGIVVGTVFIIR
                  *:**********************************************************

DQA1*05:05        GLRSVGASRHQGPL
DQA1*05:03        GLRSVGASRHQGPL
DQA1*05:01 (risk) GLRSVGASRHQGPL
                  **************
```

Supplementary Figure 13. Plots of novel Tier 1 meta-analysis and transancestral-mapping regions. Plots appear in the order found in Table 3a. Individual plots were created using LocusZoom standalone software (http://locuszoom.sph.umich.edu/)

a. IL12RB2

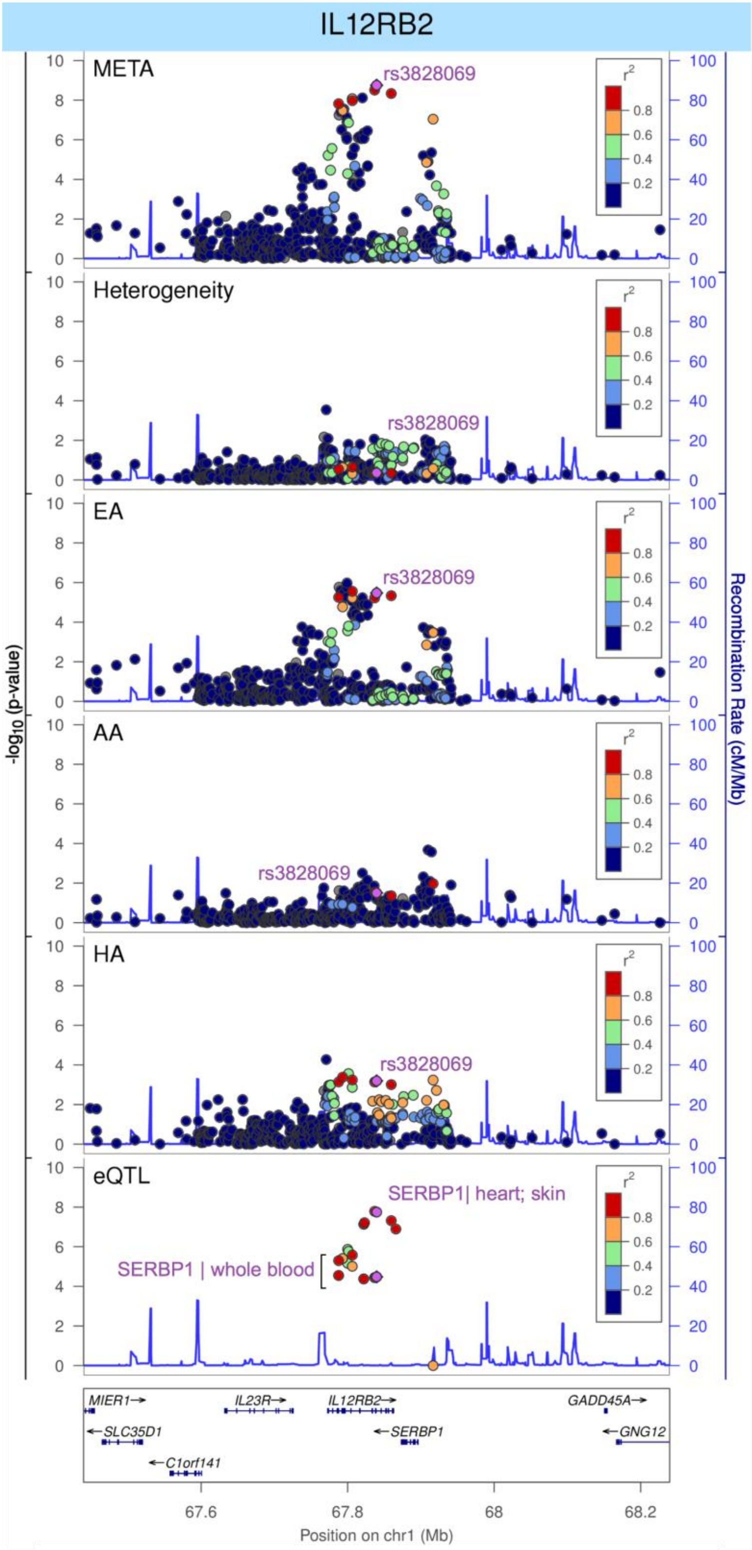

b. PAPOLG-FLJ16341

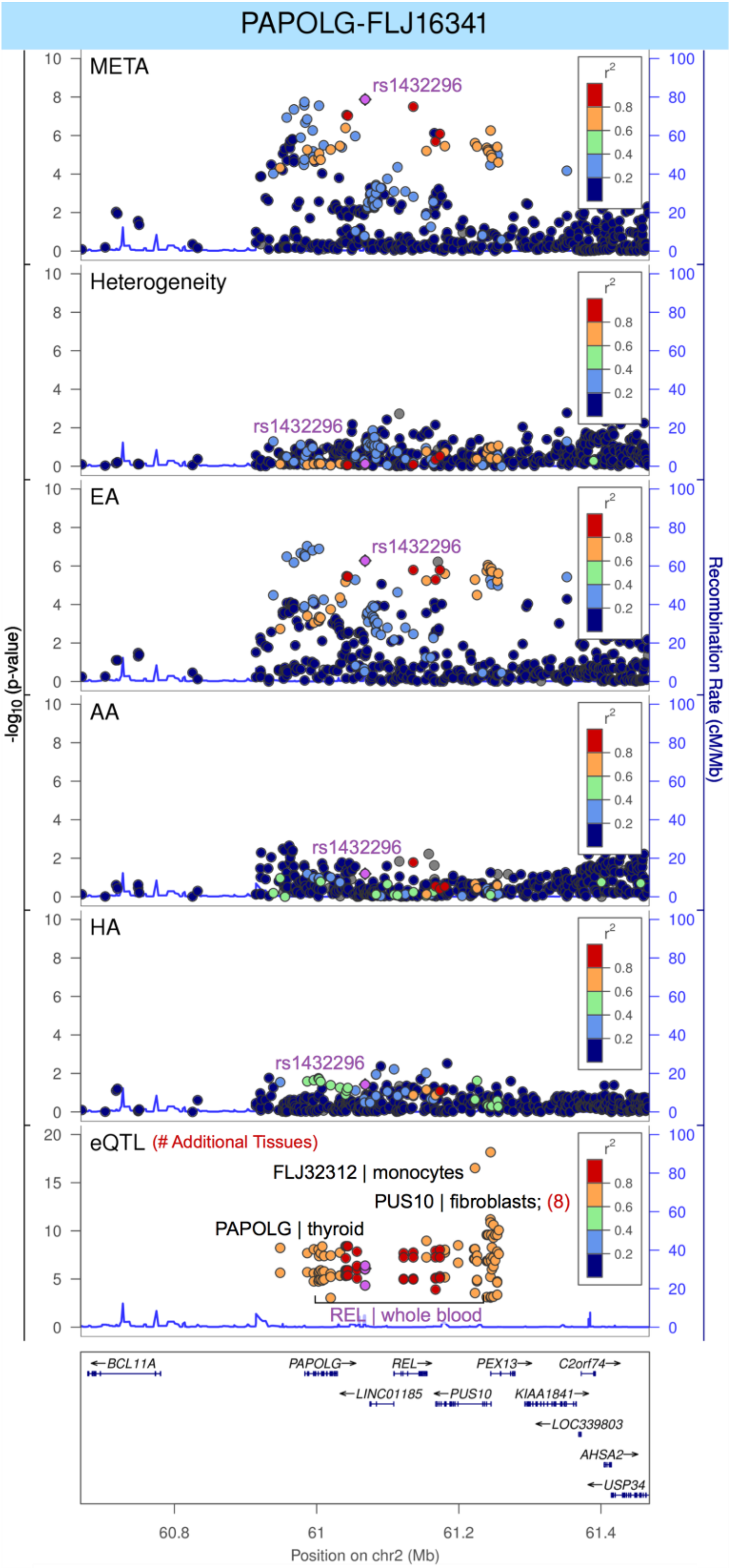

c. DGKQ

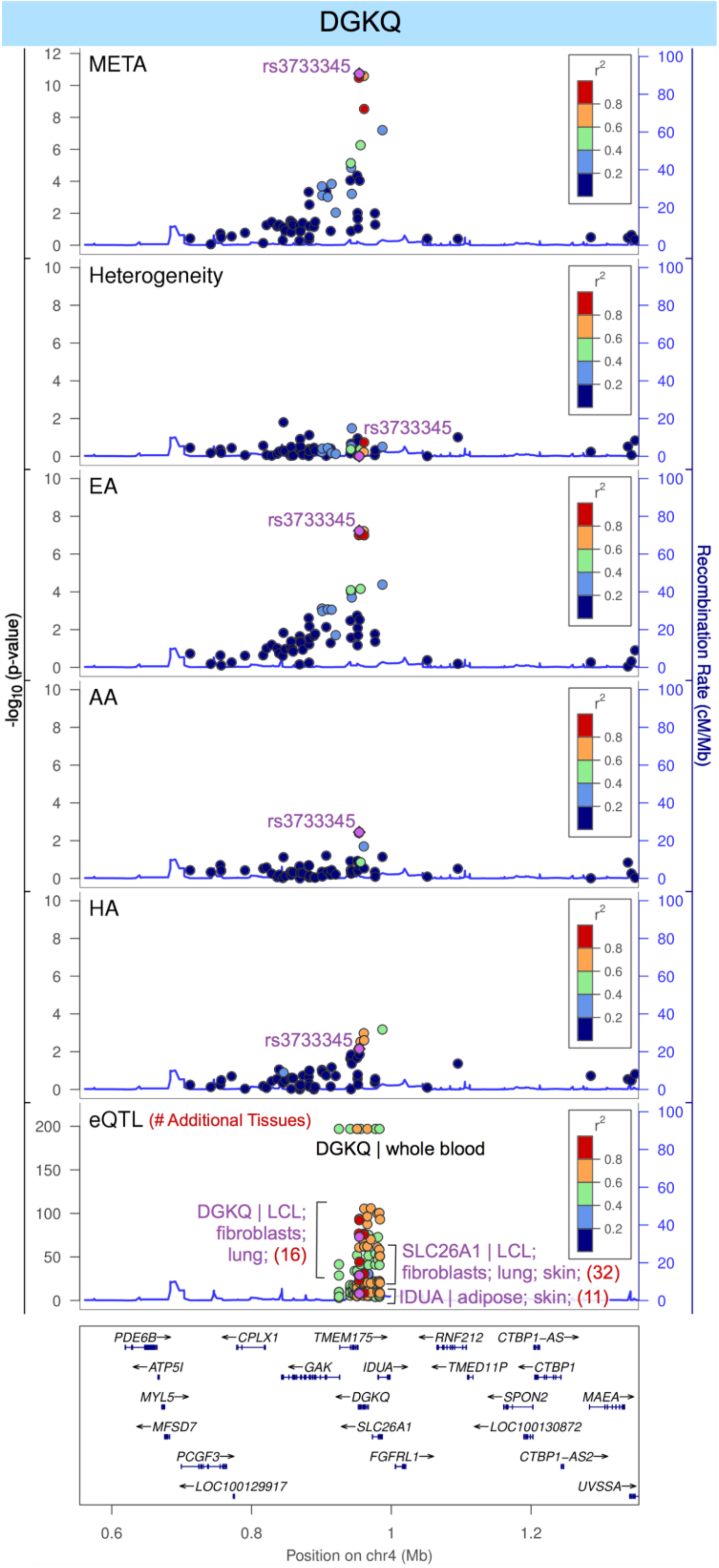
DGKQ
META
rs3733345
Heterogeneity
EA
AA
HA
eQTL (# Additional Tissues)
DGKQ | whole blood
DGKQ | LCL; fibroblasts; lung; (16)
SLC26A1 | LCL; fibroblasts; lung; skin; (32)
IDUA | adipose; skin; (11)
r^2
-log10 (p-value)
Recombination Rate (cM/Mb)
Position on chr4 (Mb)

d. ST8SIA4

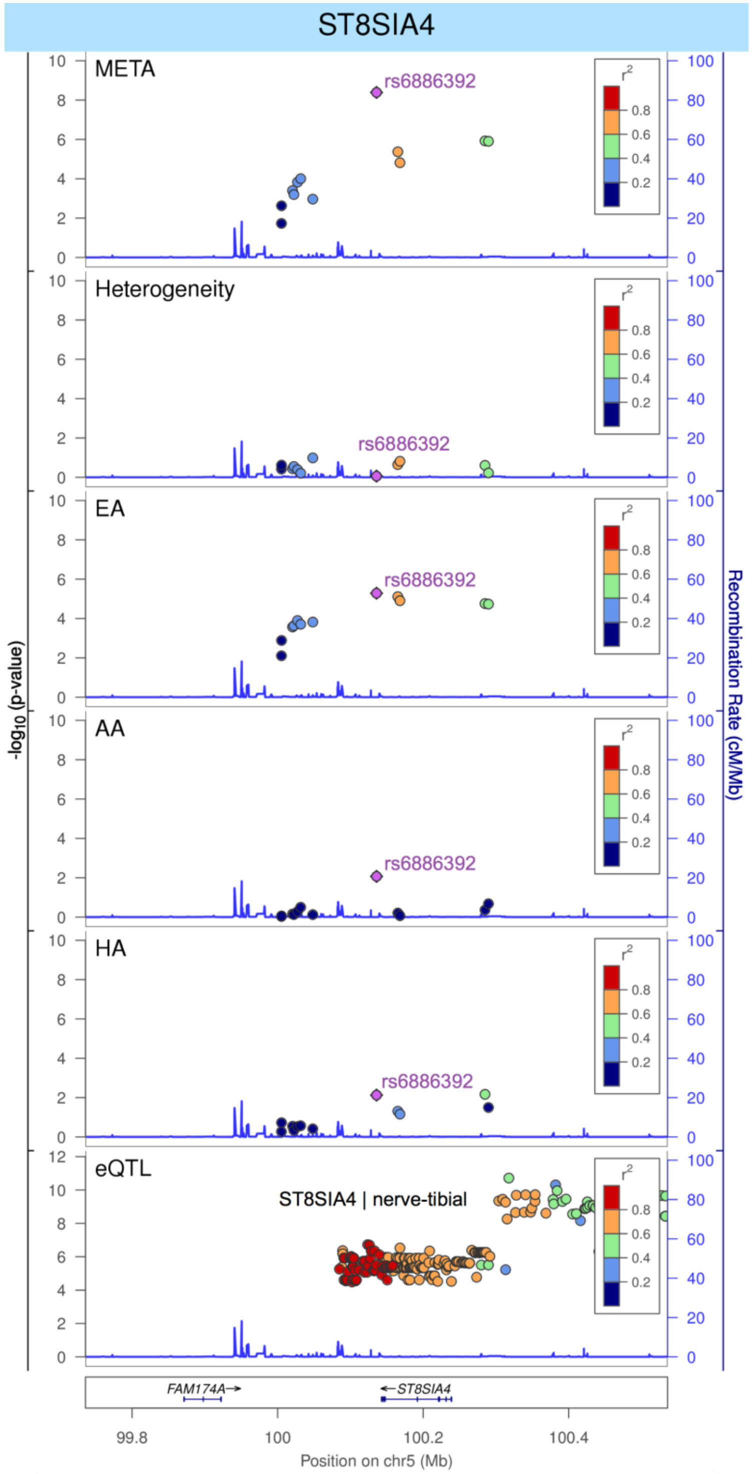

ST8SIA4
META
rs6886392
Heterogeneity
EA
AA
HA
eQTL
ST8SIA4 | nerve-tibial
r2
0.8
0.6
0.4
0.2
-log10 (p-value)
Recombination Rate (cM/Mb)
FAM174A
ST8SIA4
99.8
100
100.2
100.4
Position on chr5 (Mb)

e. UHRF1BP1-SCUBE3-DEF6 (SLE SNP associations adjusted for Classical HLA alleles)

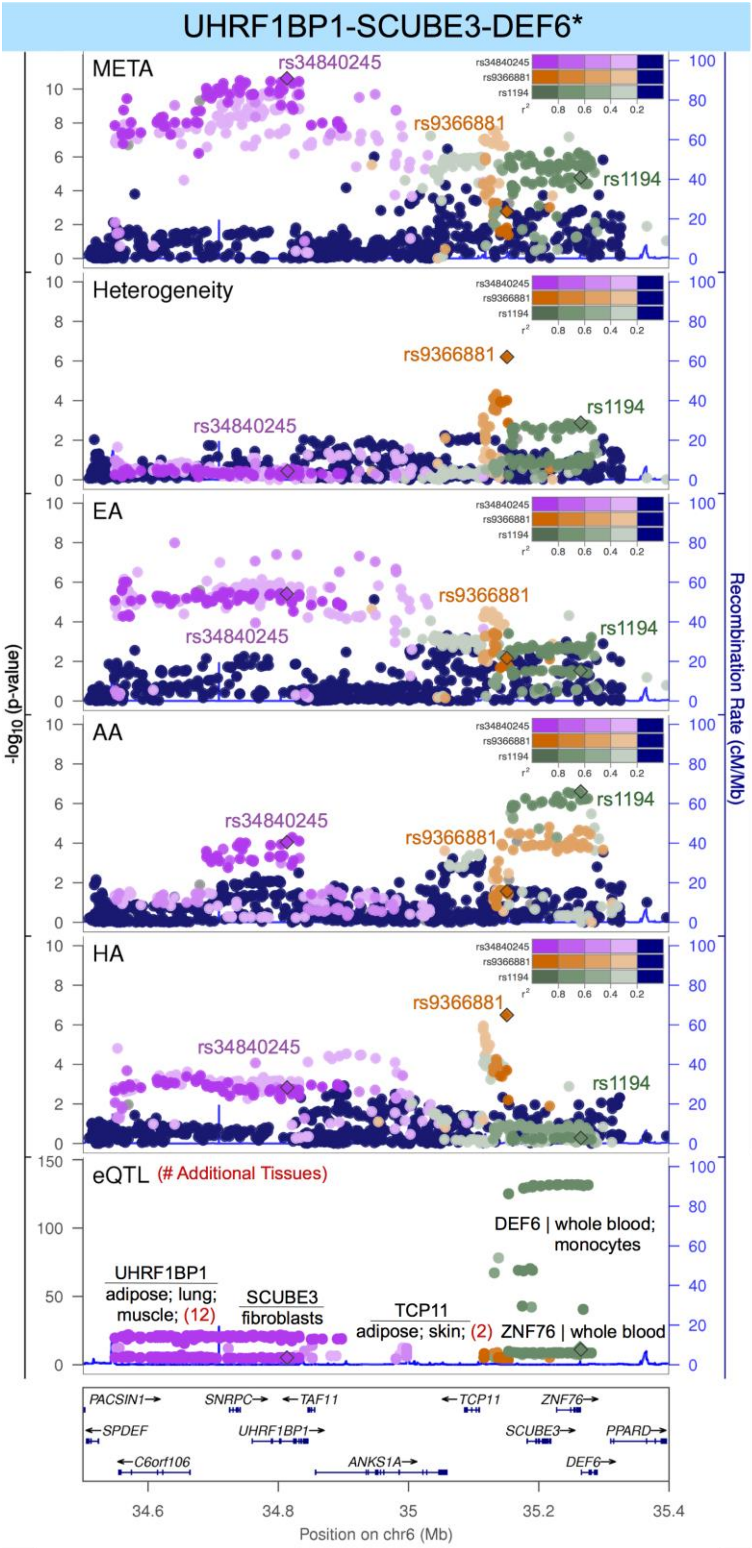

*SLE SNP associations adjusted for Classic HLA alleles

f. UHRF1BP1-SCUBE3-DEF6 (SLE SNP associations adjusted for Classical HLA alleles, and top 3 ancestry-specific SNPs: *rs34840245, rs9366881, rs1194*)

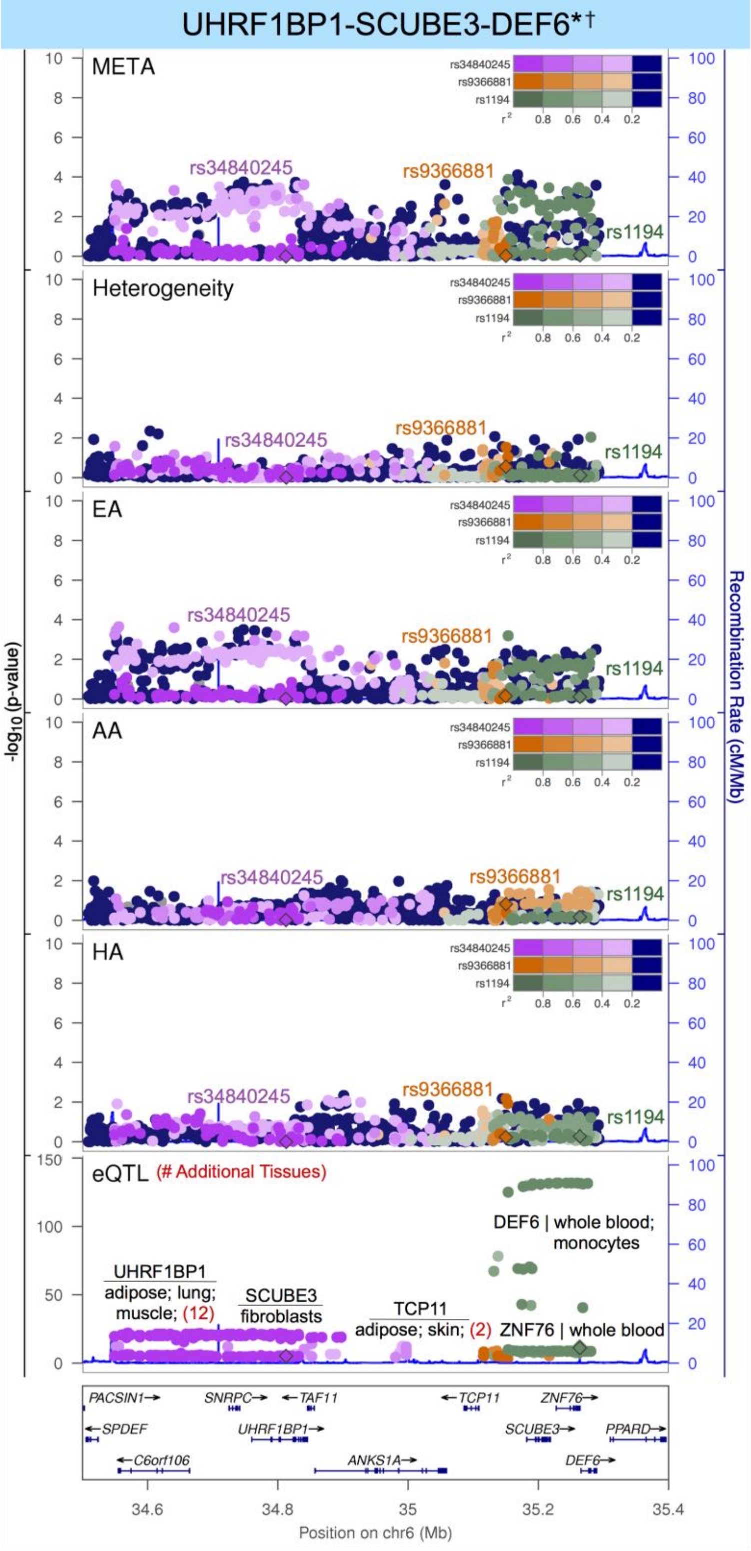

** SLE SNP associations adjusted for Classic HLA alleles*

†Post-adjustment for top 3 SNPs in region: rs34840245; rs9366881; rs1194

g. PKIA-ZC2HC1A

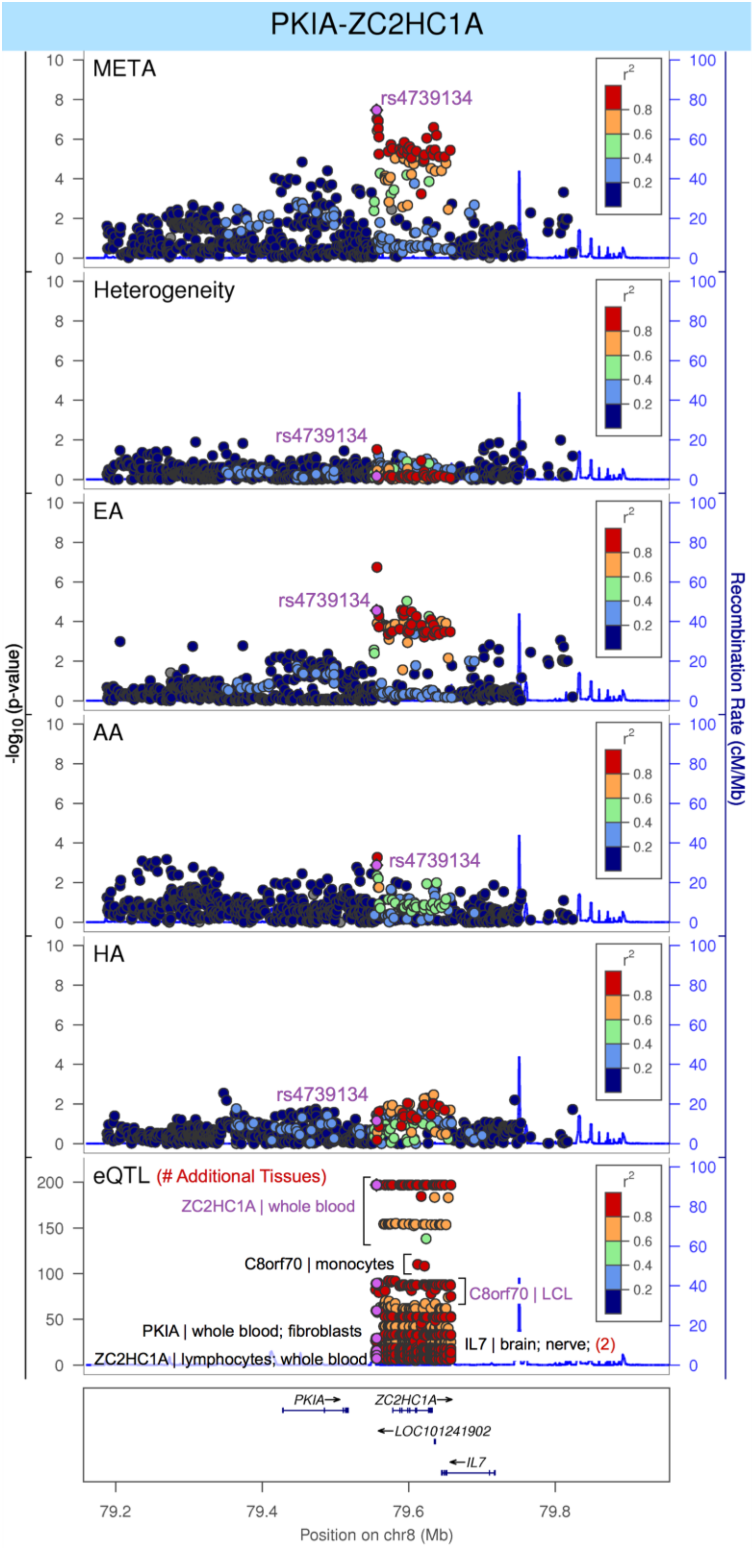

h. AK057451

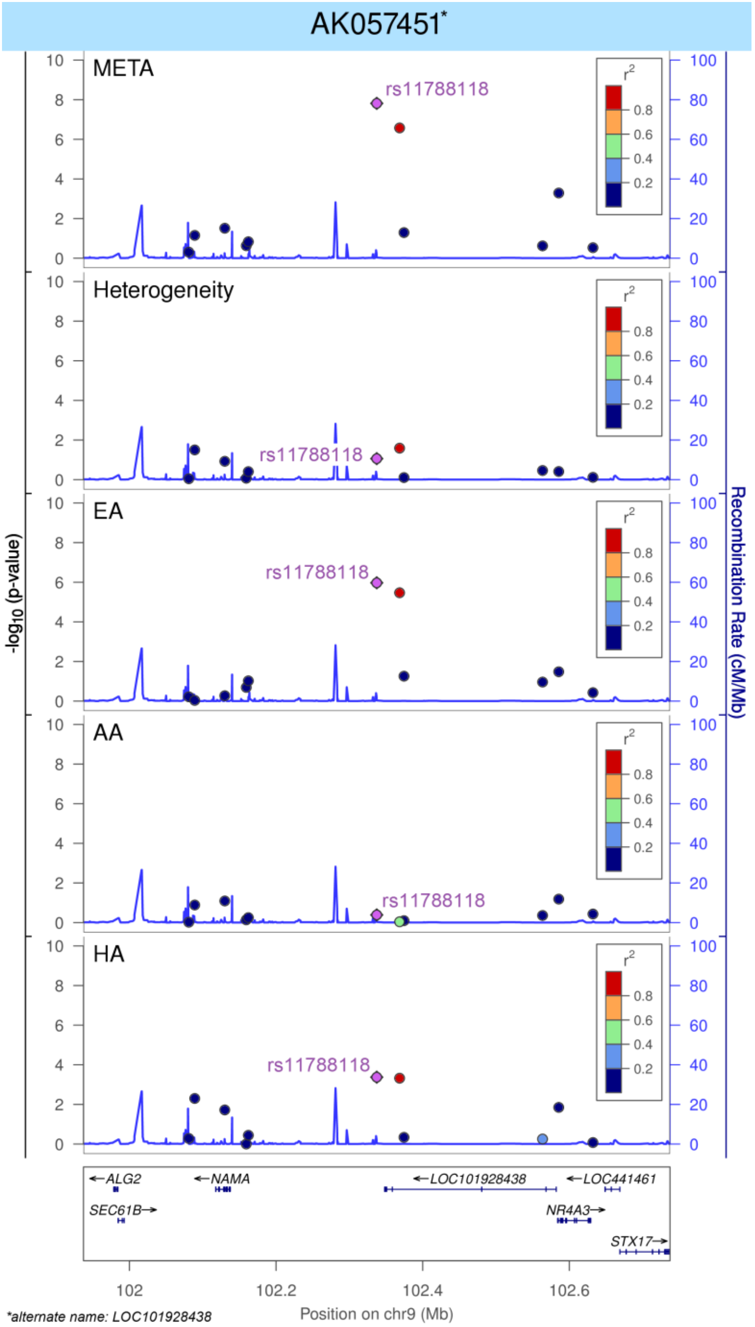
AK057451*
META
Heterogeneity
EA
AA
HA
rs11788118
r2
0.8
0.6
0.4
0.2
-log10 (p-value)
Recombination Rate (cM/Mb)
ALG2
NAMA
LOC101928438
LOC441461
SEC61B
NR4A3
STX17
102
102.2
102.4
102.6
Position on chr9 (Mb)
*alternate name: LOC101928438

i. ATXN2

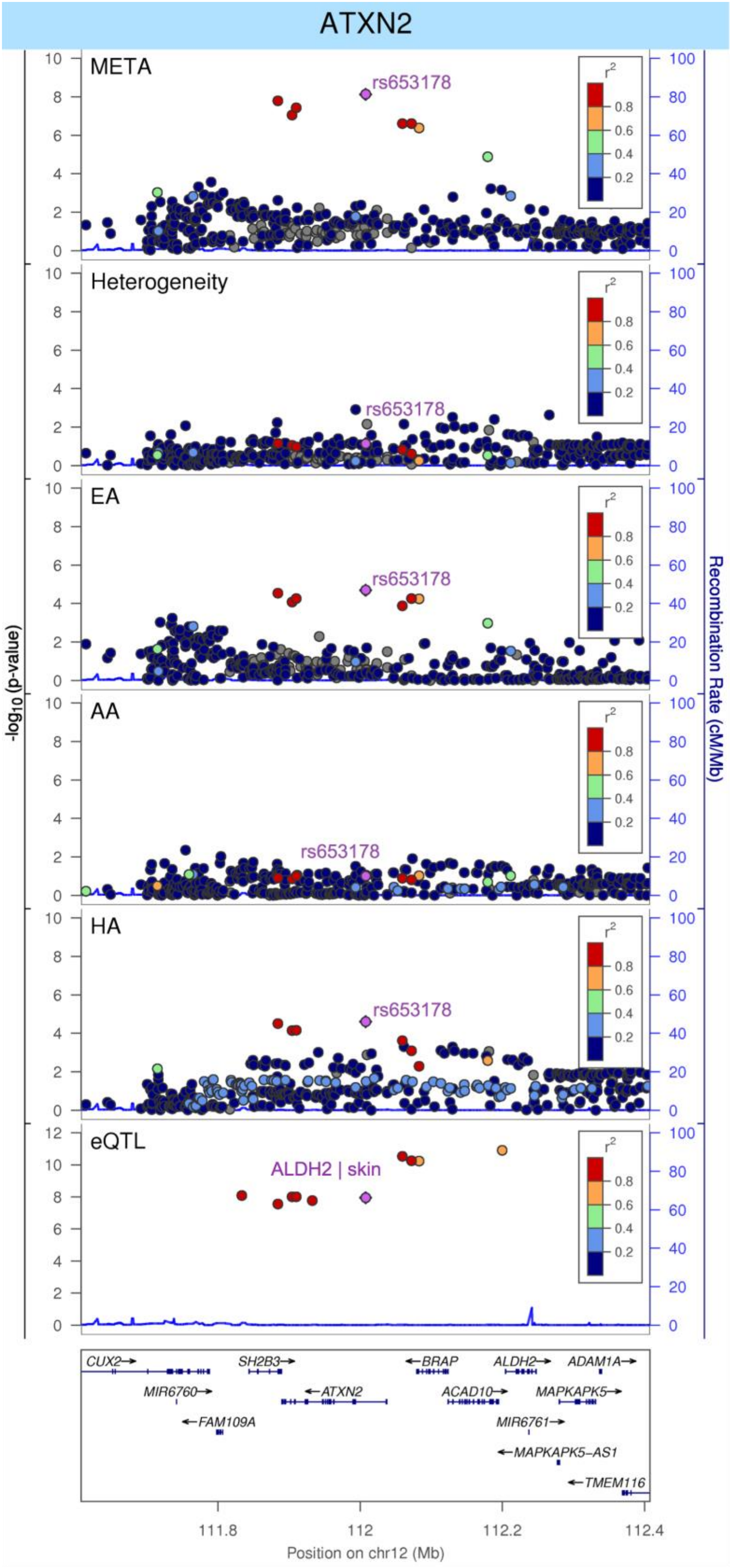
ATXN2
META
rs653178
Heterogeneity
rs653178
EA
rs653178
AA
rs653178
HA
rs653178
eQTL
ALDH2 | skin
-log10 (p-value)
Recombination Rate (cM/Mb)
CUX2
SH2B3
BRAP
ALDH2
ADAM1A
MIR6760
ATXN2
ACAD10
MAPKAPK5
FAM109A
MIR6761
MAPKAPK5-AS1
TMEM116
111.8
112
112.2
112.4
Position on chr12 (Mb)

j. CCL22

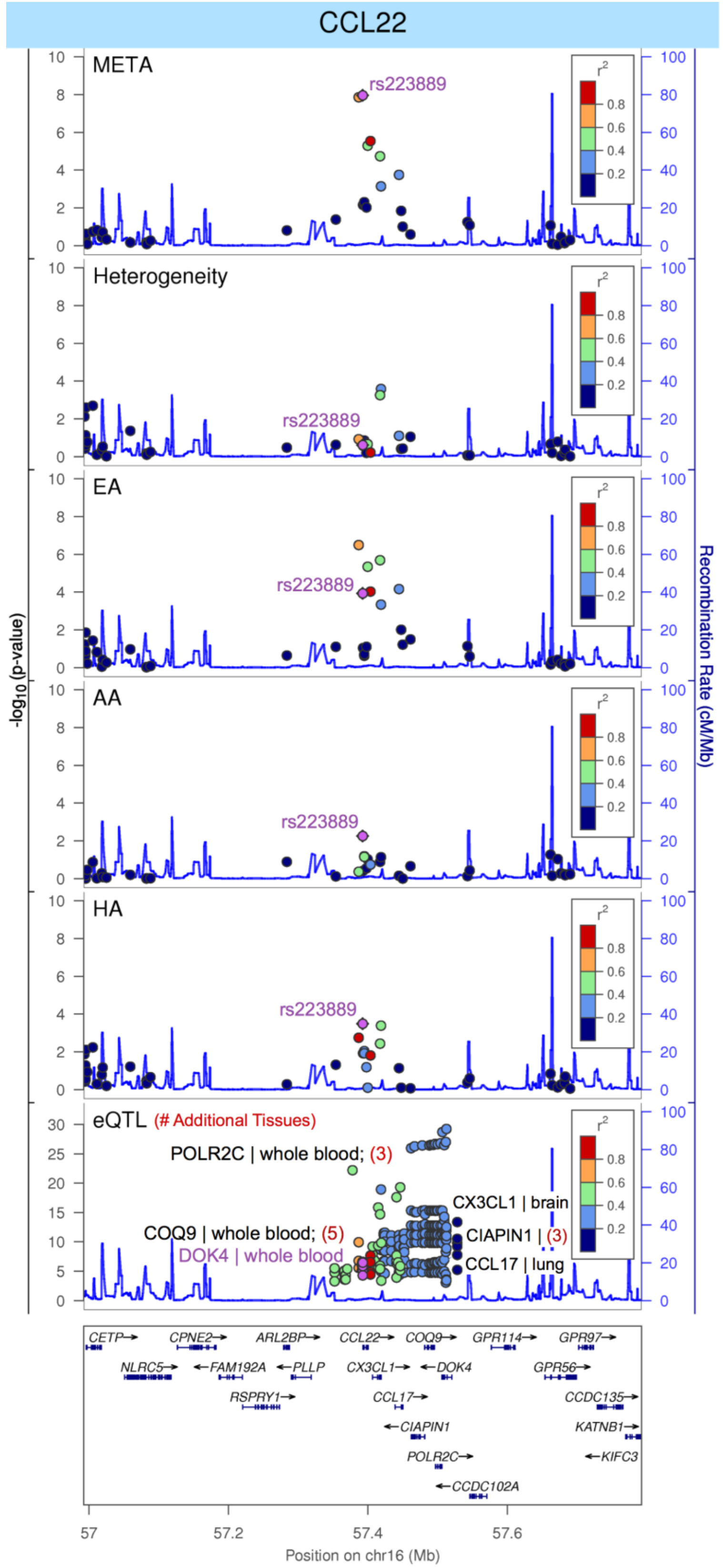

CCL22
META
rs223889
Heterogeneity
EA
AA
HA
eQTL (# Additional Tissues)
POLR2C | whole blood; (3)
CX3CL1 | brain
COQ9 | whole blood; (5)
CIAPIN1 | (3)
DOK4 | whole blood
CCL17 | lung
-log10 (p-value)
Recombination Rate (cM/Mb)
r2
CETP
CPNE2
ARL2BP
CCL22
COQ9
GPR114
GPR97
NLRC5
FAM192A
PLLP
CX3CL1
DOK4
GPR56
RSPRY1
CCL17
CCDC135
CIAPIN1
KATNB1
POLR2C
KIFC3
CCDC102A
Position on chr16 (Mb)

k. CLEC16A

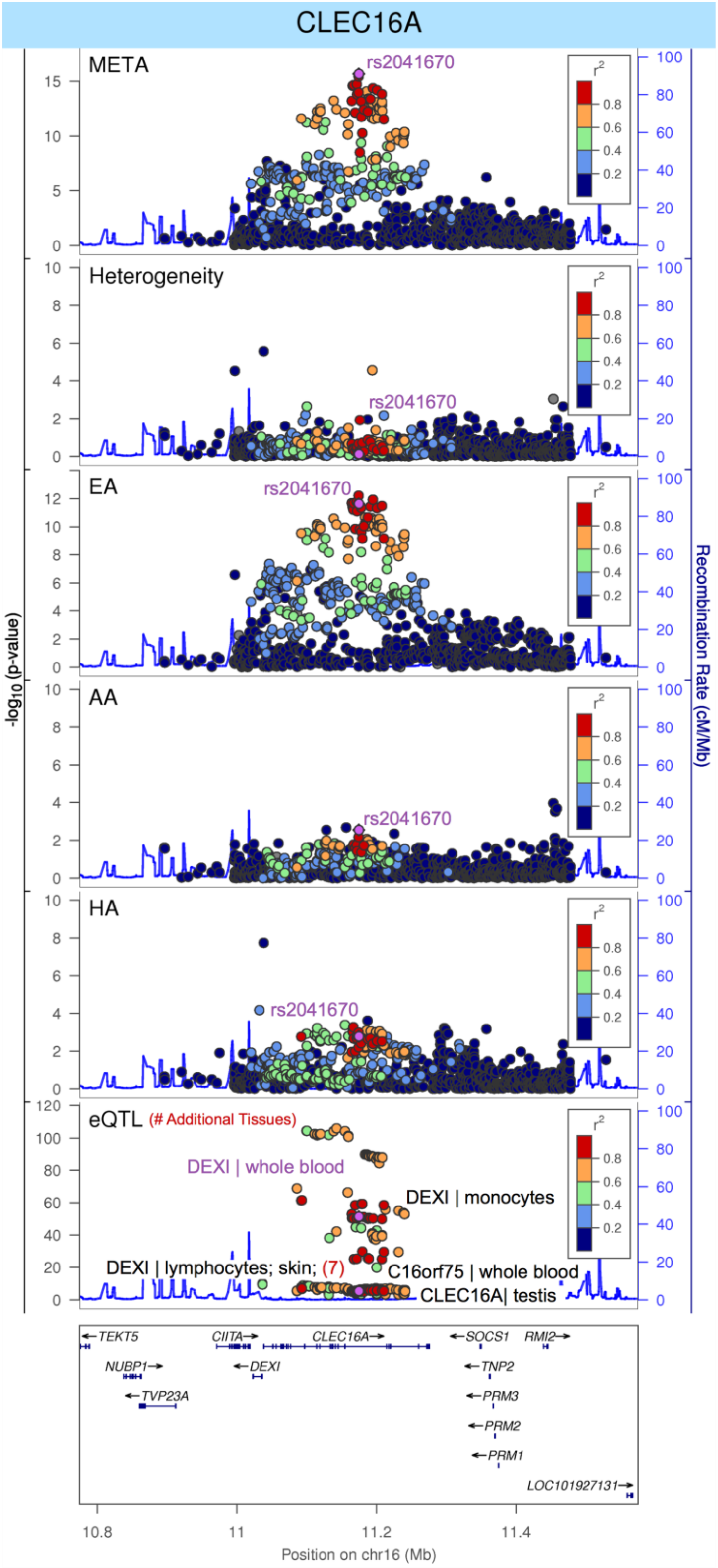

CLEC16A
META
rs2041670
Heterogeneity
rs2041670
EA
rs2041670
AA
rs2041670
HA
rs2041670
eQTL (# Additional Tissues)
DEXI | whole blood
DEXI | monocytes
DEXI | lymphocytes; skin; (7)
C16orf75 | whole blood
CLEC16A| testis
-log10 (p-value)
Recombination Rate (cM/Mb)
r2
TEKT5
CIITA
CLEC16A
SOCS1
RMI2
NUBP1
DEXI
TNP2
TVP23A
PRM3
PRM2
PRM1
LOC101927131
Position on chr16 (Mb)

I. ZFP90

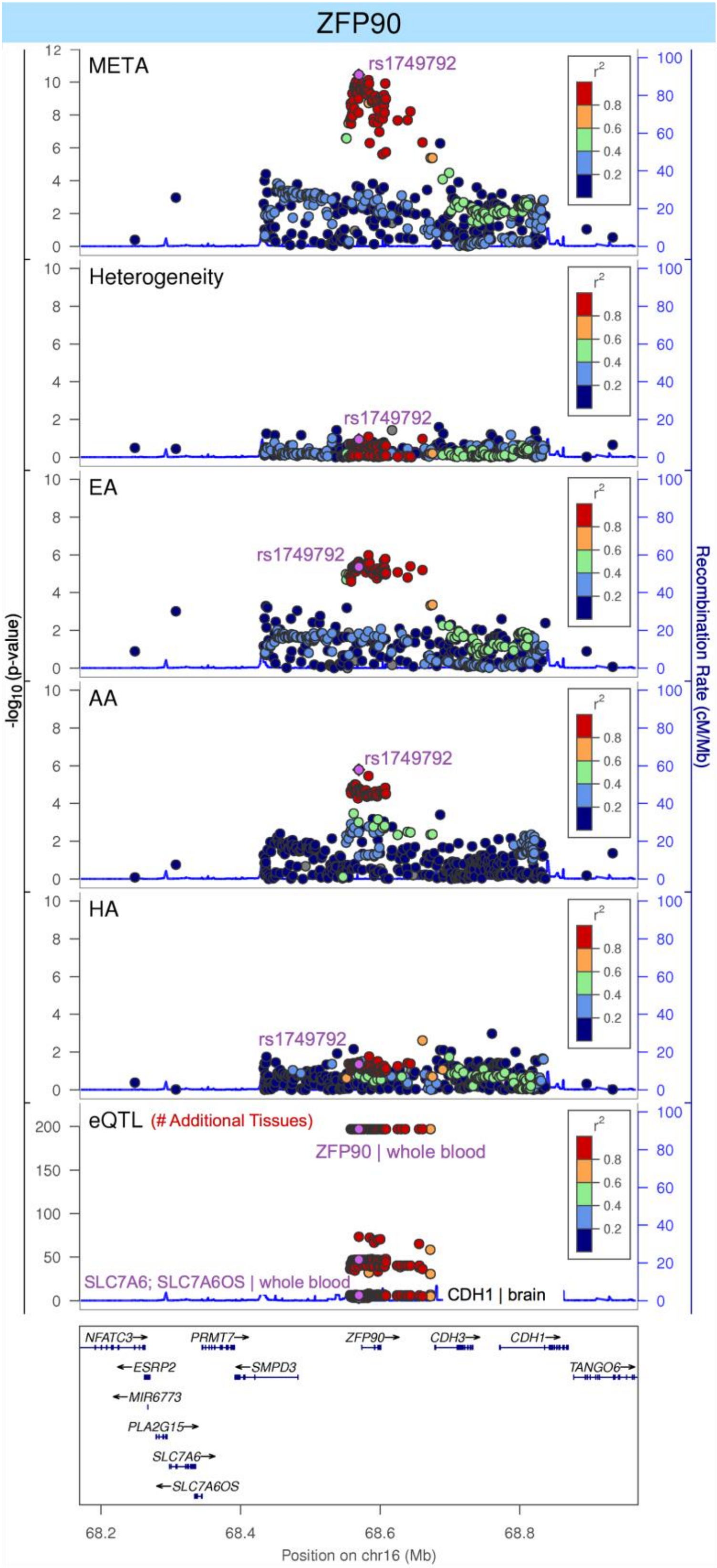

ZFP90
META
rs1749792
Heterogeneity
rs1749792
EA
rs1749792
AA
rs1749792
HA
rs1749792
eQTL (# Additional Tissues)
ZFP90 | whole blood
SLC7A6; SLC7A6OS | whole blood
CDH1 | brain
-log10 (p-value)
Recombination Rate (cM/Mb)
r2
NFATC3 PRMT7 ZFP90 CDH3 CDH1
ESRP2 SMPD3 TANGO6
MIR6773
PLA2G15
SLC7A6
SLC7A6OS
68.2 68.4 68.6 68.8
Position on chr16 (Mb)

m. GRB2

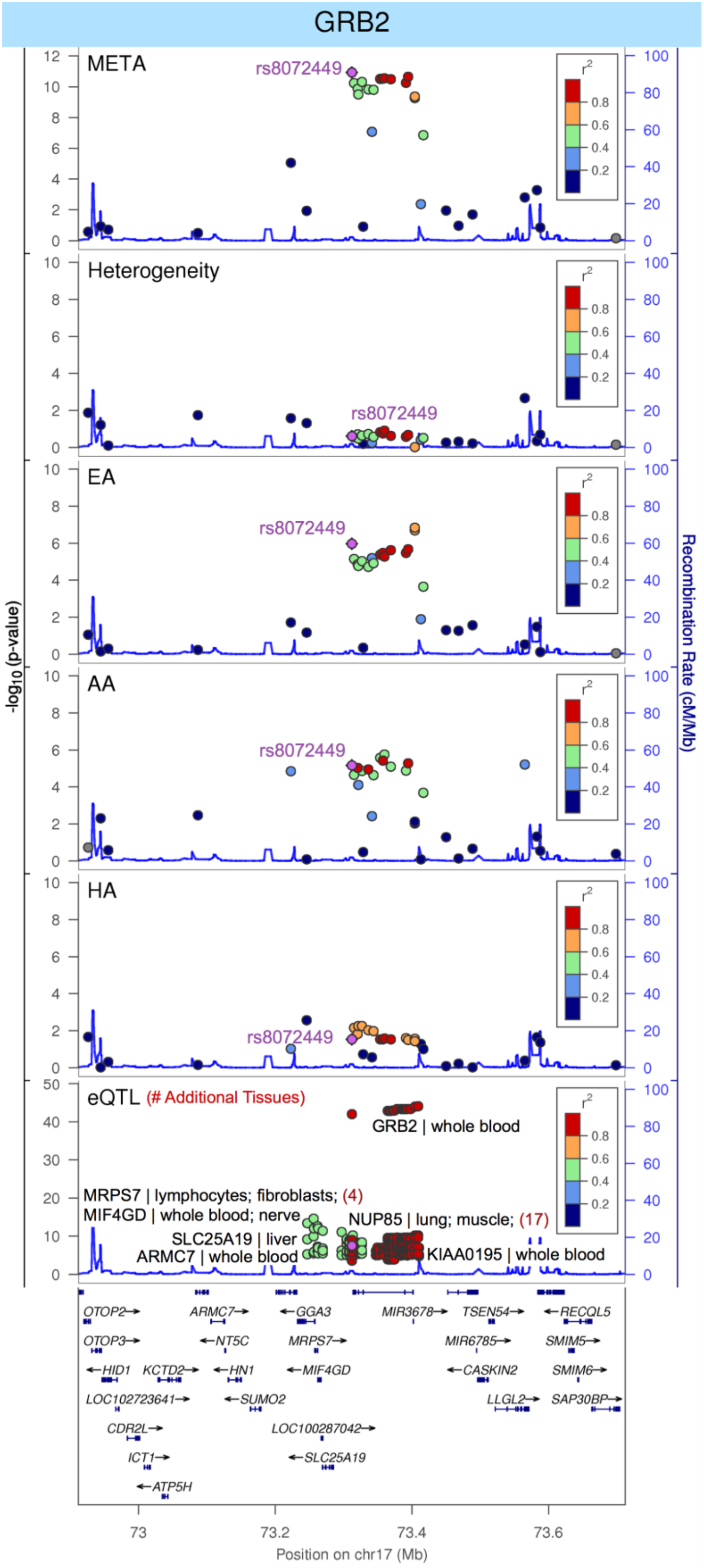
GRB2
META
rs8072449
Heterogeneity
rs8072449
EA
rs8072449
AA
rs8072449
HA
rs8072449
eQTL (# Additional Tissues)
GRB2 | whole blood
MRPS7 | lymphocytes; fibroblasts; (4)
MIF4GD | whole blood; nerve
NUP85 | lung; muscle; (17)
SLC25A19 | liver
ARMC7 | whole blood
KIAA0195 | whole blood
-log10 (p-value)
Recombination Rate (cM/Mb)
r2
OTOP2
OTOP3
HID1
KCTD2
LOC102723641
CDR2L
ICT1
ATP5H
ARMC7
NT5C
HN1
SUMO2
GGA3
MRPS7
MIF4GD
LOC100287042
SLC25A19
MIR3678
TSEN54
MIR6785
CASKIN2
LLGL2
RECQL5
SMIM5
SMIM6
SAP30BP
Position on chr17 (Mb)

n. LRRC25-SSPB4

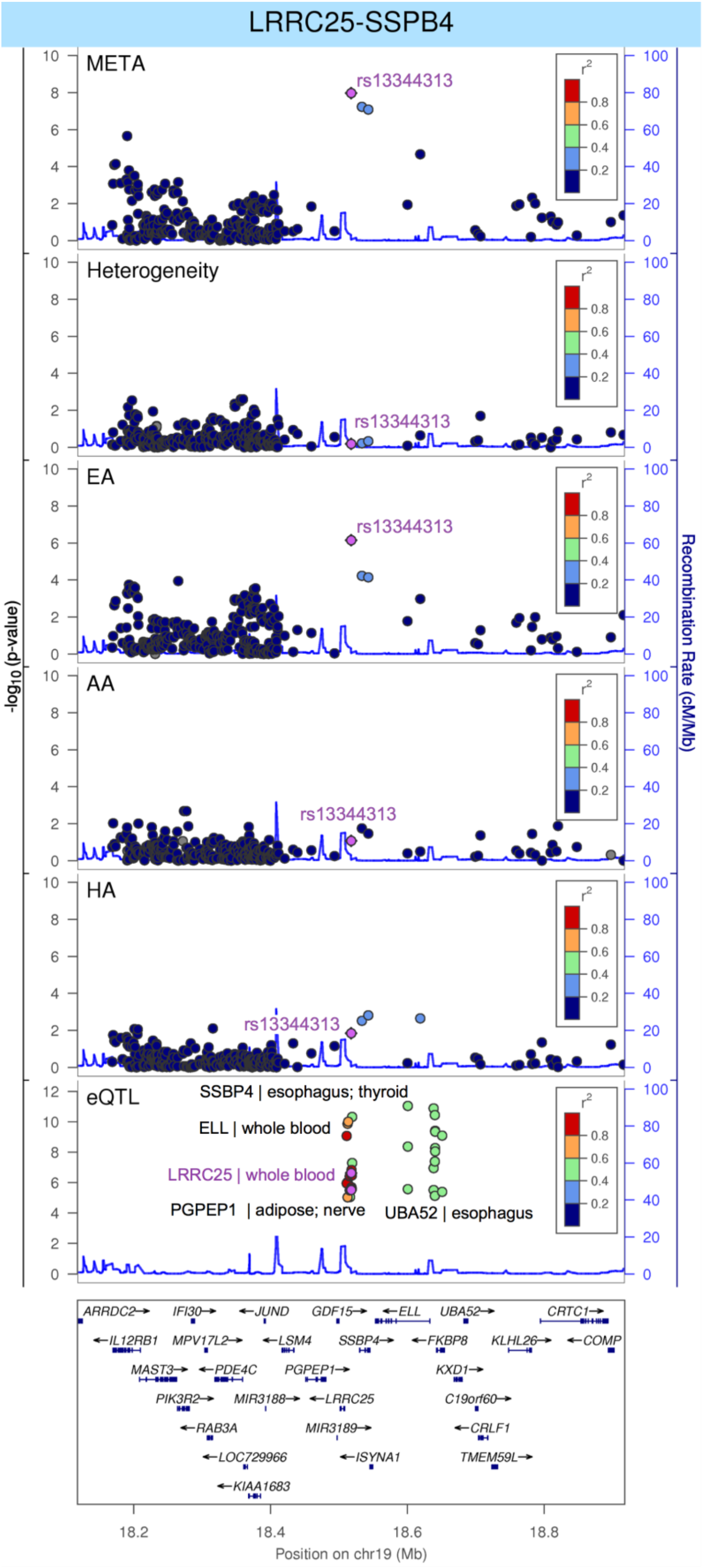

o. PTPRH-TMEM86B

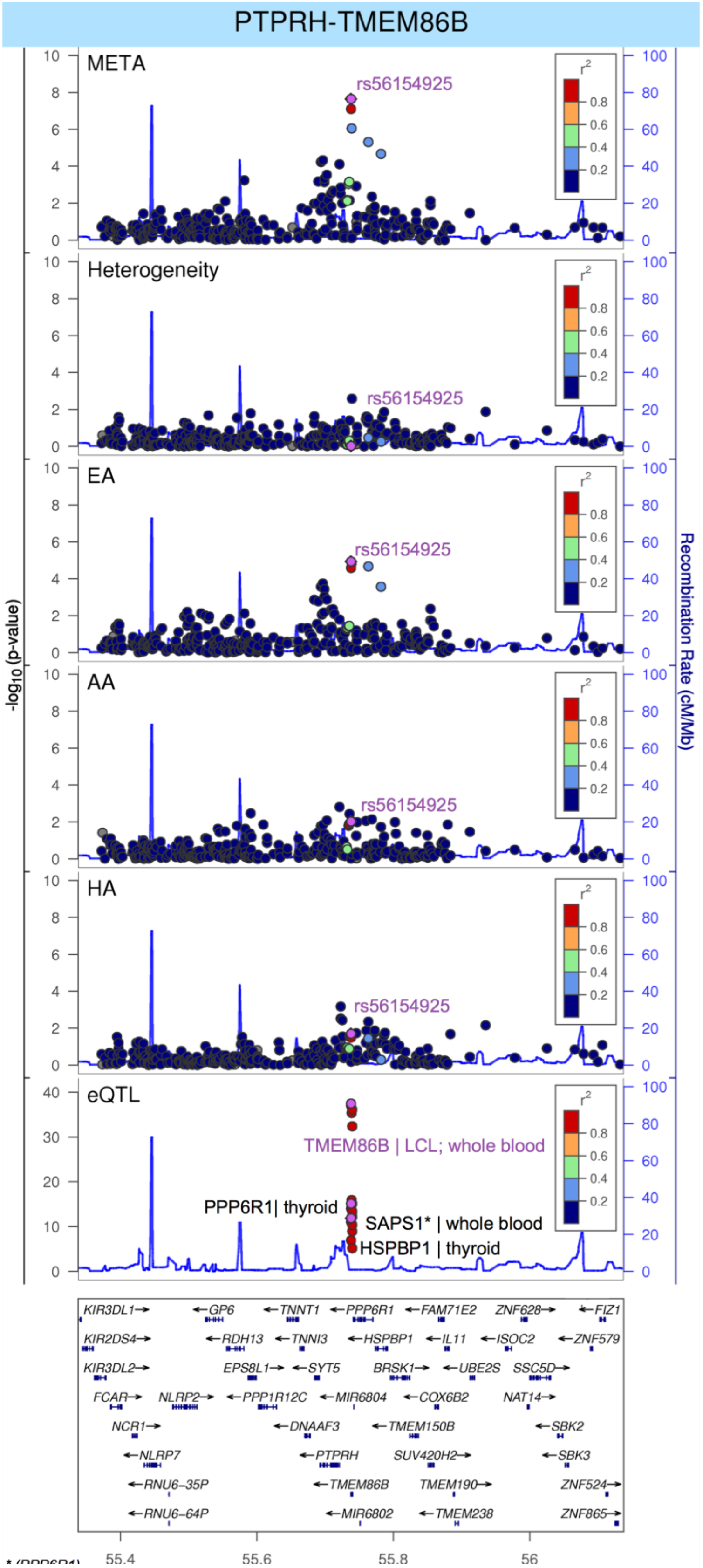

p. ENTHD1-GRAP2

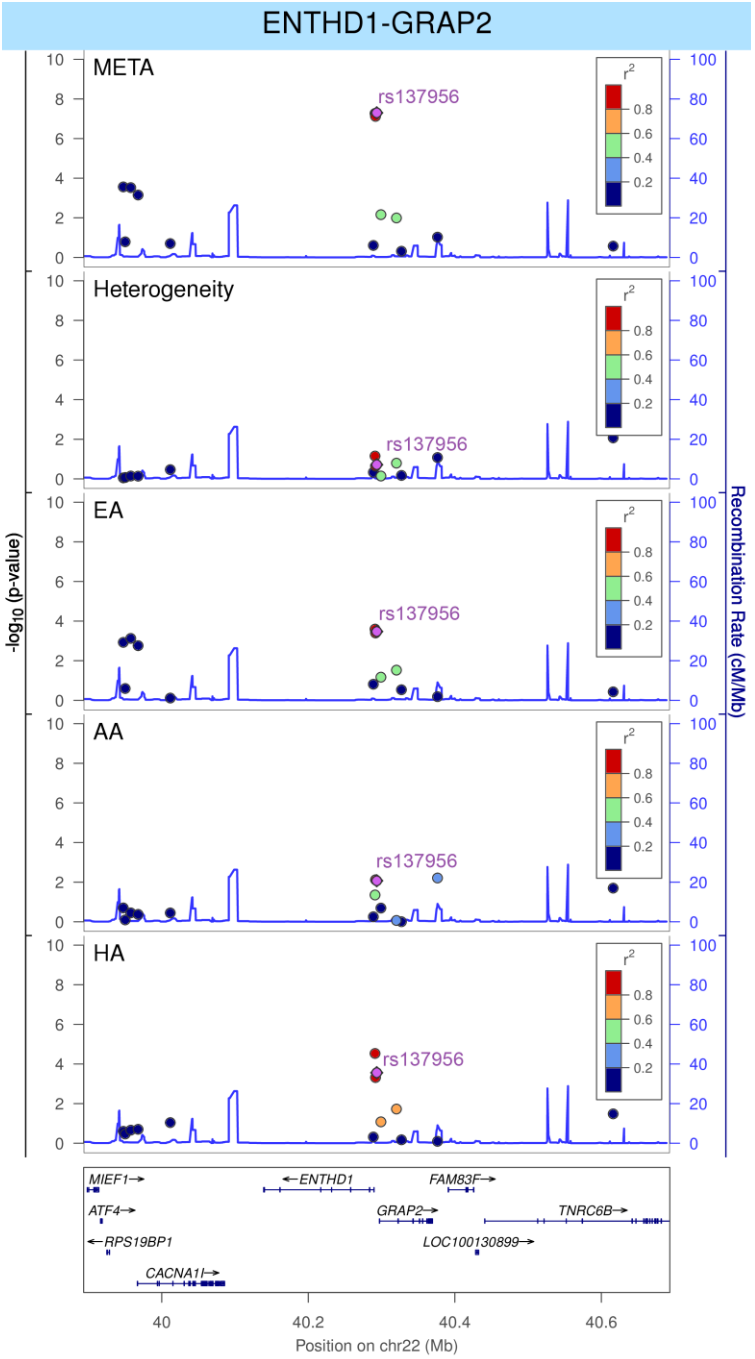

Supplementary Figure 14. Plots of novel Tier 2 meta-analysis and transancestral-mapping regions. Plots appear in the order found in Table 3b. Individual plots were created using LocusZoom standalone software (*http://locuszoom.sph.umich.edu/*).

a. GFI1

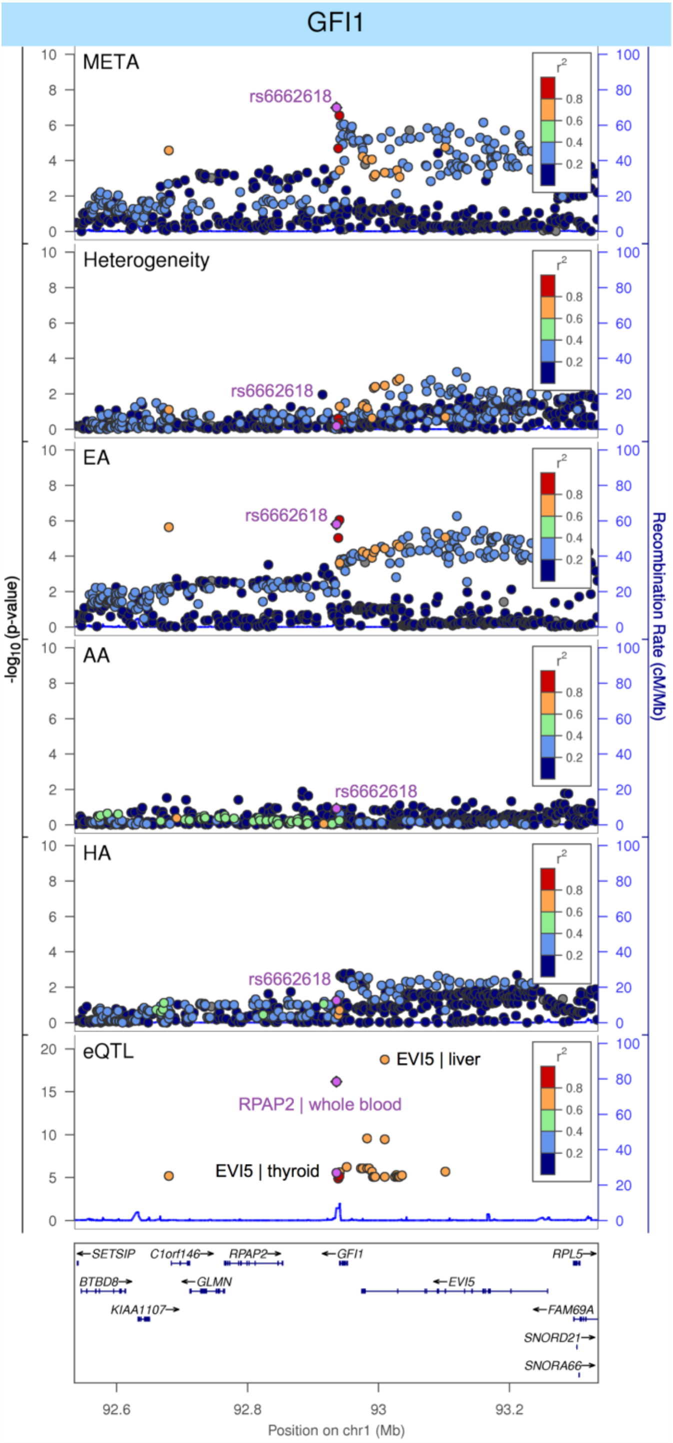

b. NOTCH2

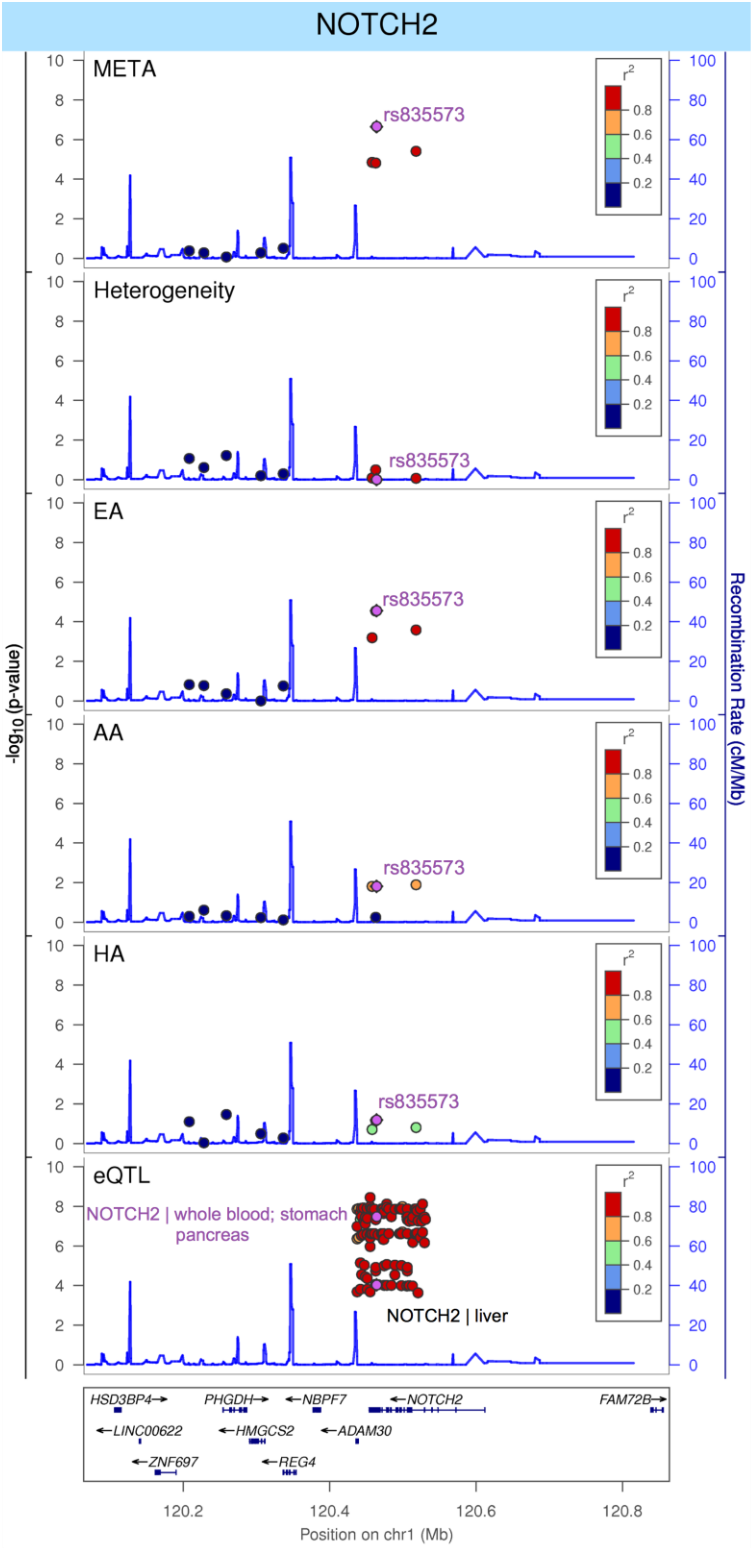
NOTCH2
META
Heterogeneity
EA
AA
HA
eQTL
rs835573
NOTCH2 | whole blood; stomach
pancreas
NOTCH2 | liver
r2
0.8
0.6
0.4
0.2
-log10 (p-value)
Recombination Rate (cM/Mb)
HSD3BP4
PHGDH
NBPF7
NOTCH2
FAM72B
LINC00622
HMGCS2
ADAM30
ZNF697
REG4
120.2
120.4
120.6
120.8
Position on chr1 (Mb)

c. FASLG

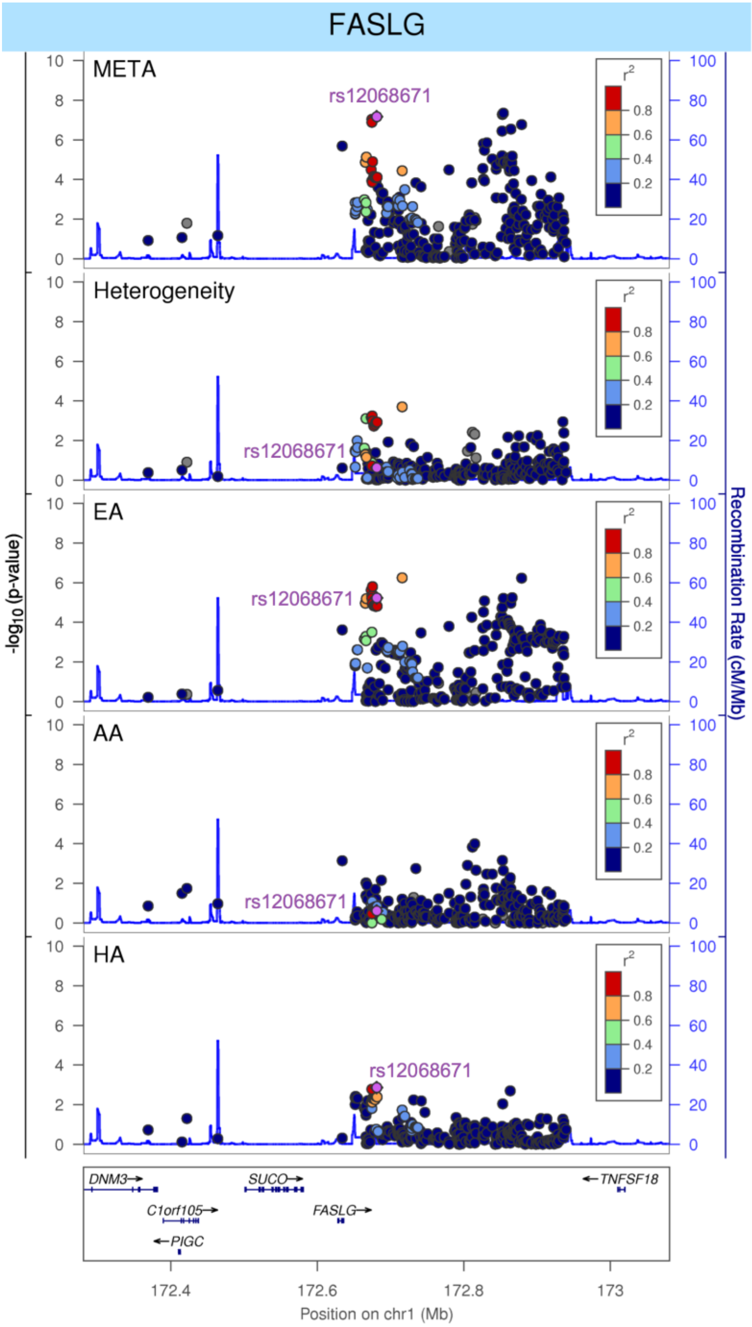

FASLG
META
Heterogeneity
EA
AA
HA
rs12068671
-log10 (p-value)
Recombination Rate (cM/Mb)
r2
0.8
0.6
0.4
0.2
DNM3
SUCO
TNFSF18
C1orf105
FASLG
PIGC
172.4
172.6
172.8
173
Position on chr1 (Mb)

d. LBH

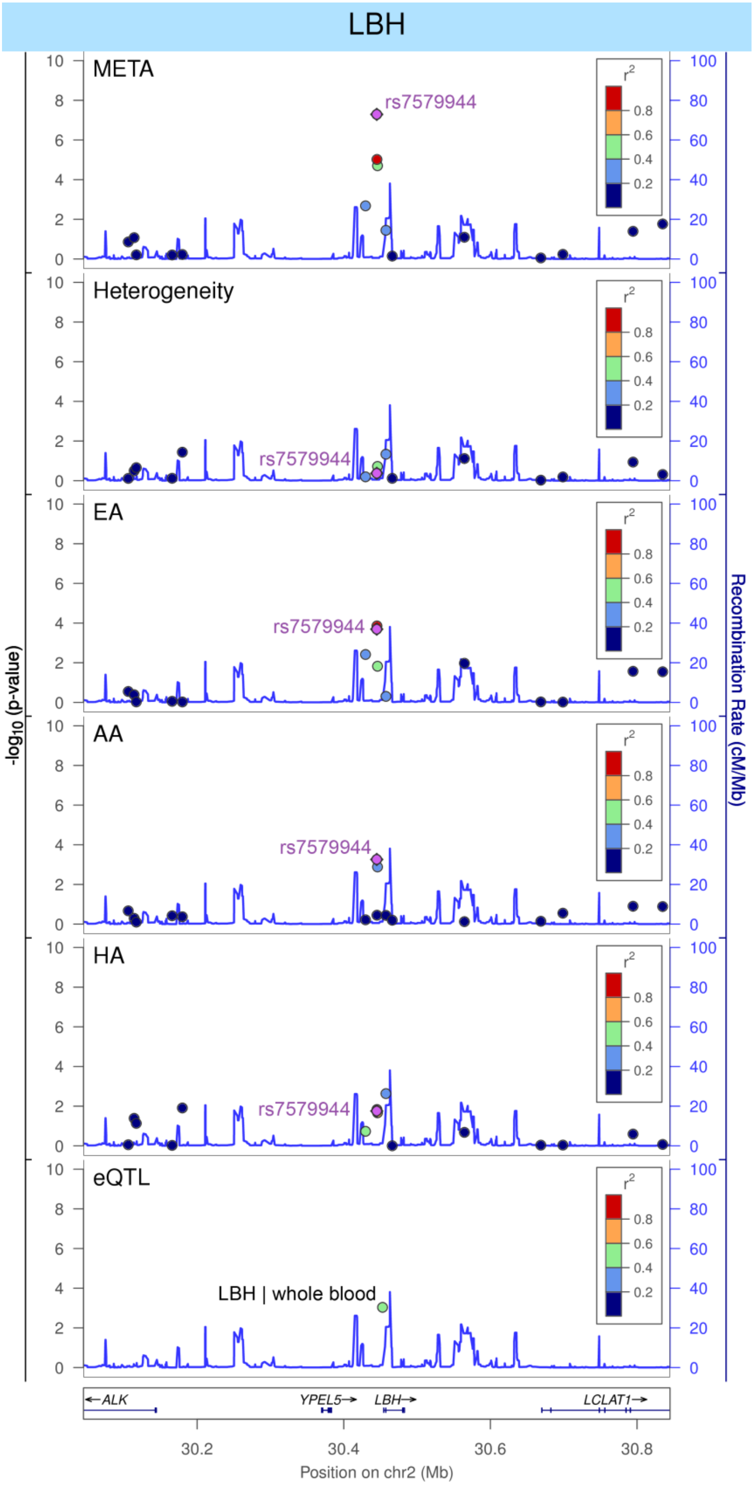
LBH
META
rs7579944
Heterogeneity
rs7579944
EA
rs7579944
AA
rs7579944
HA
rs7579944
eQTL
LBH | whole blood
r2
0.8
0.6
0.4
0.2
-log10 (p-value)
Recombination Rate (cM/Mb)
ALK
YPEL5
LBH
LCLAT1
30.2
30.4
30.6
30.8
Position on chr2 (Mb)

e. CLPTM1L-SLC6A3

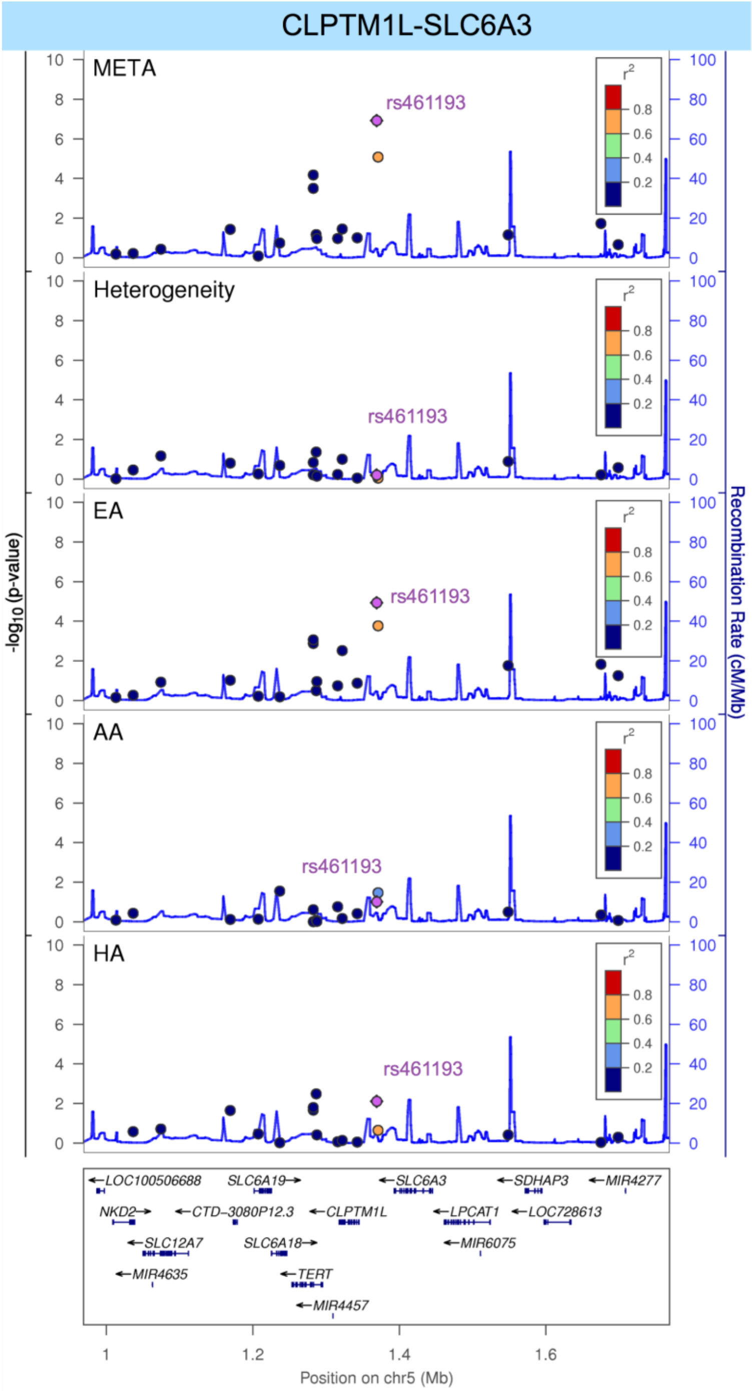

f. ATXN1

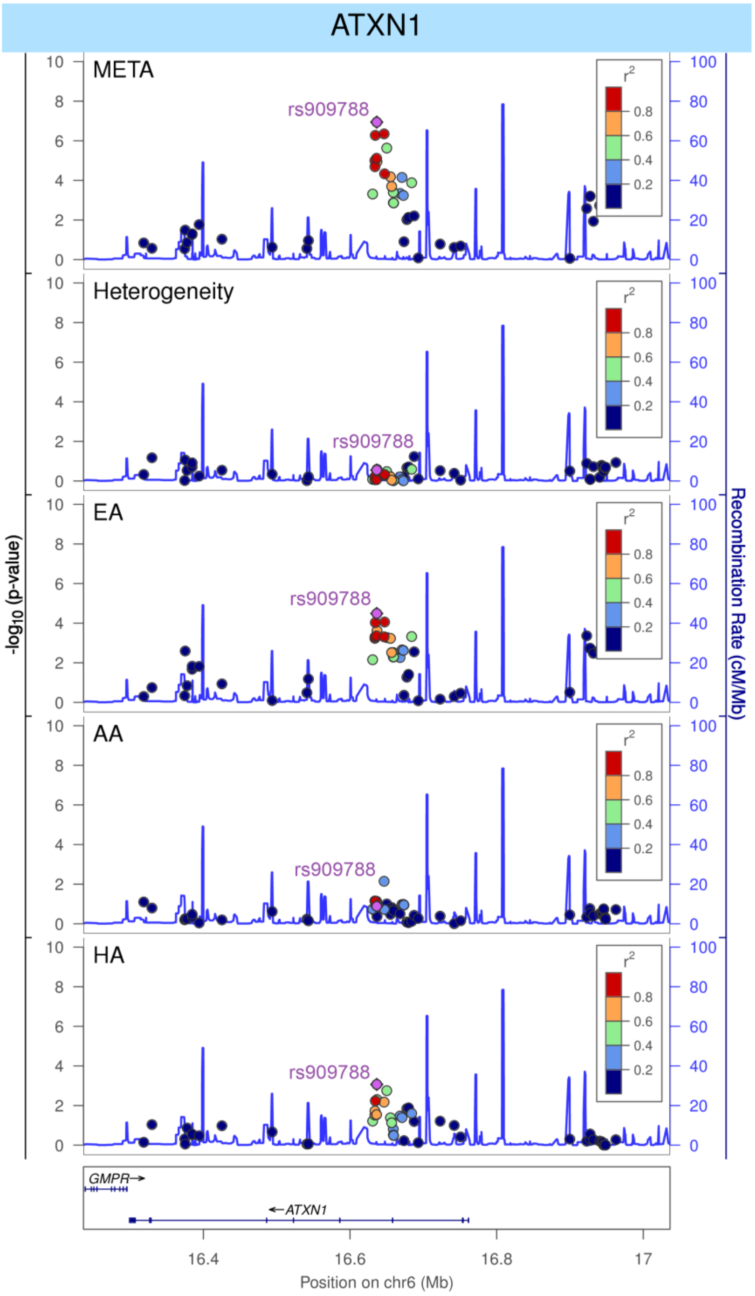
ATXN1
META
Heterogeneity
EA
AA
HA
rs909788
r2
0.8
0.6
0.4
0.2
-log10 (p-value)
Recombination Rate (cM/Mb)
GMPR
ATXN1
16.4
16.6
16.8
17
Position on chr6 (Mb)

g. AHI1

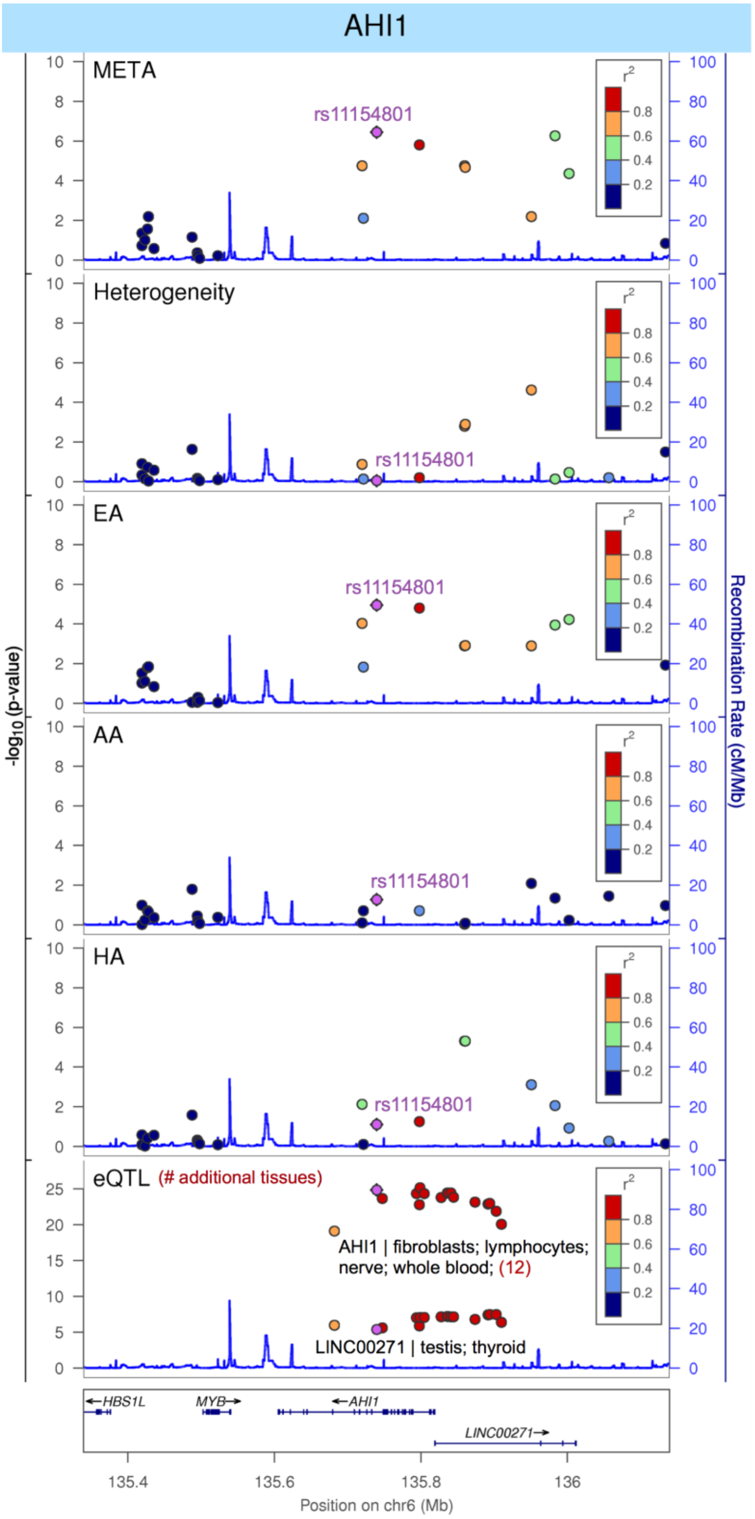

AHI1
META
Heterogeneity
EA
AA
HA
eQTL (# additional tissues)
rs11154801
AHI1 | fibroblasts; lymphocytes; nerve; whole blood; (12)
LINC00271 | testis; thyroid
-log10 (p-value)
Recombination Rate (cM/Mb)
r2
HBS1L
MYB
AHI1
LINC00271
135.4
135.6
135.8
136
Position on chr6 (Mb)

h. SKAP2

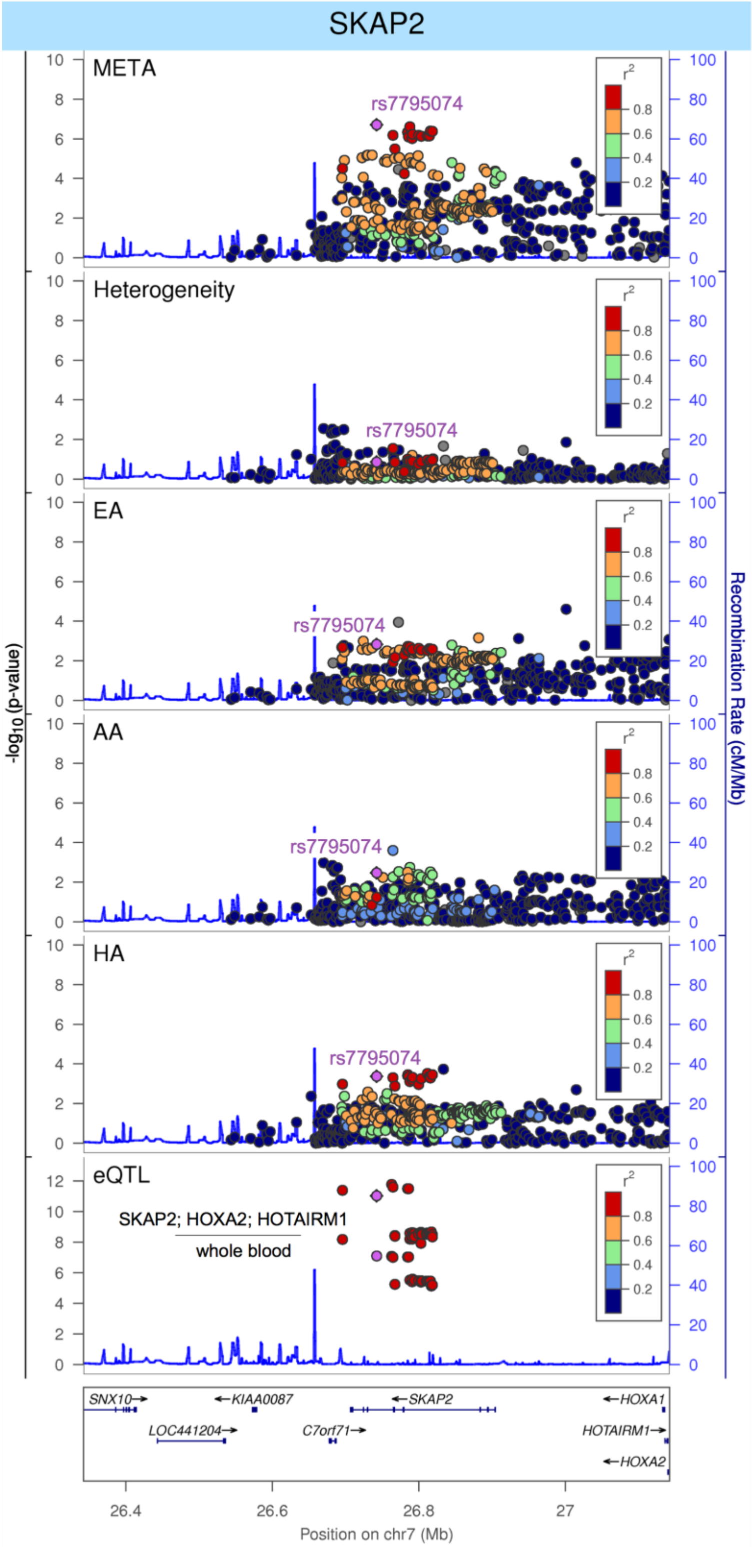

SKAP2
META
Heterogeneity
EA
AA
HA
eQTL
rs7795074
SKAP2; HOXA2; HOTAIRM1
whole blood
r^2
0.8
0.6
0.4
0.2
-log10 (p-value)
Recombination Rate (cM/Mb)
SNX10
KIAA0087
SKAP2
HOXA1
LOC441204
C7orf71
HOTAIRM1
HOXA2
26.4
26.6
26.8
27
Position on chr7 (Mb)

i. TNKS

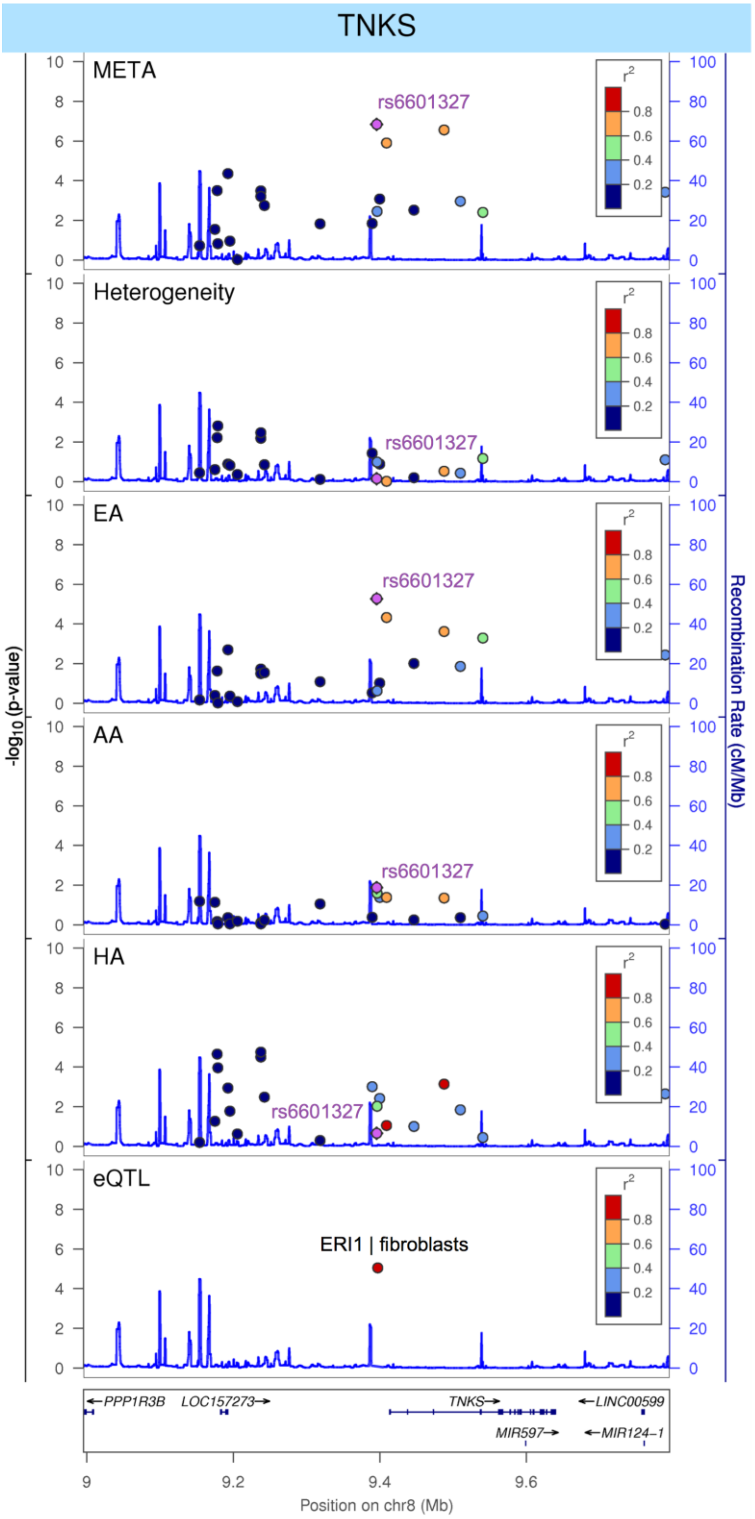
TNKS
META
rs6601327
Heterogeneity
rs6601327
EA
rs6601327
AA
rs6601327
HA
rs6601327
eQTL
ERI1 | fibroblasts
r2
0.8
0.6
0.4
0.2
-log10 (p-value)
Recombination Rate (cM/Mb)
PPP1R3B
LOC157273
TNKS
LINC00599
MIR597
MIR124-1
9
9.2
9.4
9.6
Position on chr8 (Mb)

j. TRAFD1-HECTD4

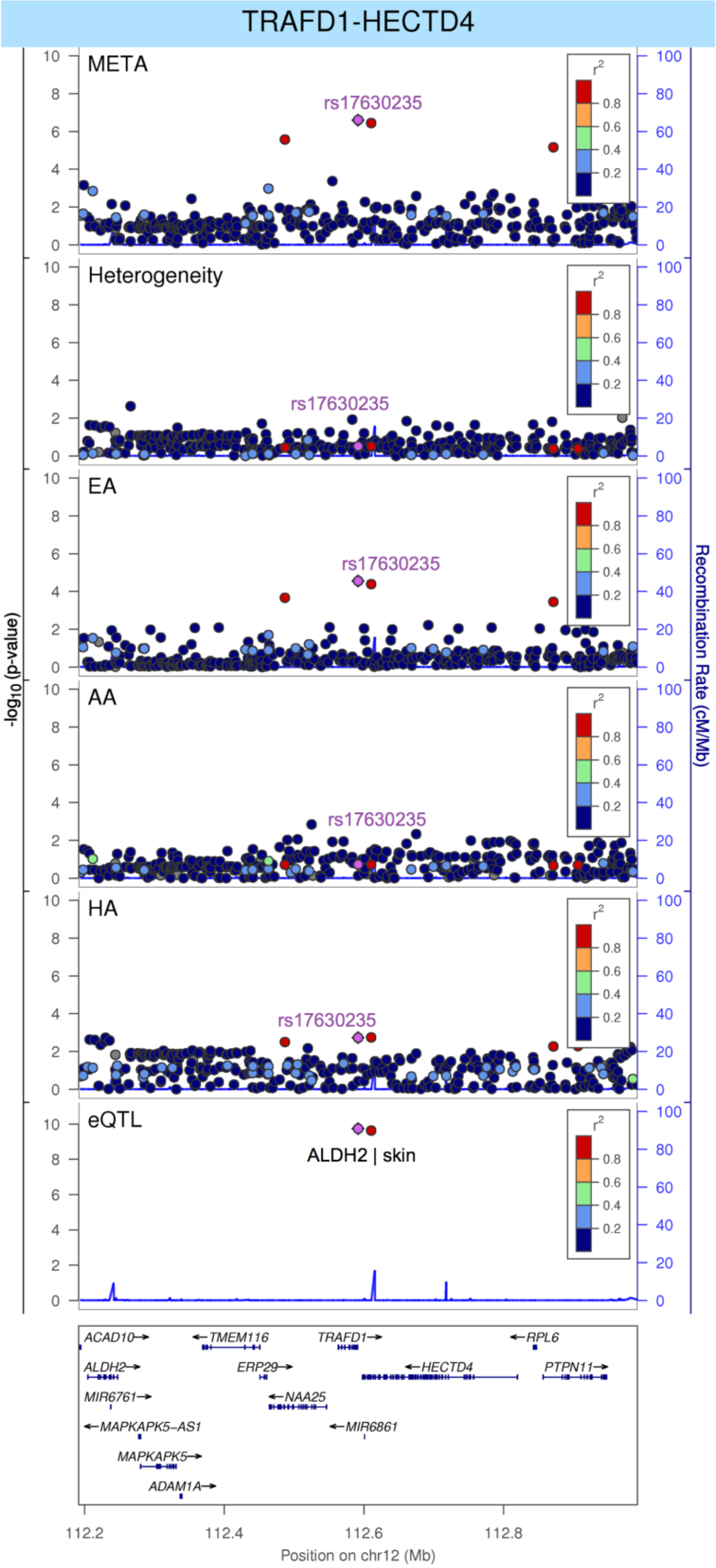

Supplementary Figure 15. Plots of Tier 1 meta-analysis regions noted for transancestral-mapping. Plots appear in the order found in Table 4. Individual plots were created using LocusZoom standalone software (*http://locuszoom.sph.umich.edu/*).

a. TNFSF4-LOC100506023

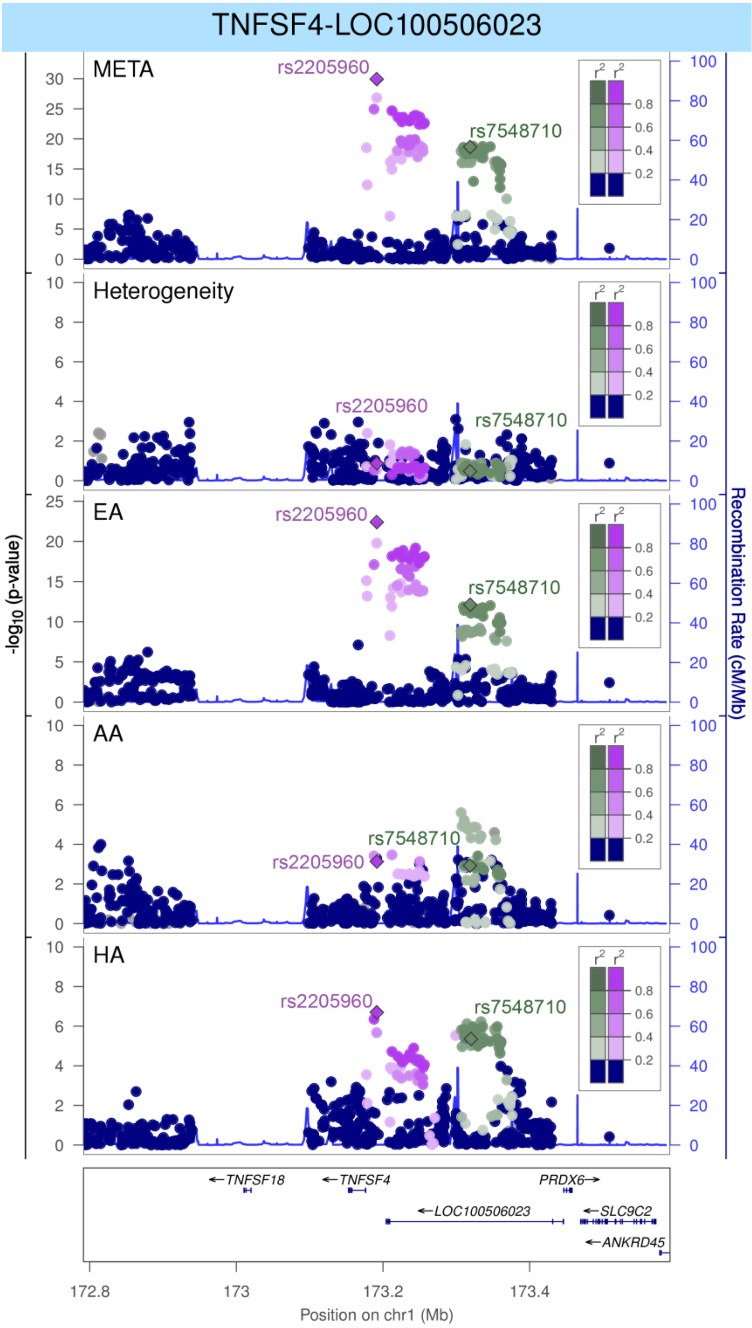

b. TNFSF4-LOC100506023

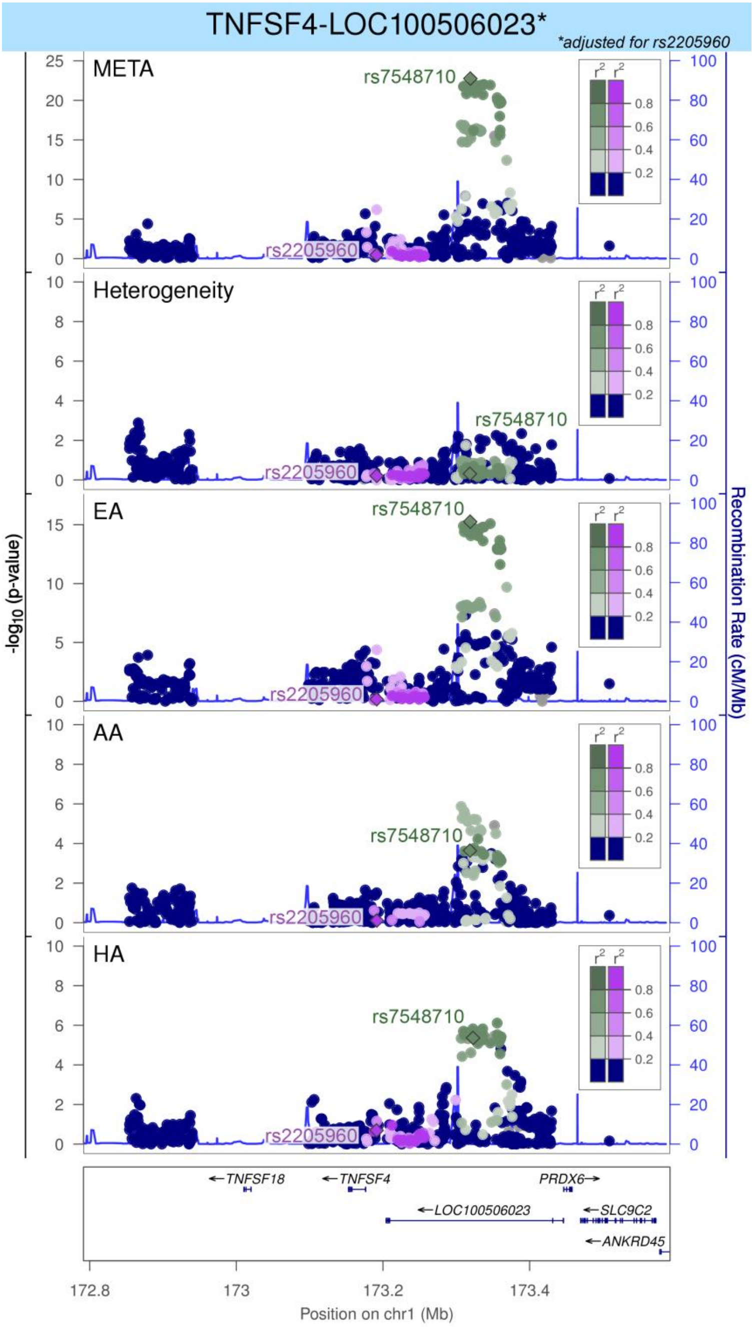

c. NMNAT2-SMG7-NCF2

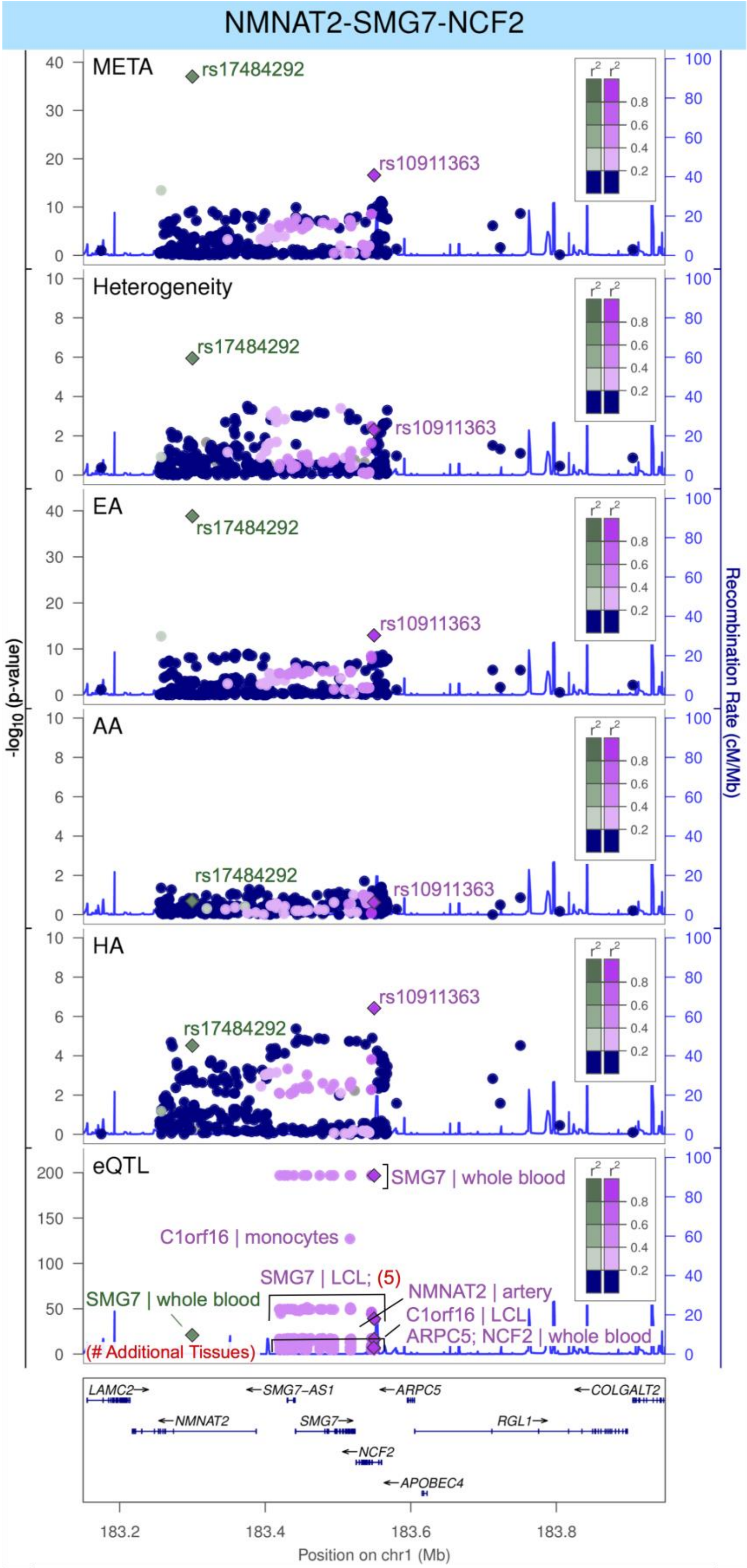

d. IL2-IL21

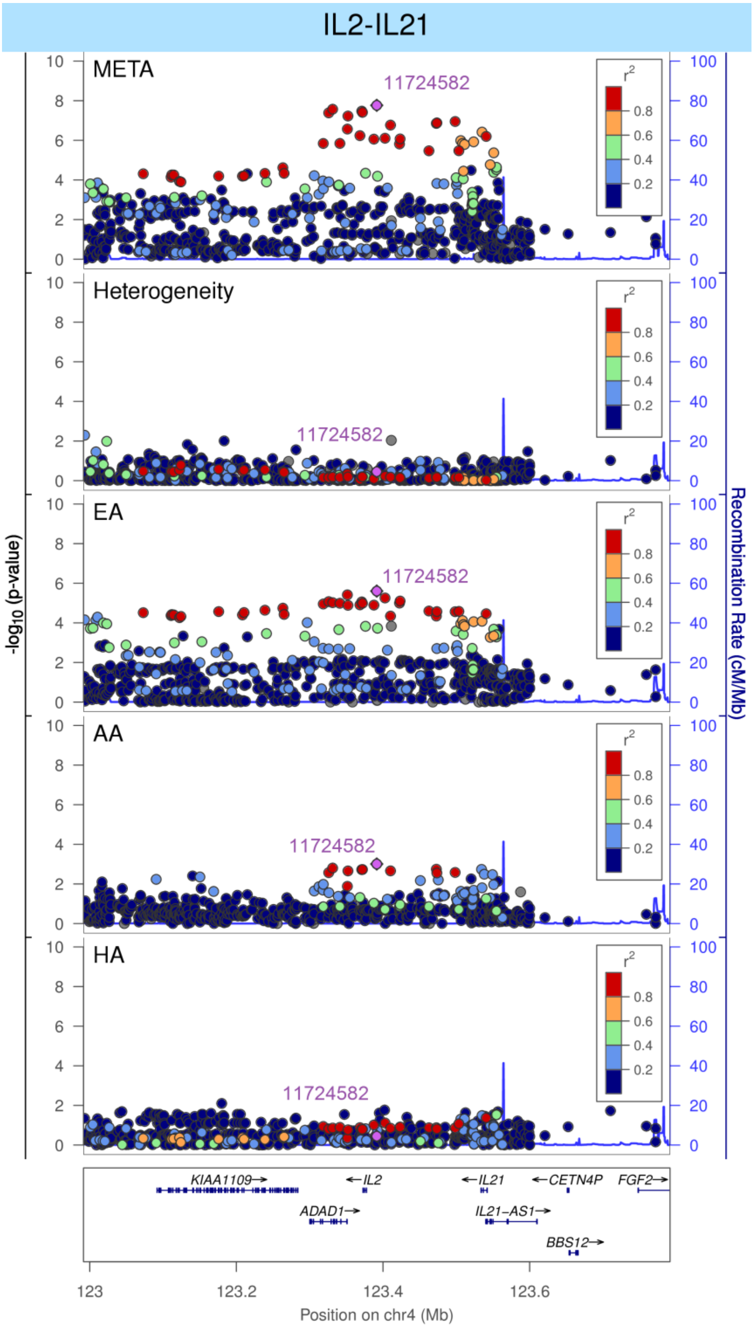
IL2-IL21
META
Heterogeneity
EA
AA
HA
11724582
-log10 (p-value)
Recombination Rate (cM/Mb)
r2
KIAA1109
IL2
IL21
CETN4P
FGF2
ADAD1
IL21−AS1
BBS12
Position on chr4 (Mb)

e. PTTG1-MIR146A

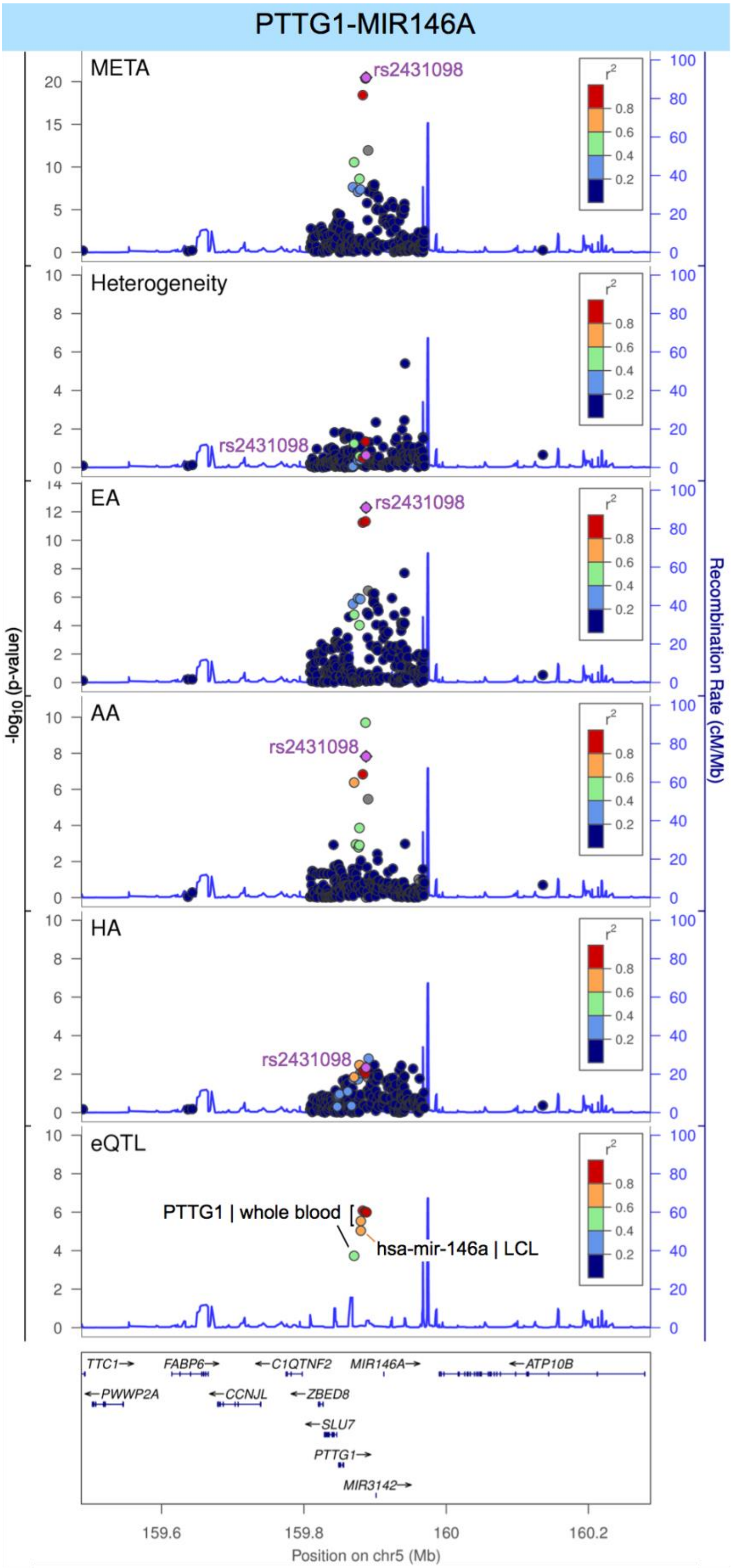

f. IRF5-TNPO3

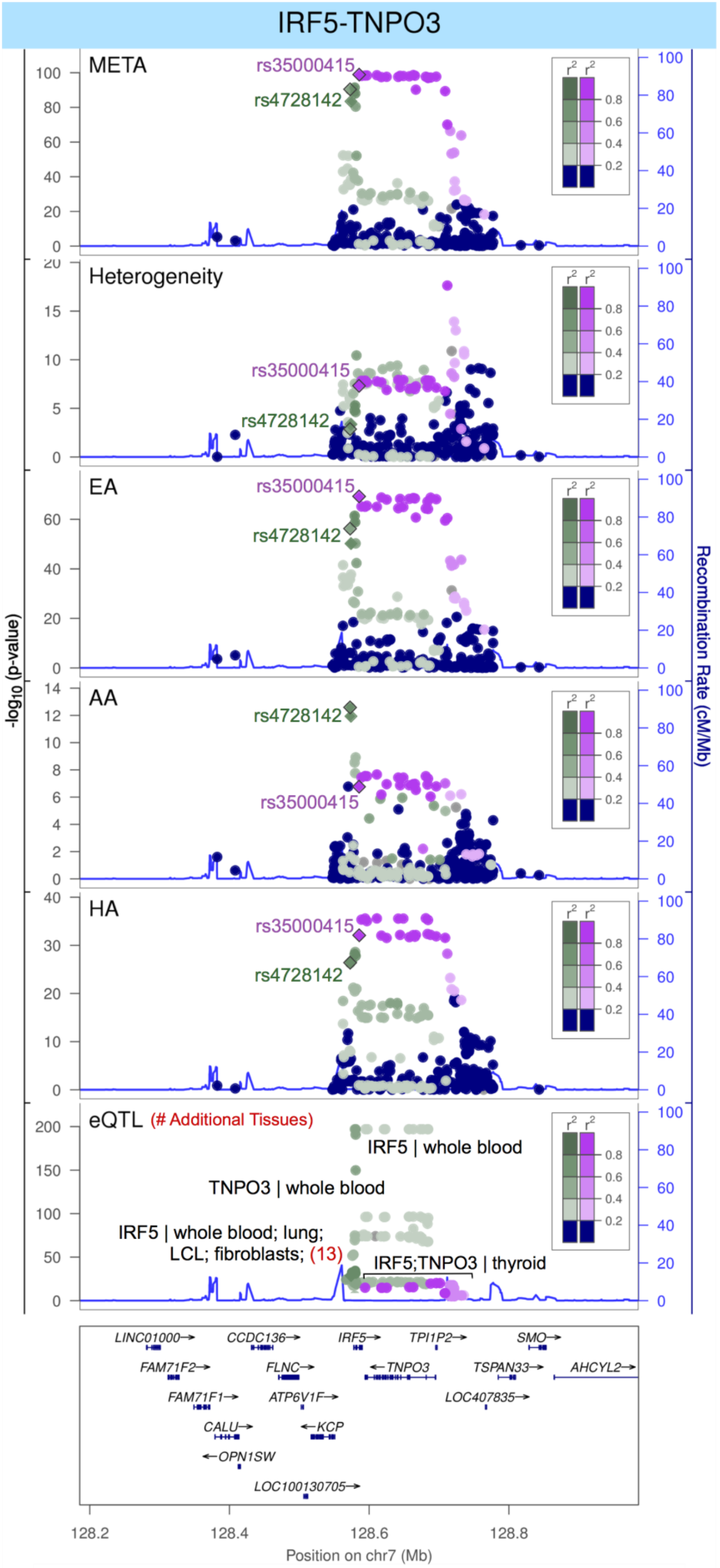

IRF5-TNPO3
META
Heterogeneity
EA
AA
HA
eQTL (# Additional Tissues)
rs35000415
rs4728142
-log10 (p-value)
Recombination Rate (cM/Mb)
IRF5 | whole blood
TNPO3 | whole blood
IRF5 | whole blood; lung;
LCL; fibroblasts; (13)
IRF5;TNPO3 | thyroid
LINC01000
CCDC136
IRF5
TPI1P2
SMO
FAM71F2
FLNC
TNPO3
TSPAN33
AHCYL2
FAM71F1
ATP6V1F
LOC407835
CALU
KCP
OPN1SW
LOC100130705
Position on chr7 (Mb)

g. IRF5-TNPO3 (adjusted for rs350004115)

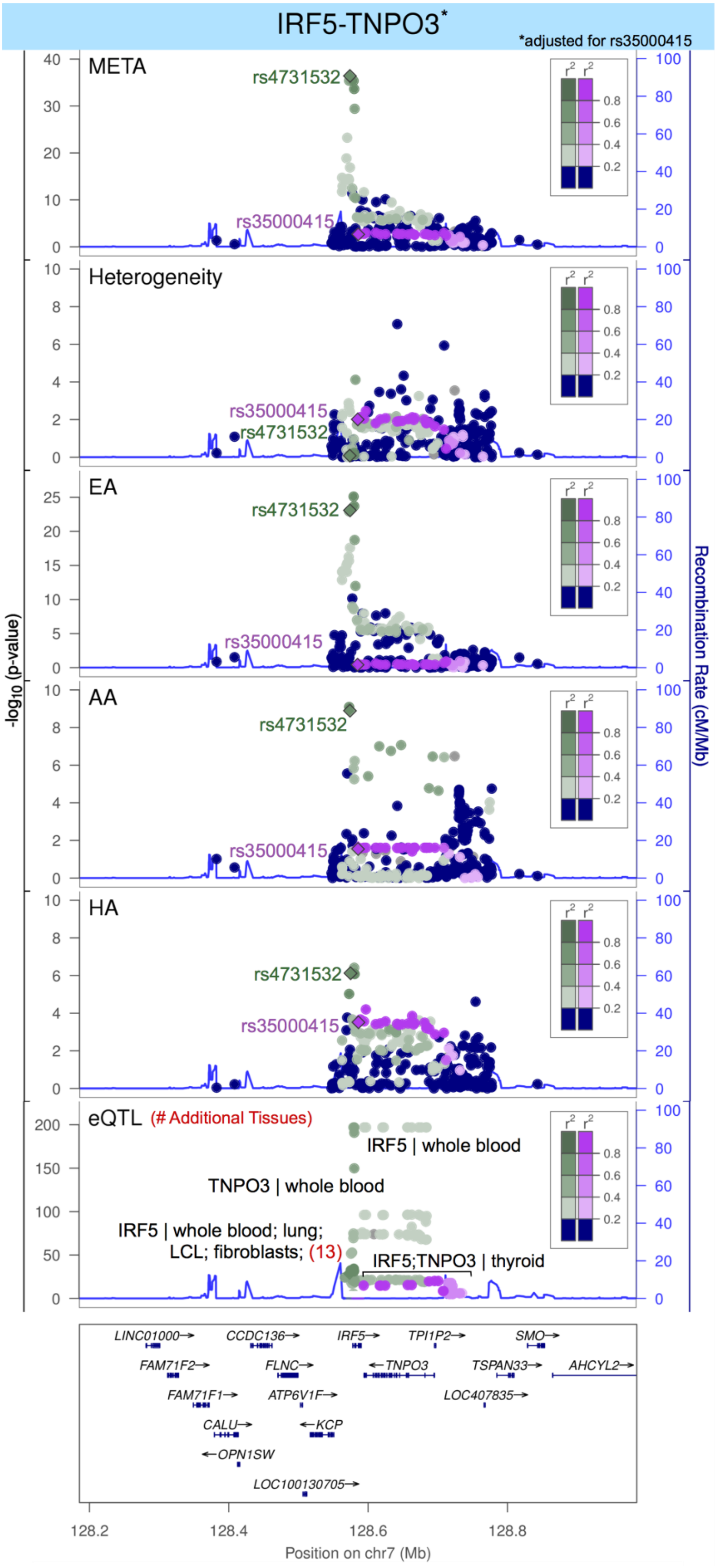

h. LYN-RPS20

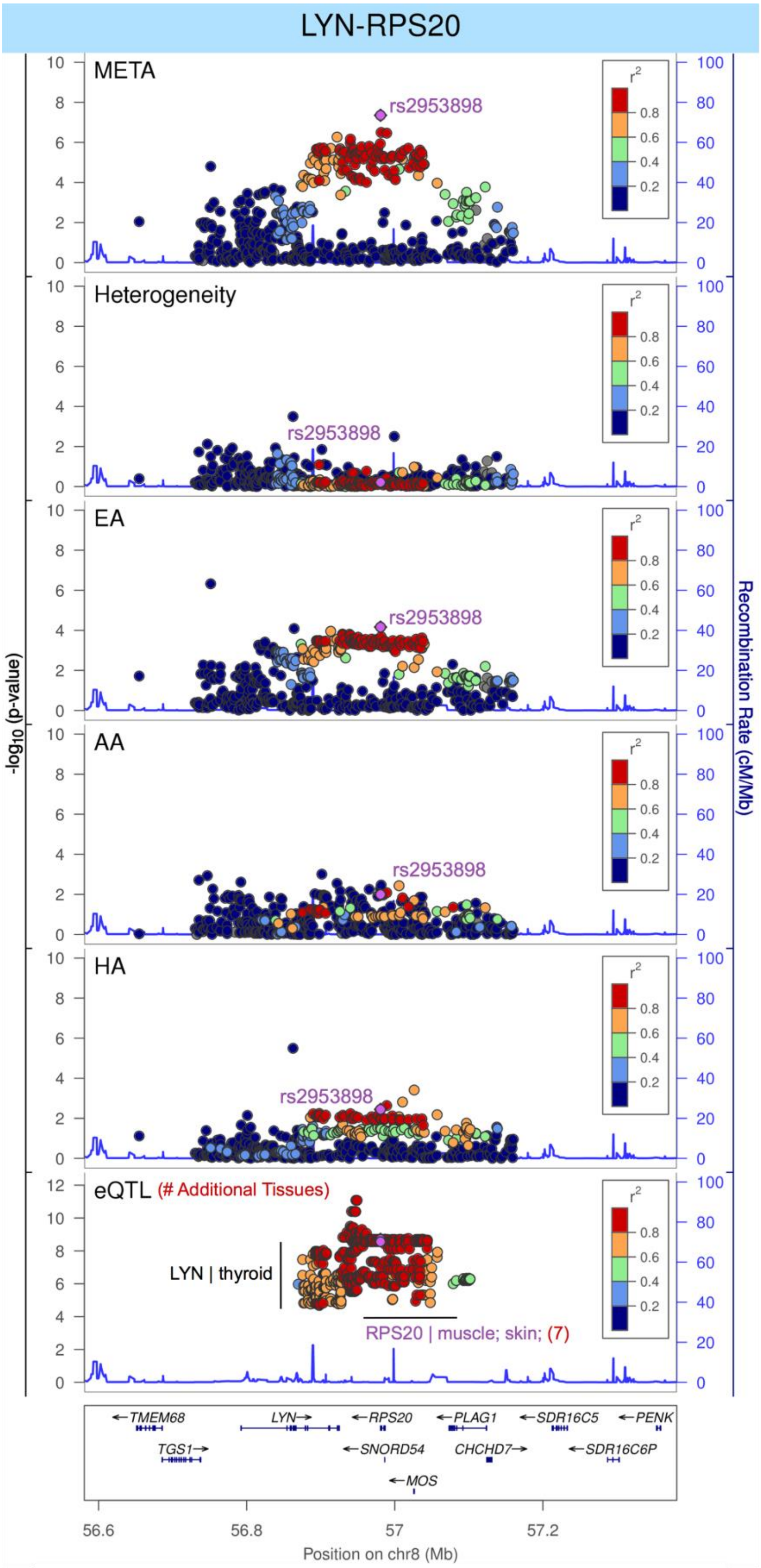

LYN-RPS20
META
rs2953898
Heterogeneity
EA
AA
HA
eQTL (# Additional Tissues)
LYN | thyroid
RPS20 | muscle; skin; (7)
r²
0.8
0.6
0.4
0.2
-log10 (p-value)
Recombination Rate (cM/Mb)
TMEM68
LYN
RPS20
PLAG1
SDR16C5
PENK
TGS1
SNORD54
CHCHD7
SDR16C6P
MOS
Position on chr8 (Mb)

i. PDHX-CD44

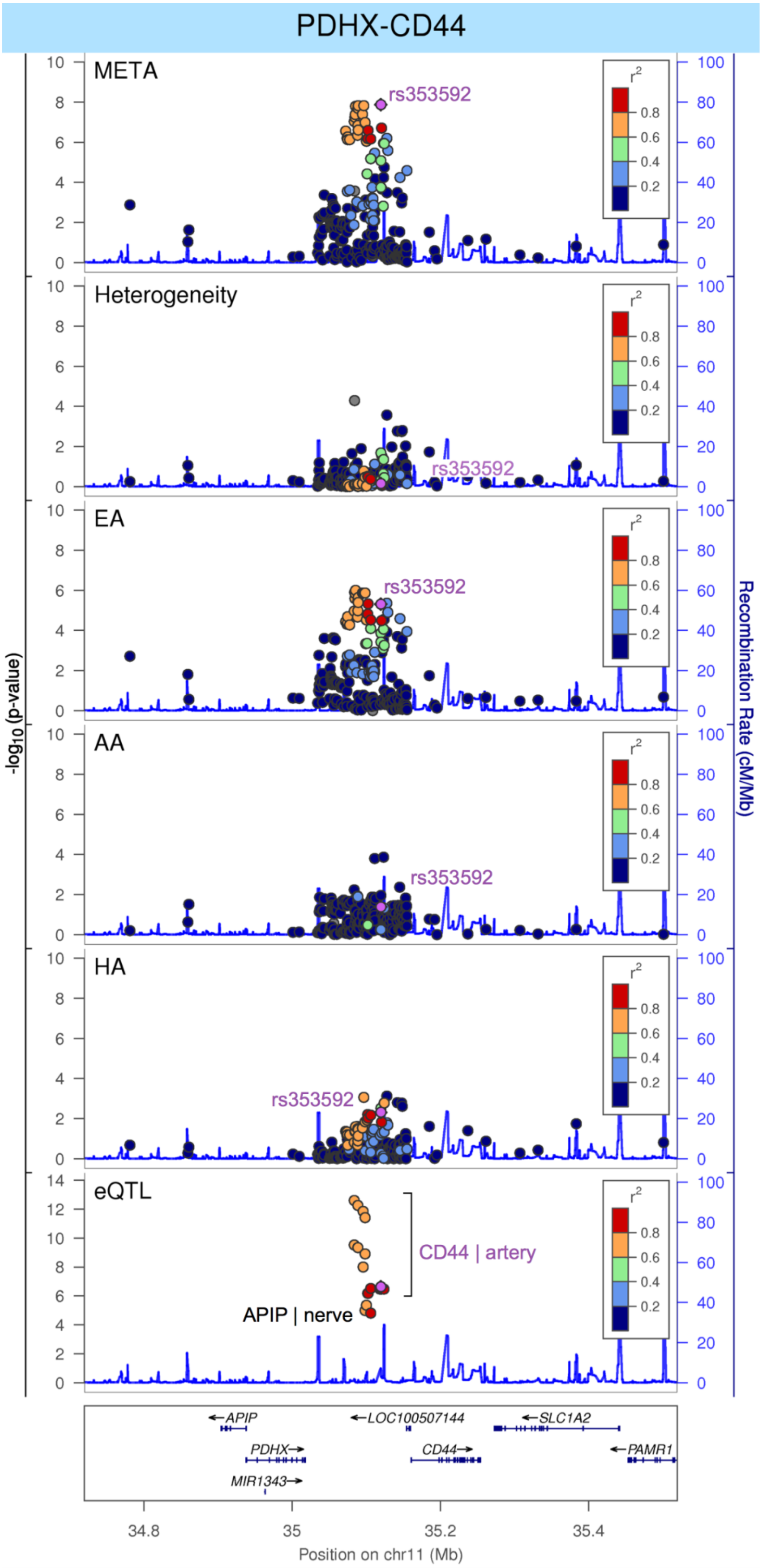

PDHX-CD44
META
rs353592
Heterogeneity
rs353592
EA
rs353592
AA
rs353592
HA
rs353592
eQTL
CD44 | artery
APIP | nerve
-log10 (p-value)
Recombination Rate (cM/Mb)
r2
0.8
0.6
0.4
0.2
APIP
LOC100507144
SLC1A2
PDHX
CD44
PAMR1
MIR1343
34.8
35
35.2
35.4
Position on chr11 (Mb)

j. SLC15A4

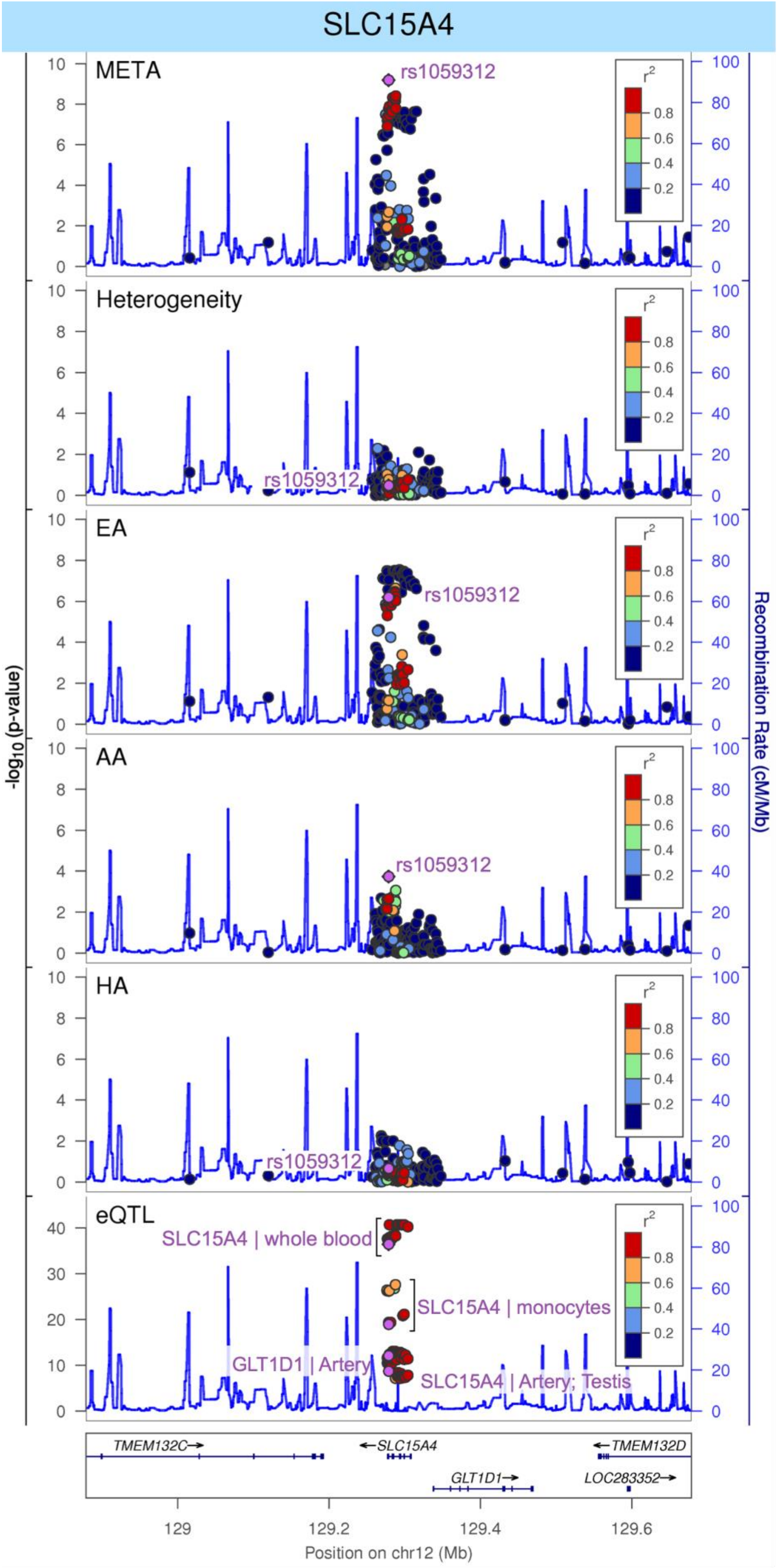
SLC15A4
META
rs1059312
Heterogeneity
rs1059312
EA
rs1059312
AA
rs1059312
HA
rs1059312
eQTL
SLC15A4 | whole blood
SLC15A4 | monocytes
GLT1D1 | Artery
SLC15A4 | Artery; Testis
$-\log_{10}$(p-value)
Recombination Rate (cM/Mb)
r^2
TMEM132C
SLC15A4
TMEM132D
GLT1D1
LOC283352
Position on chr12 (Mb)

k. NCOA5-CD40

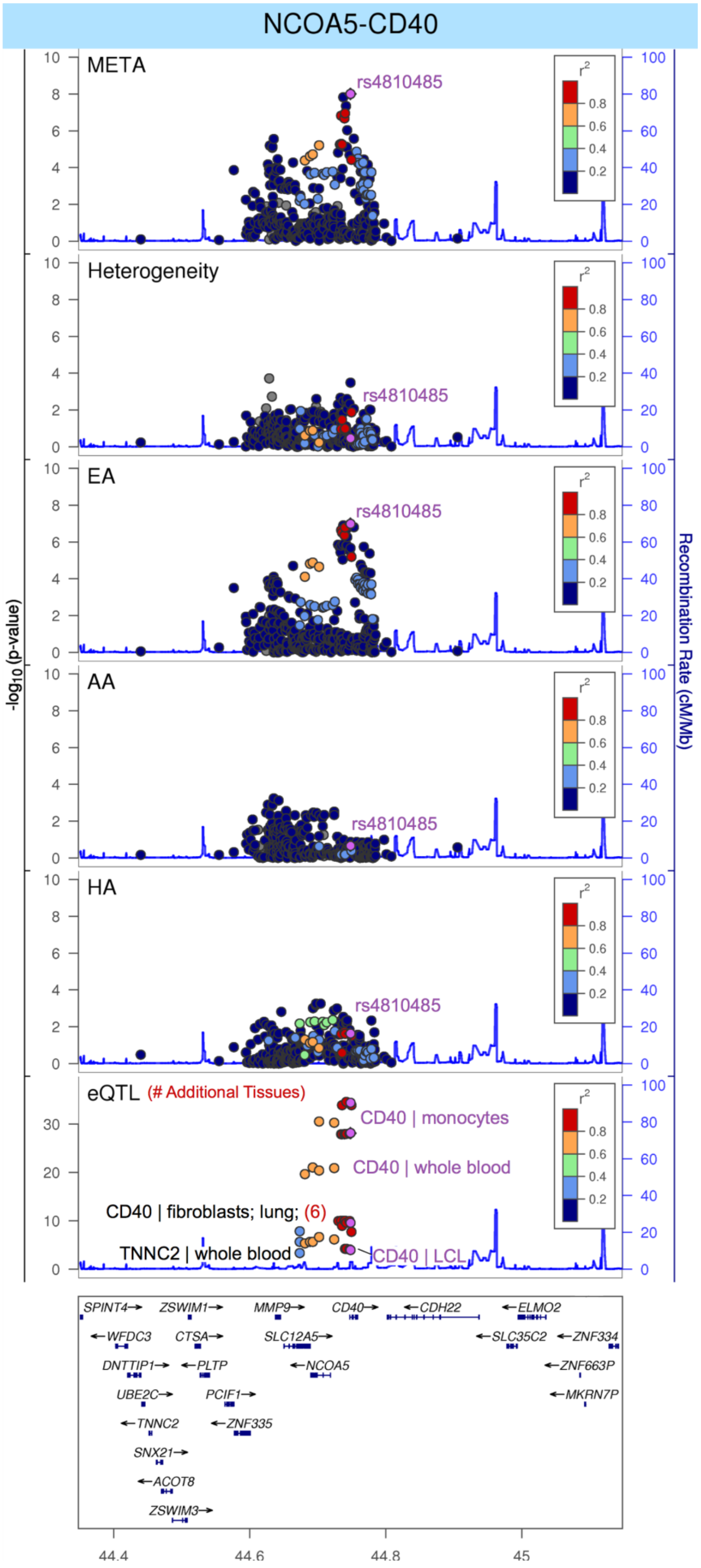

NCOA5-CD40
META
Heterogeneity
EA
AA
HA
eQTL (# Additional Tissues)
rs4810485
CD40 | monocytes
CD40 | whole blood
CD40 | fibroblasts; lung; (6)
TNNC2 | whole blood
CD40 | LCL
-log10 (p-value)
Recombination Rate (cM/Mb)
r²
SPINT4
ZSWIM1
MMP9
CD40
CDH22
ELMO2
WFDC3
CTSA
SLC12A5
SLC35C2
ZNF334
DNTTIP1
PLTP
NCOA5
ZNF663P
UBE2C
PCIF1
MKRN7P
TNNC2
ZNF335
SNX21
ACOT8
ZSWIM3
44.4
44.6
44.8
45

Supplementary Figure 16. Plots of additional meta-analysis regions noted for transancestral-mapping (p-value$_{fdr}$ <0.001). Plots appear in the order found in Supplementary Data 8. Individual plots were created using LocusZoom standalone software (*http://locuszoom.sph.umich.edu/*).

a. VANGL2

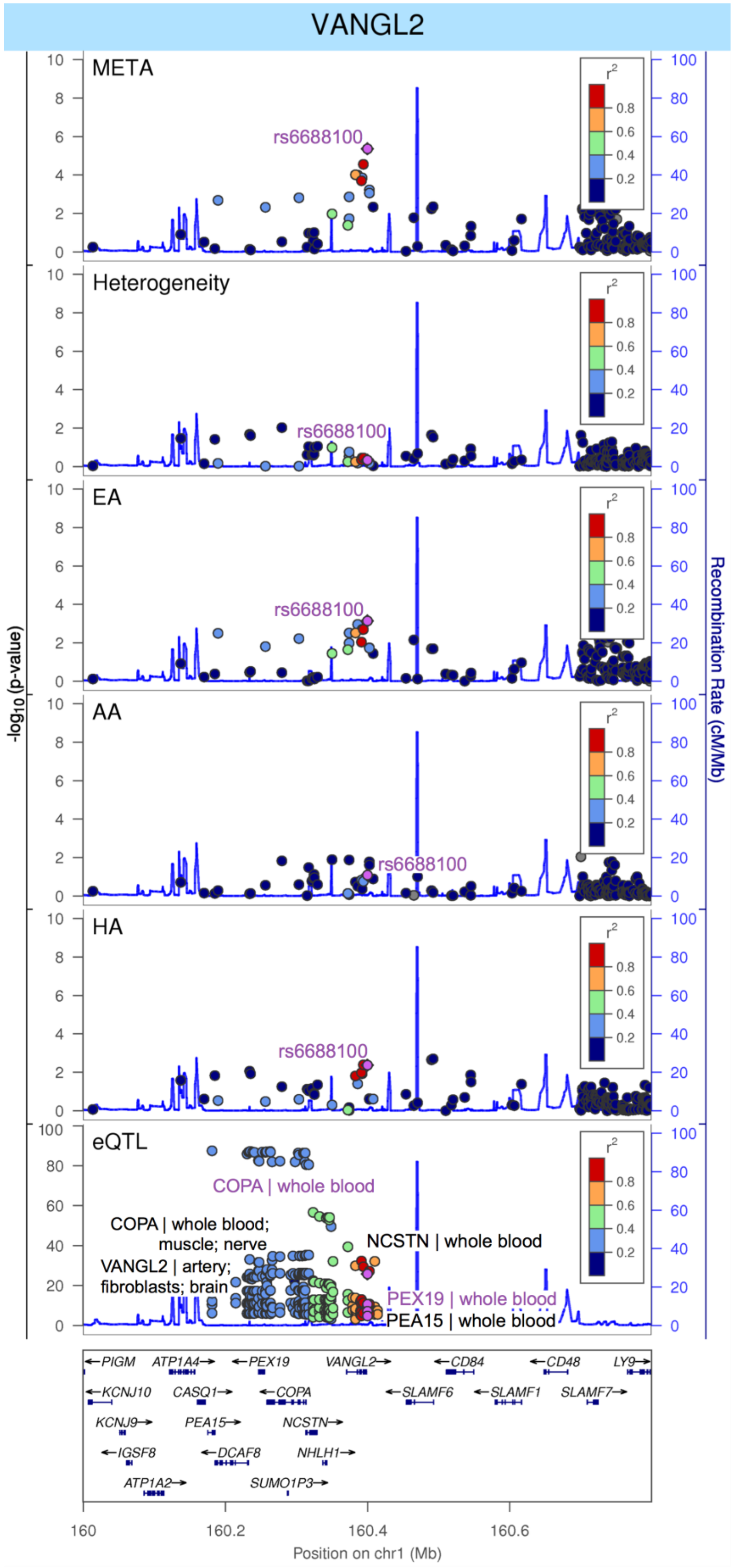

b. FCGR2A

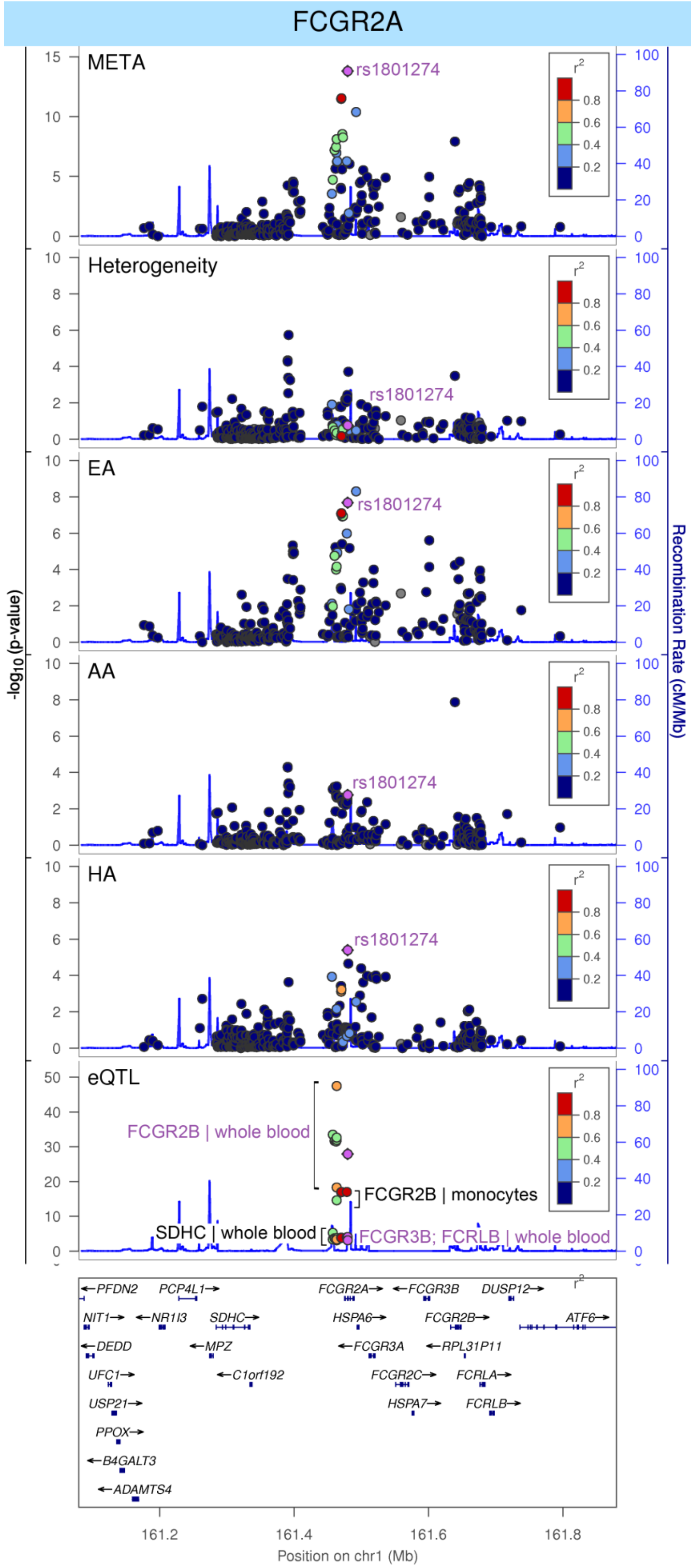

FCGR2A
META
rs1801274
Heterogeneity
rs1801274
EA
rs1801274
AA
rs1801274
HA
rs1801274
eQTL
FCGR2B | whole blood
FCGR2B | monocytes
SDHC | whole blood
FCGR3B; FCRLB | whole blood
r^2
-log10 (p-value)
Recombination Rate (cM/Mb)
PFDN2
PCP4L1
FCGR2A
FCGR3B
DUSP12
NIT1
NR1I3
SDHC
HSPA6
FCGR2B
ATF6
DEDD
MPZ
FCGR3A
RPL31P11
UFC1
C1orf192
FCGR2C
FCRLA
USP21
HSPA7
FCRLB
PPOX
B4GALT3
ADAMTS4
161.2
161.4
161.6
161.8
Position on chr1 (Mb)

c. RGS1

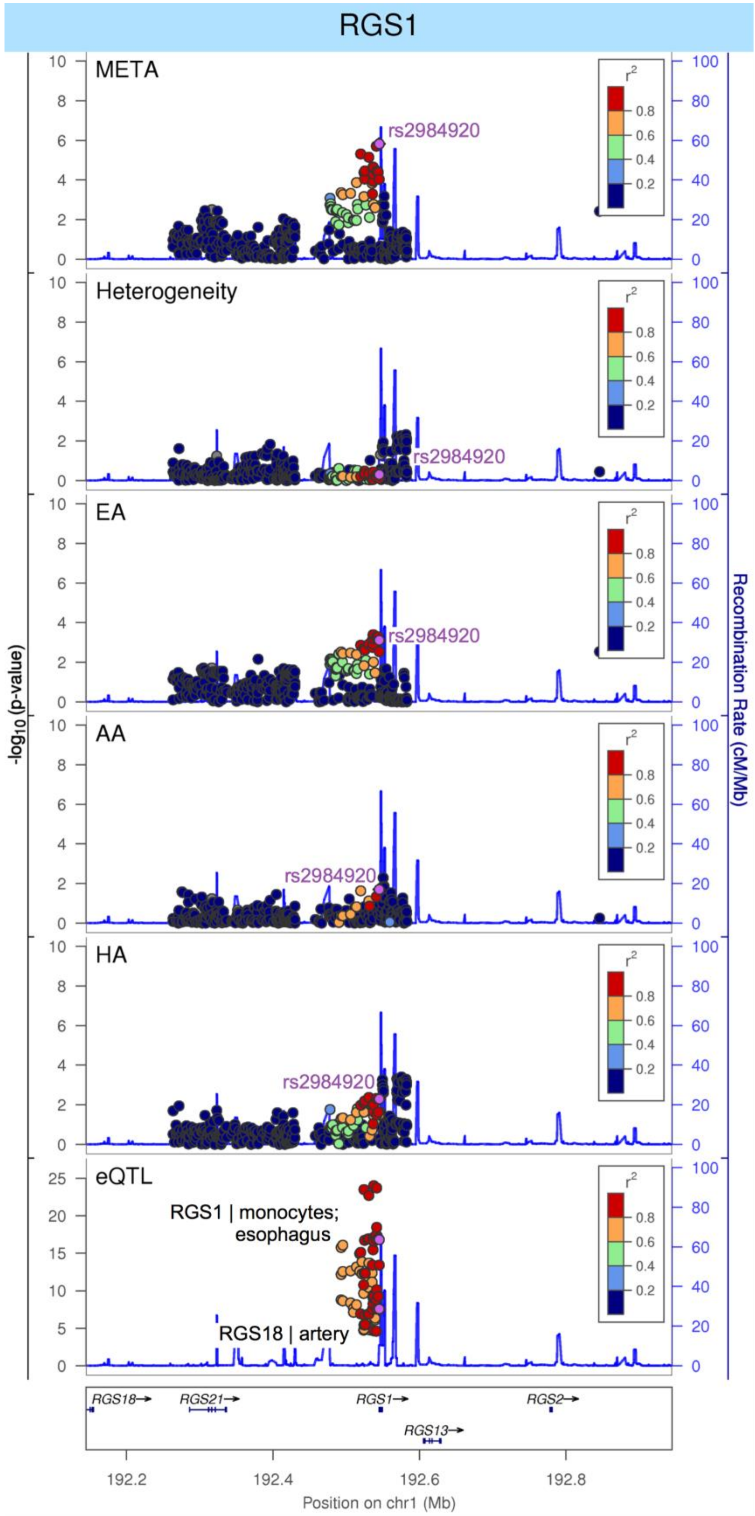

RGS1
META
Heterogeneity
EA
AA
HA
eQTL
rs2984920
RGS1 | monocytes;
esophagus
RGS18 | artery
-log10 (p-value)
Recombination Rate (cM/Mb)
r2
0.8
0.6
0.4
0.2
RGS18→
RGS21→
RGS1→
RGS2→
RGS13→
192.2
192.4
192.6
192.8
Position on chr1 (Mb)

d. IL10

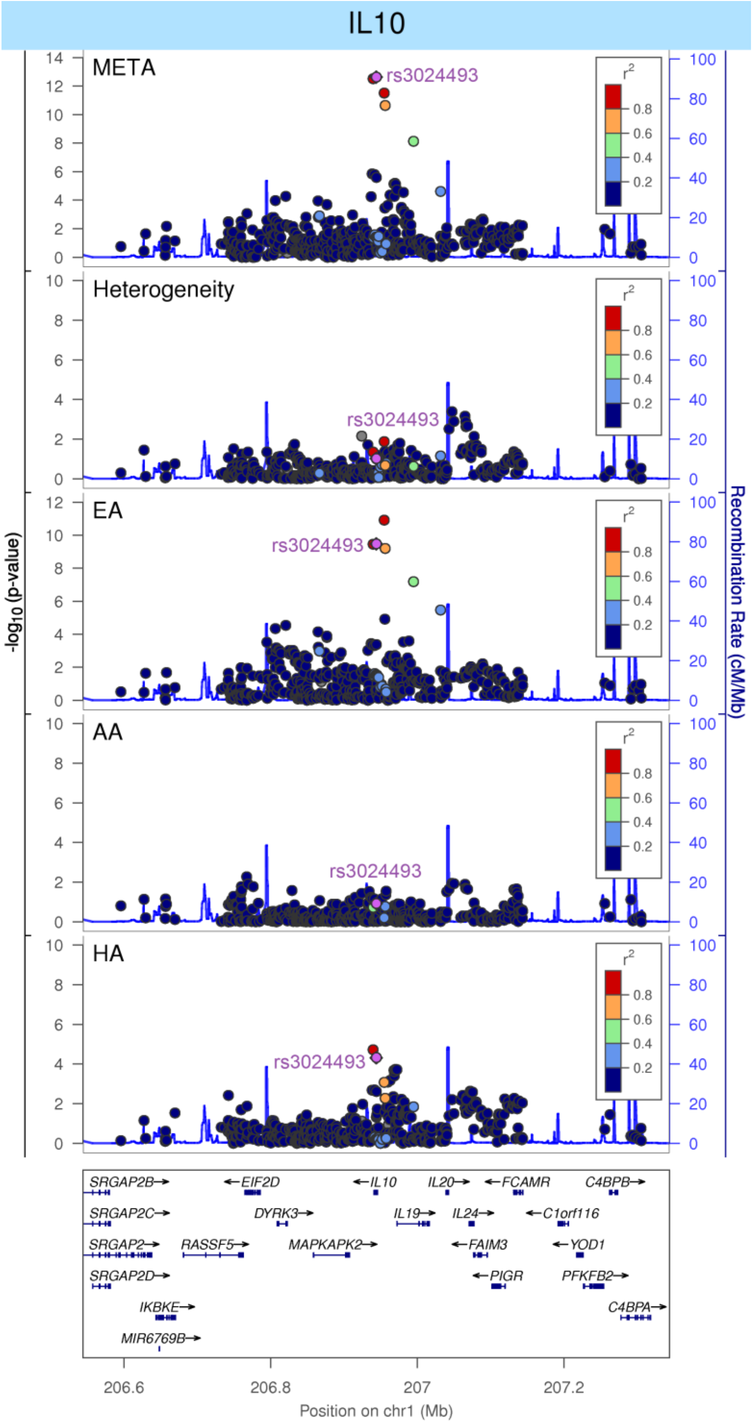

IL10
META
rs3024493
Heterogeneity
rs3024493
EA
rs3024493
AA
rs3024493
HA
rs3024493
-log10 (p-value)
Recombination Rate (cM/Mb)
SRGAP2B
EIF2D
IL10
IL20
FCAMR
C4BPB
SRGAP2C
DYRK3
IL19
IL24
C1orf116
SRGAP2
RASSF5
MAPKAPK2
FAIM3
YOD1
SRGAP2D
PIGR
PFKFB2
IKBKE
C4BPA
MIR6769B
206.6
206.8
207
207.2
Position on chr1 (Mb)

e. STAT4

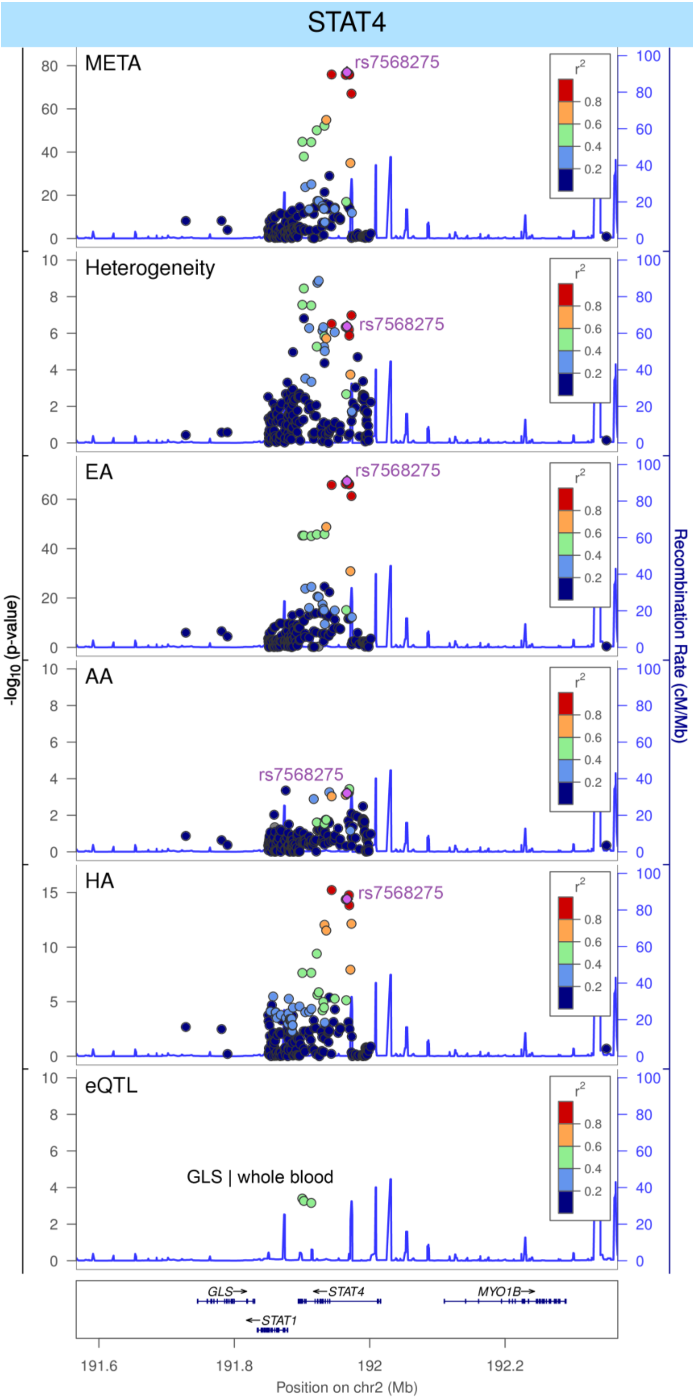

STAT4
META
Heterogeneity
EA
AA
HA
eQTL
rs7568275
GLS | whole blood
r²
0.8
0.6
0.4
0.2
-log10 (p-value)
Recombination Rate (cM/Mb)
GLS
STAT4
STAT1
MYO1B
191.6
191.8
192
192.2
Position on chr2 (Mb)

f. PXK-PDHB-KCTD6

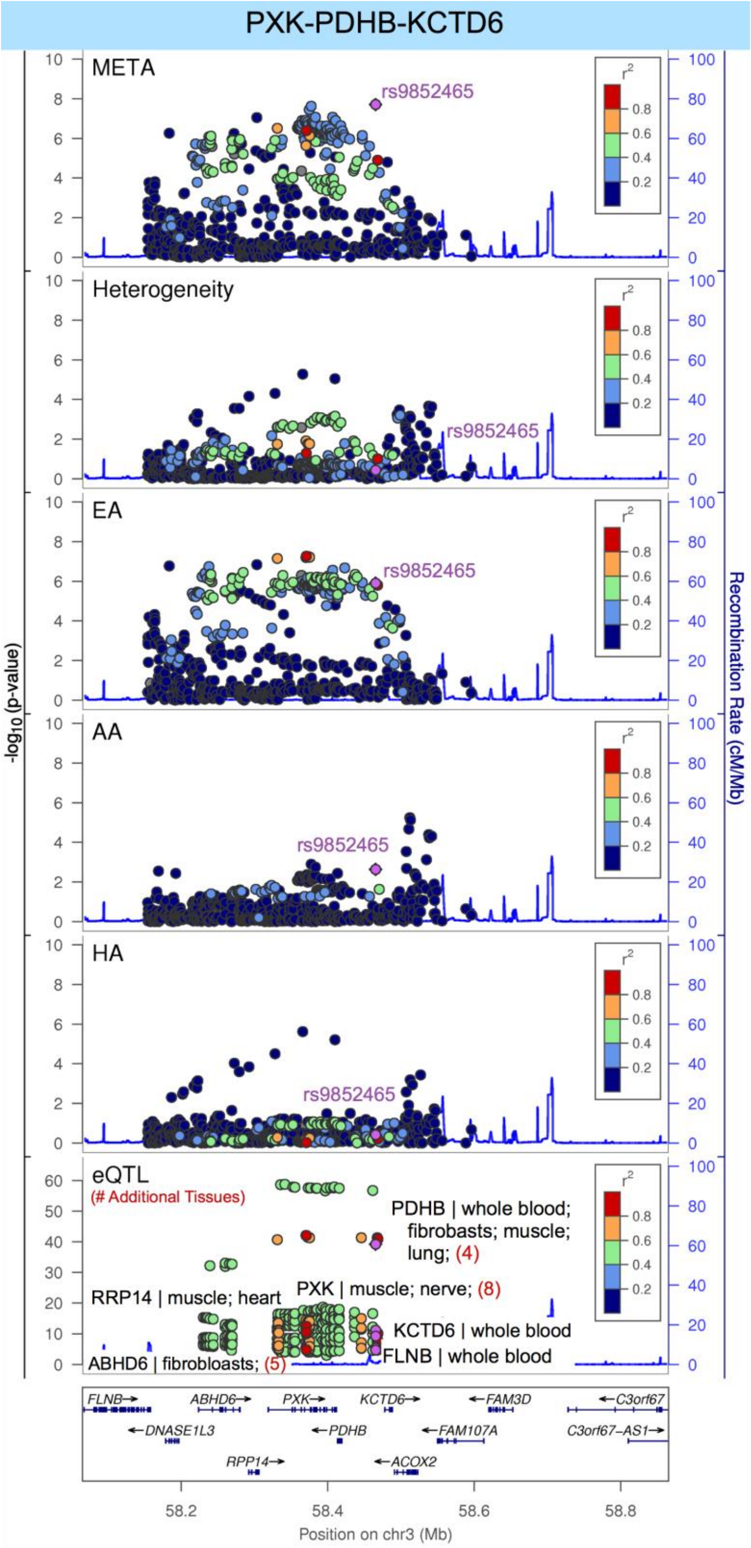

g. TMEM39A-TIMMDC1

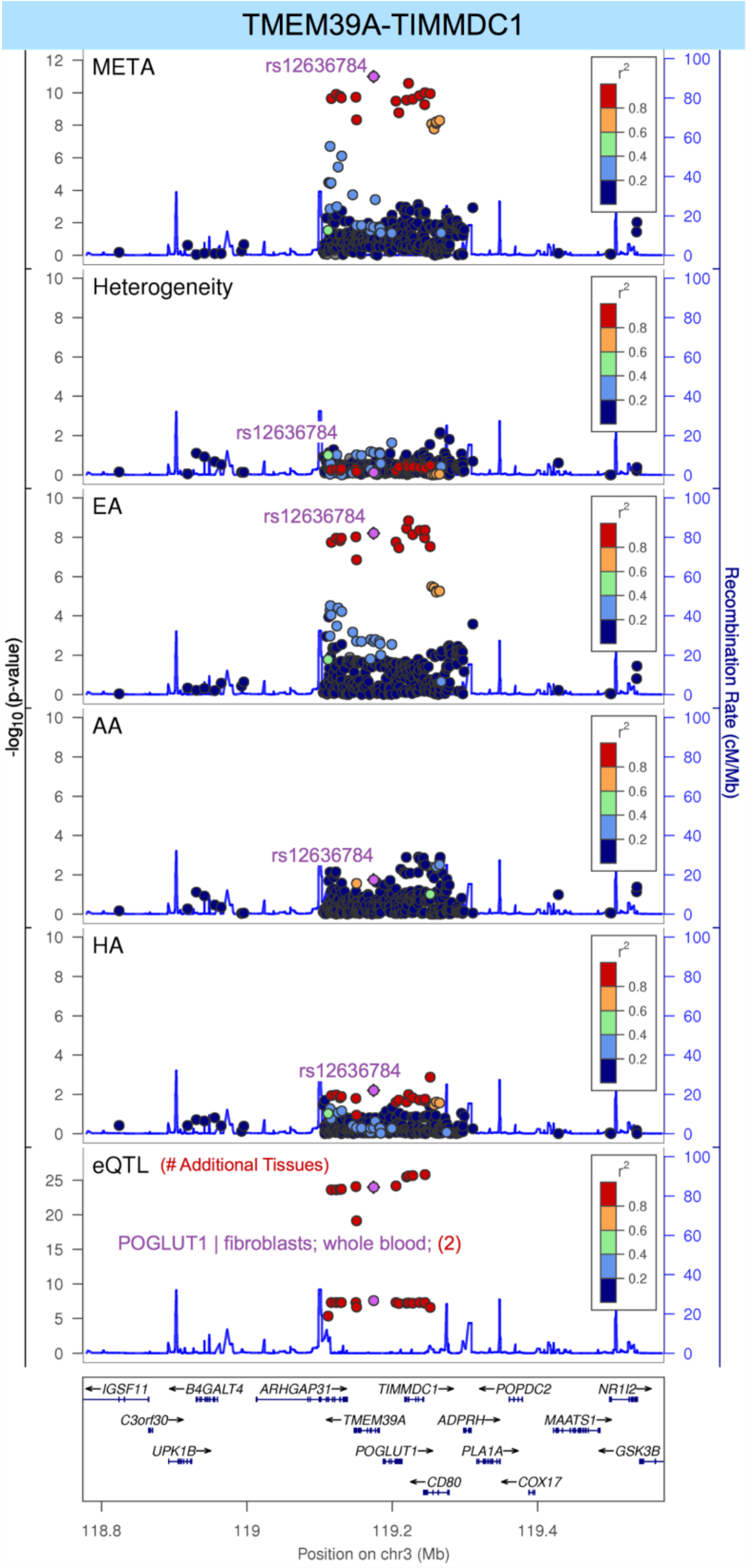

h. BANK1

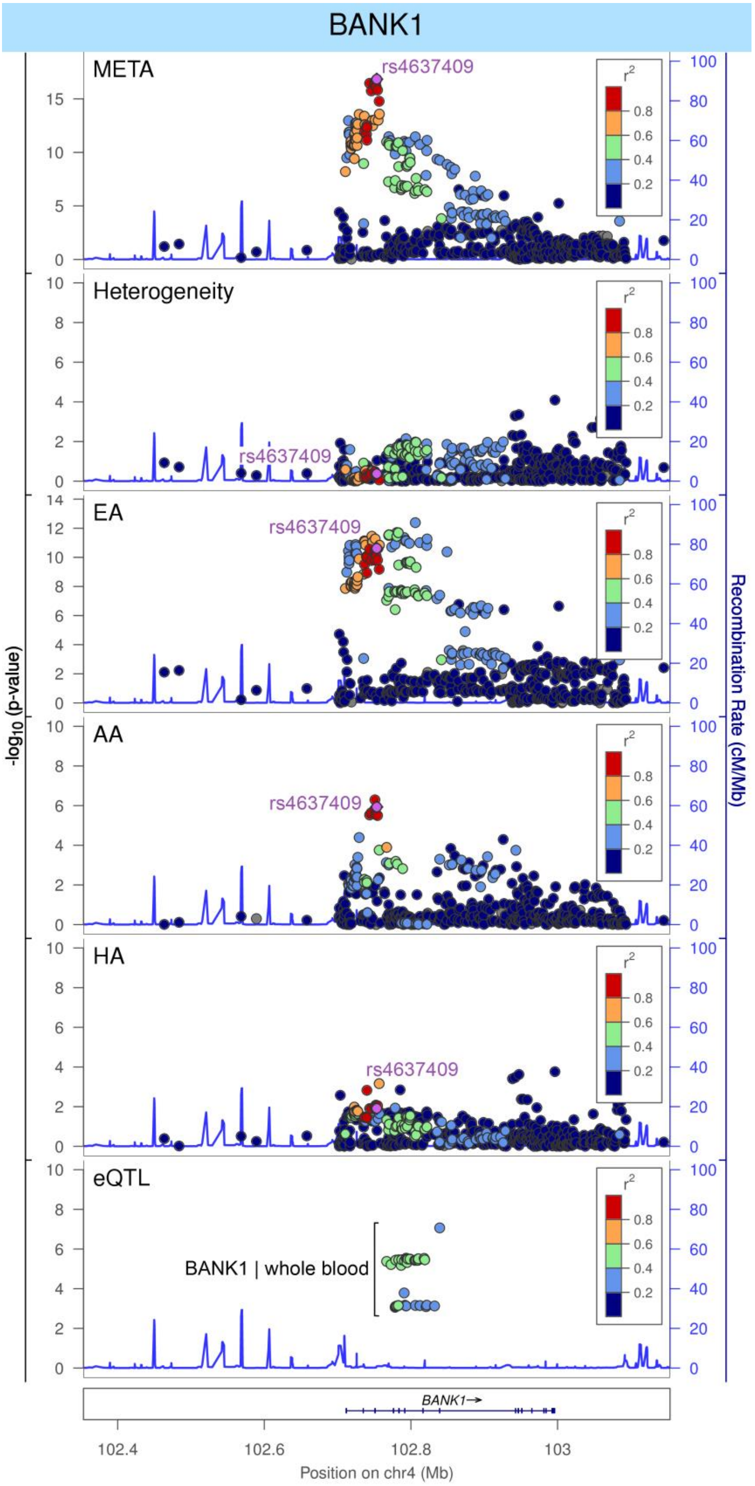

BANK1
META
Heterogeneity
EA
AA
HA
eQTL
rs4637409
BANK1 | whole blood
r²
0.8
0.6
0.4
0.2
-log10 (p-value)
Recombination Rate (cM/Mb)
BANK1→
102.4
102.6
102.8
103
Position on chr4 (Mb)

i. TNIP1

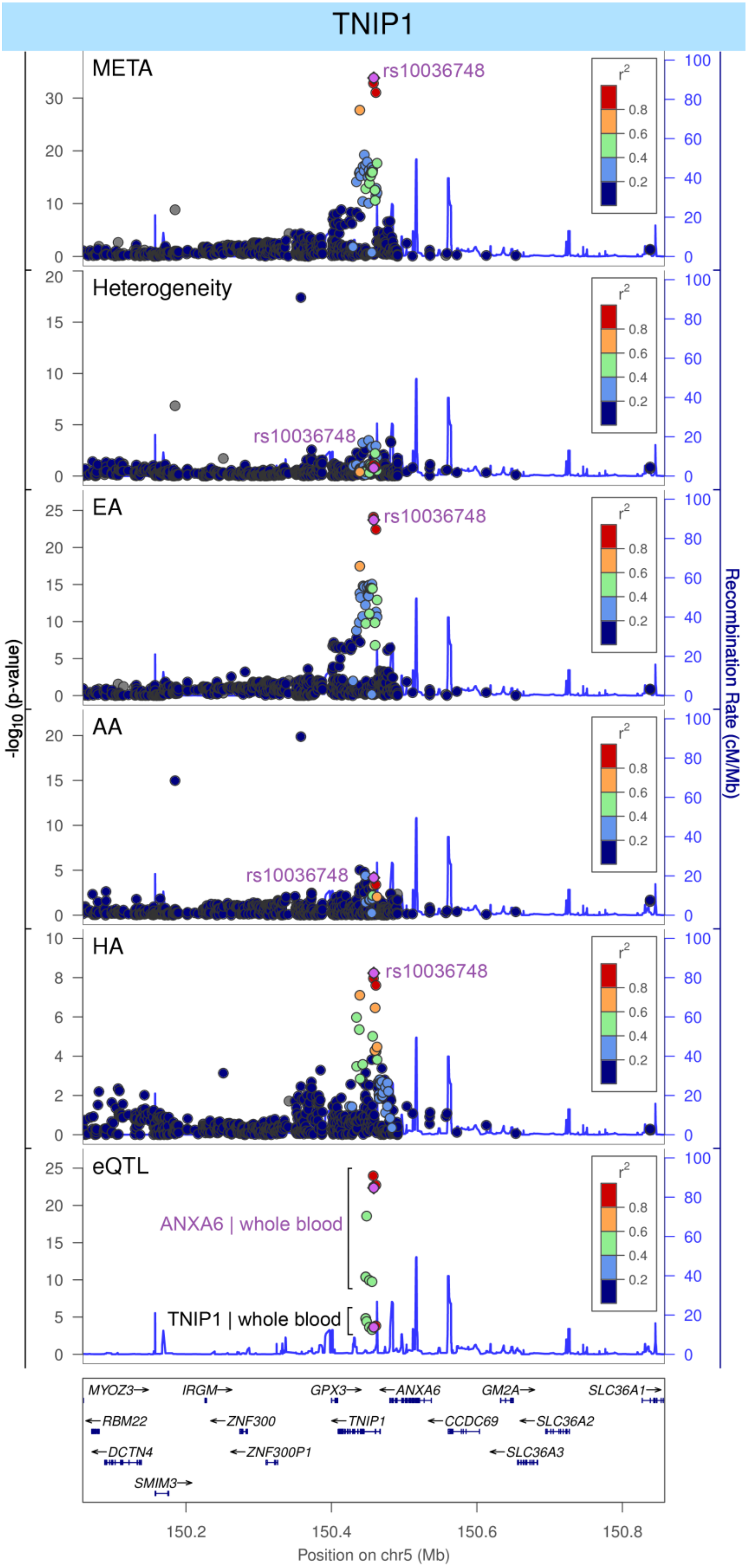

TNIP1
META
rs10036748
Heterogeneity
rs10036748
EA
rs10036748
AA
rs10036748
HA
rs10036748
eQTL
ANXA6 | whole blood
TNIP1 | whole blood
r2
0.8
0.6
0.4
0.2
-log10 (p-value)
Recombination Rate (cM/Mb)
MYOZ3
IRGM
GPX3
ANXA6
GM2A
SLC36A1
RBM22
ZNF300
TNIP1
CCDC69
SLC36A2
DCTN4
ZNF300P1
SLC36A3
SMIM3
150.2
150.4
150.6
150.8
Position on chr5 (Mb)

j. ATG5

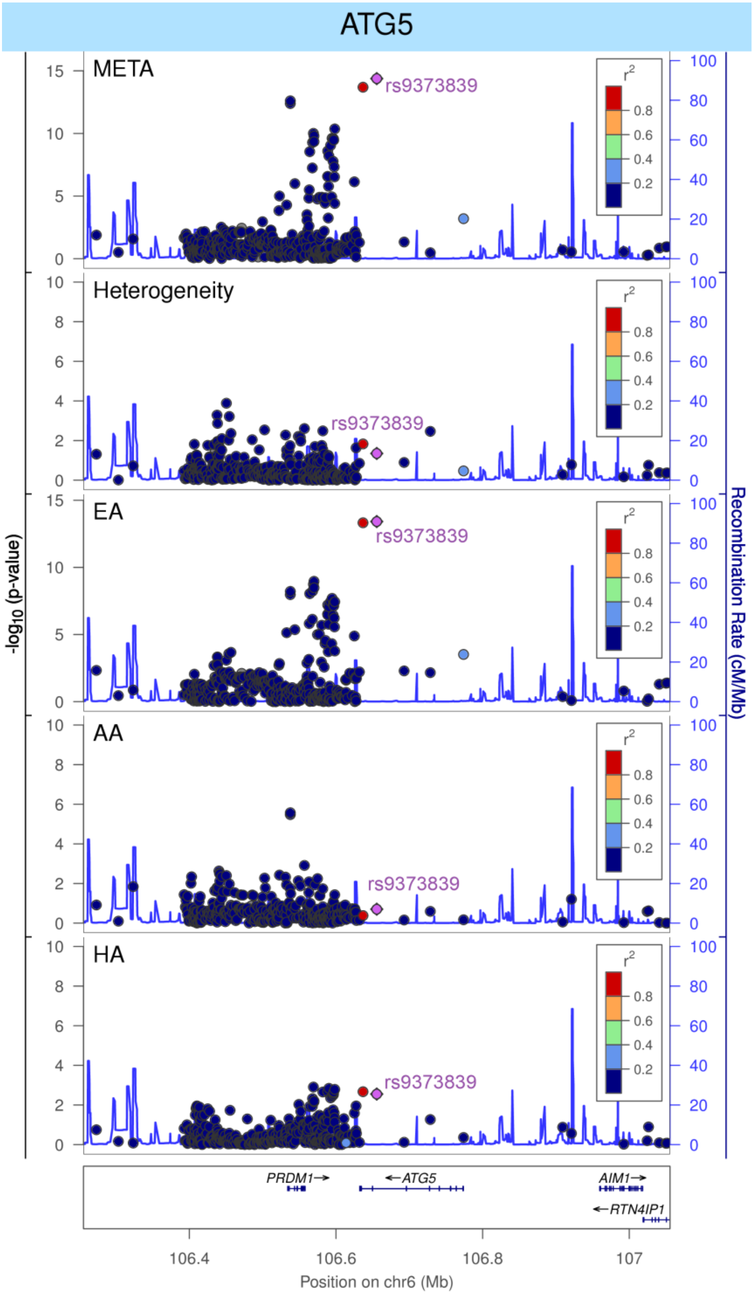

ATG5
META
rs9373839
Heterogeneity
rs9373839
EA
rs9373839
AA
rs9373839
HA
rs9373839
-log10 (p-value)
Recombination Rate (cM/Mb)
r2
0.8
0.6
0.4
0.2
PRDM1
ATG5
AIM1
RTN4IP1
106.4
106.6
106.8
107
Position on chr6 (Mb)

k. TAGAP

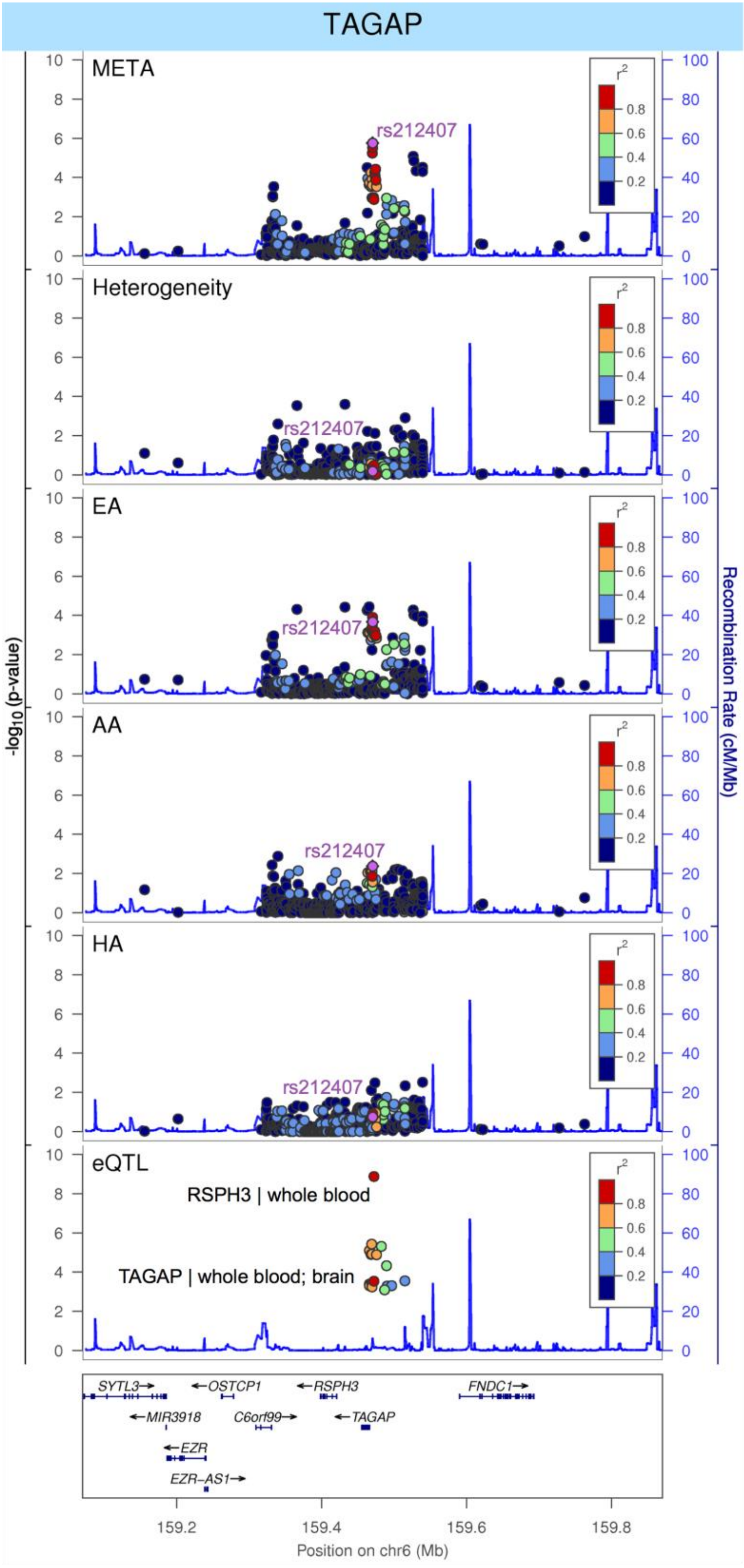
TAGAP
META
Heterogeneity
EA
AA
HA
eQTL
rs212407
RSPH3 | whole blood
TAGAP | whole blood; brain
-log10 (p-value)
Recombination Rate (cM/Mb)
r2
SYTL3
OSTCP1
RSPH3
FNDC1
MIR3918
C6orf99
TAGAP
EZR
EZR-AS1
Position on chr6 (Mb)

I. JAZF1

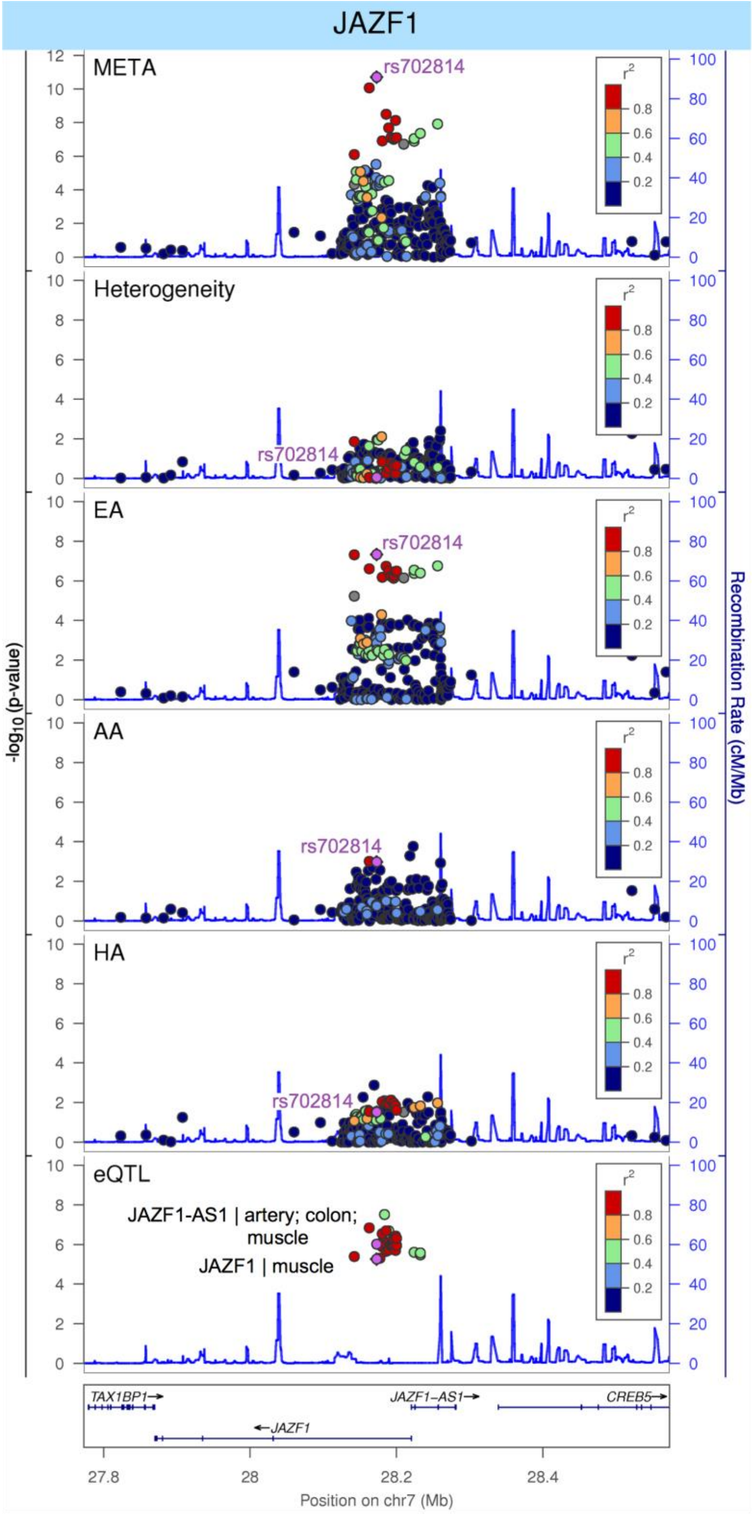
JAZF1
META
Heterogeneity
EA
AA
HA
eQTL
rs702814
JAZF1-AS1 | artery; colon; muscle
JAZF1 | muscle
-log10 (p-value)
Recombination Rate (cM/Mb)
r2
TAX1BP1
JAZF1-AS1
CREB5
JAZF1
Position on chr7 (Mb)

m. C7orf72-IKZF1

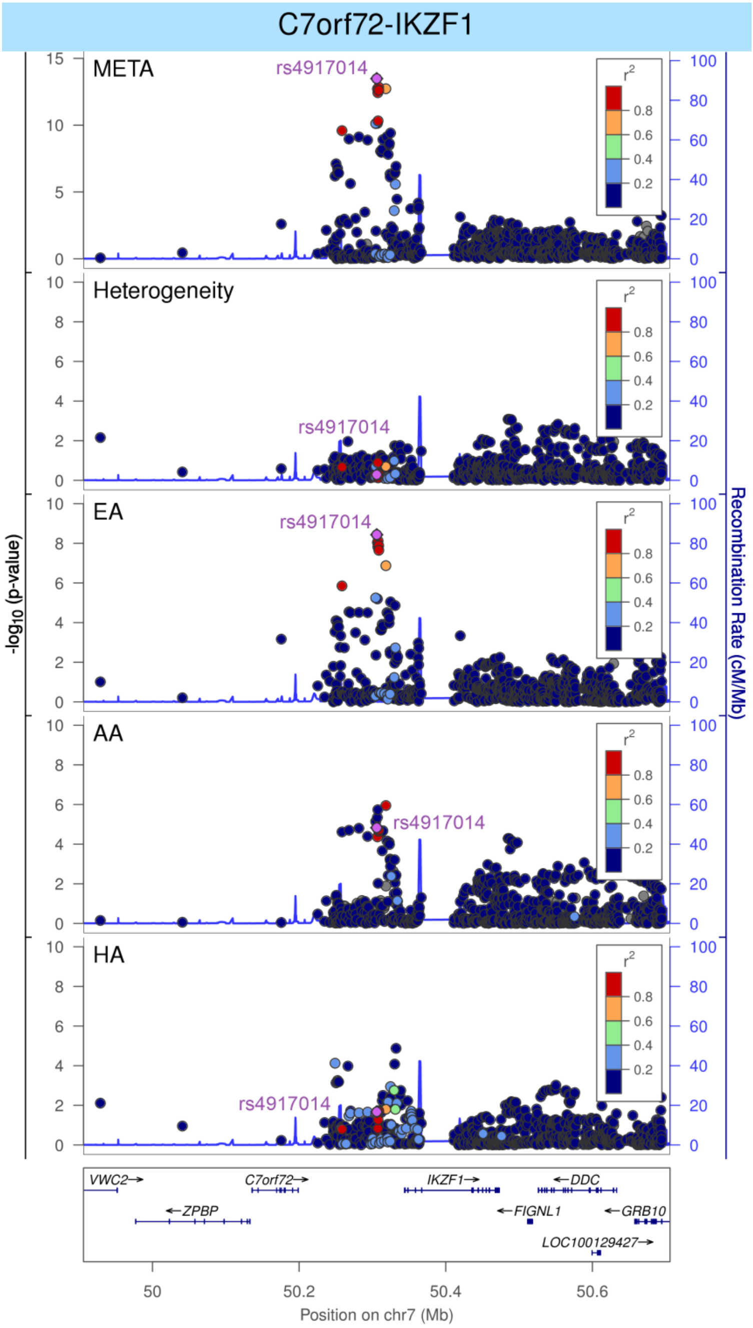

n. BLK

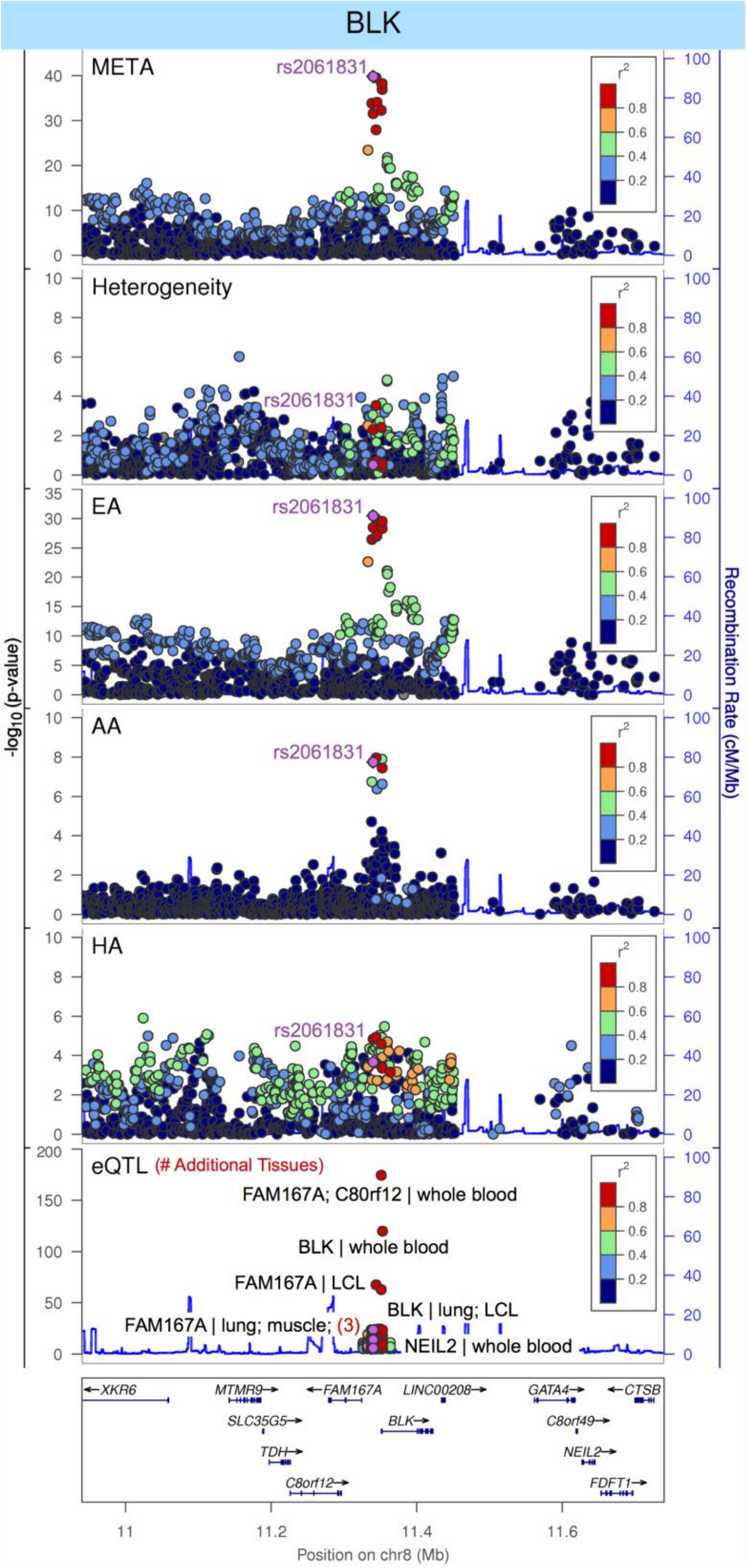
BLK
META
rs2061831
Heterogeneity
EA
AA
HA
eQTL (# Additional Tissues)
FAM167A; C80rf12 | whole blood
BLK | whole blood
FAM167A | LCL
BLK | lung; LCL
FAM167A | lung; muscle; (3)
NEIL2 | whole blood
-log10 (p-value)
Recombination Rate (cM/Mb)
XKR6
MTMR9
FAM167A
LINC00208
GATA4
CTSB
SLC35G5
BLK
C8orf49
TDH
NEIL2
C8orf12
FDFT1
Position on chr8 (Mb)

o. WDFY4

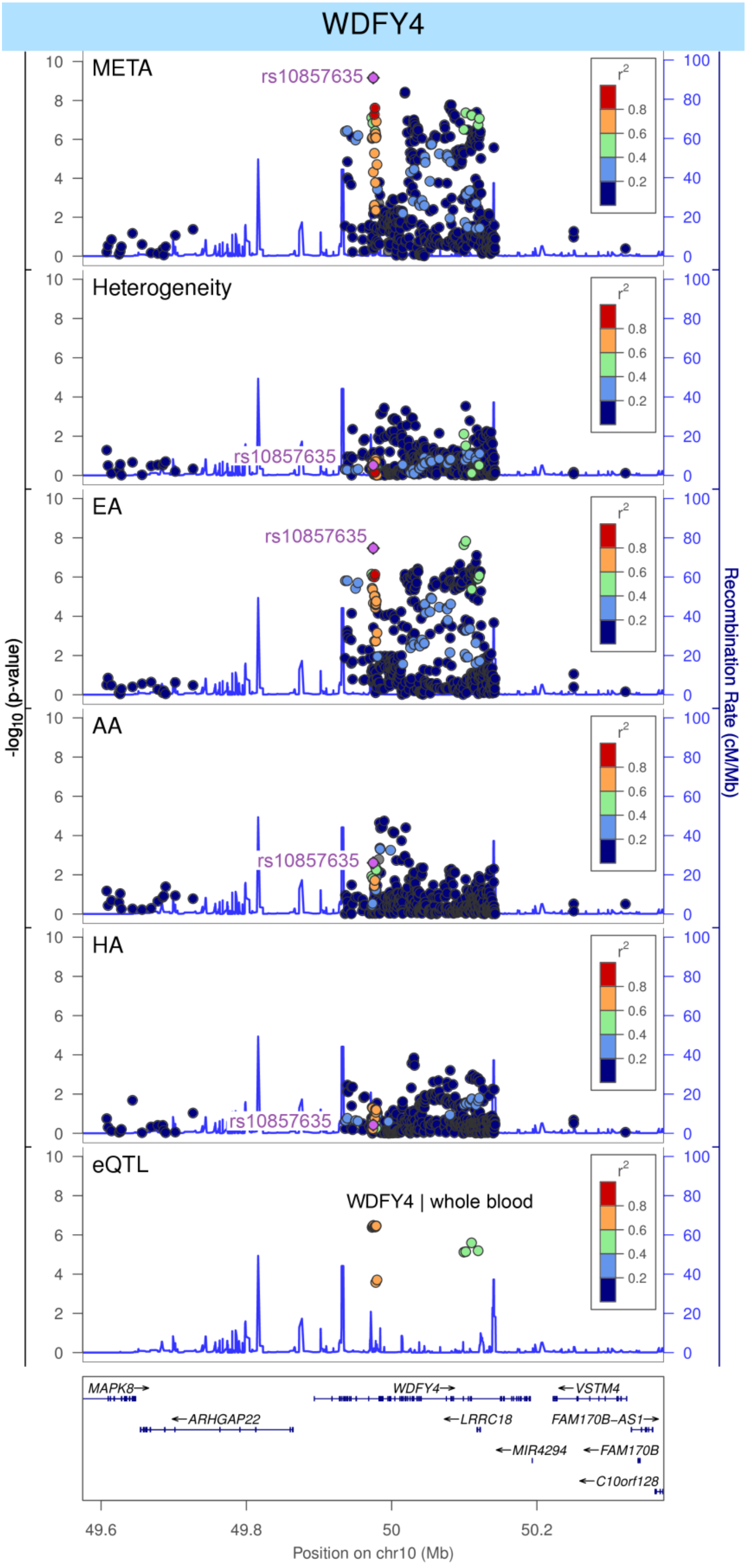

WDFY4
META
rs10857635
Heterogeneity
rs10857635
EA
rs10857635
AA
rs10857635
HA
rs10857635
eQTL
WDFY4 | whole blood
-log10 (p-value)
Recombination Rate (cM/Mb)
MAPK8
WDFY4
VSTM4
ARHGAP22
LRRC18
FAM170B-AS1
MIR4294
FAM170B
C10orf128
49.6
49.8
50
50.2
Position on chr10 (Mb)

p. ETS1

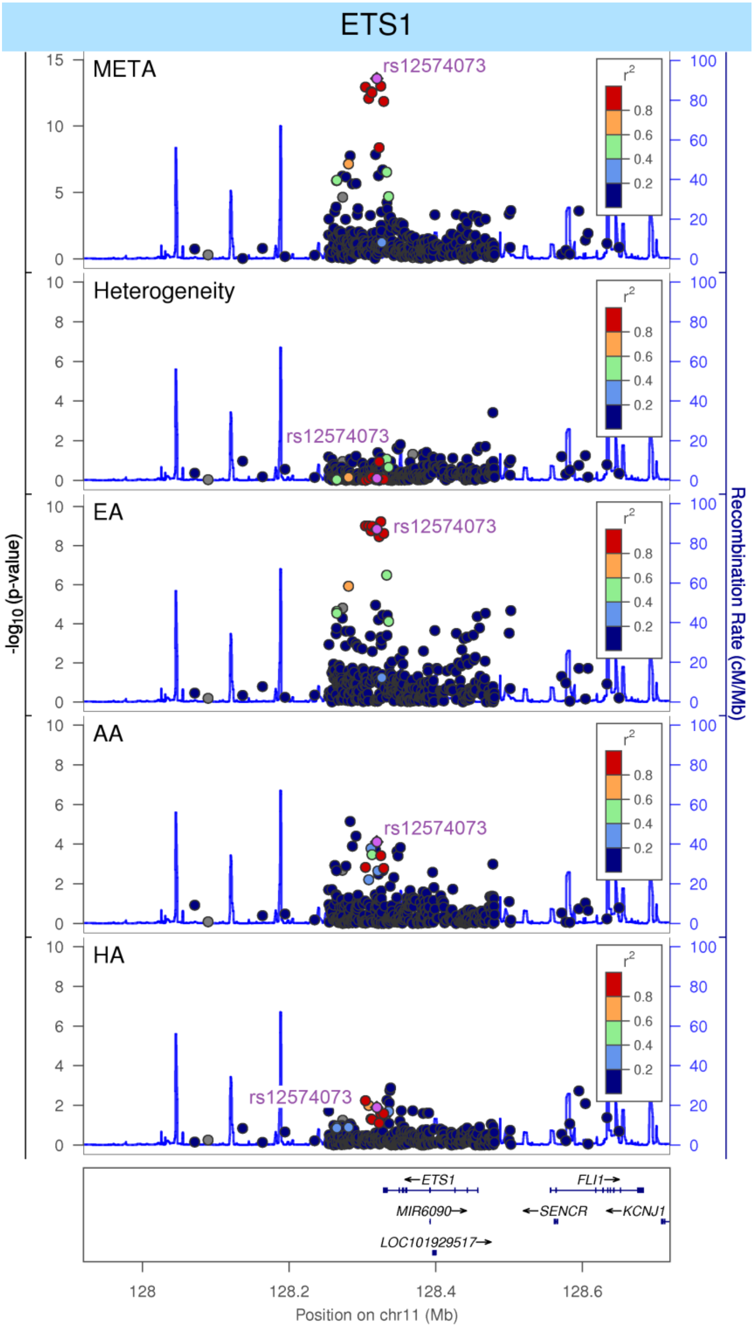

ETS1
META
rs12574073
Heterogeneity
rs12574073
EA
rs12574073
AA
rs12574073
HA
rs12574073
r2
0.8
0.6
0.4
0.2
-log10 (p-value)
Recombination Rate (cM/Mb)
ETS1
FLI1
MIR6090
SENCR
KCNJ1
LOC101929517
128
128.2
128.4
128.6
Position on chr11 (Mb)

q. ATXN2

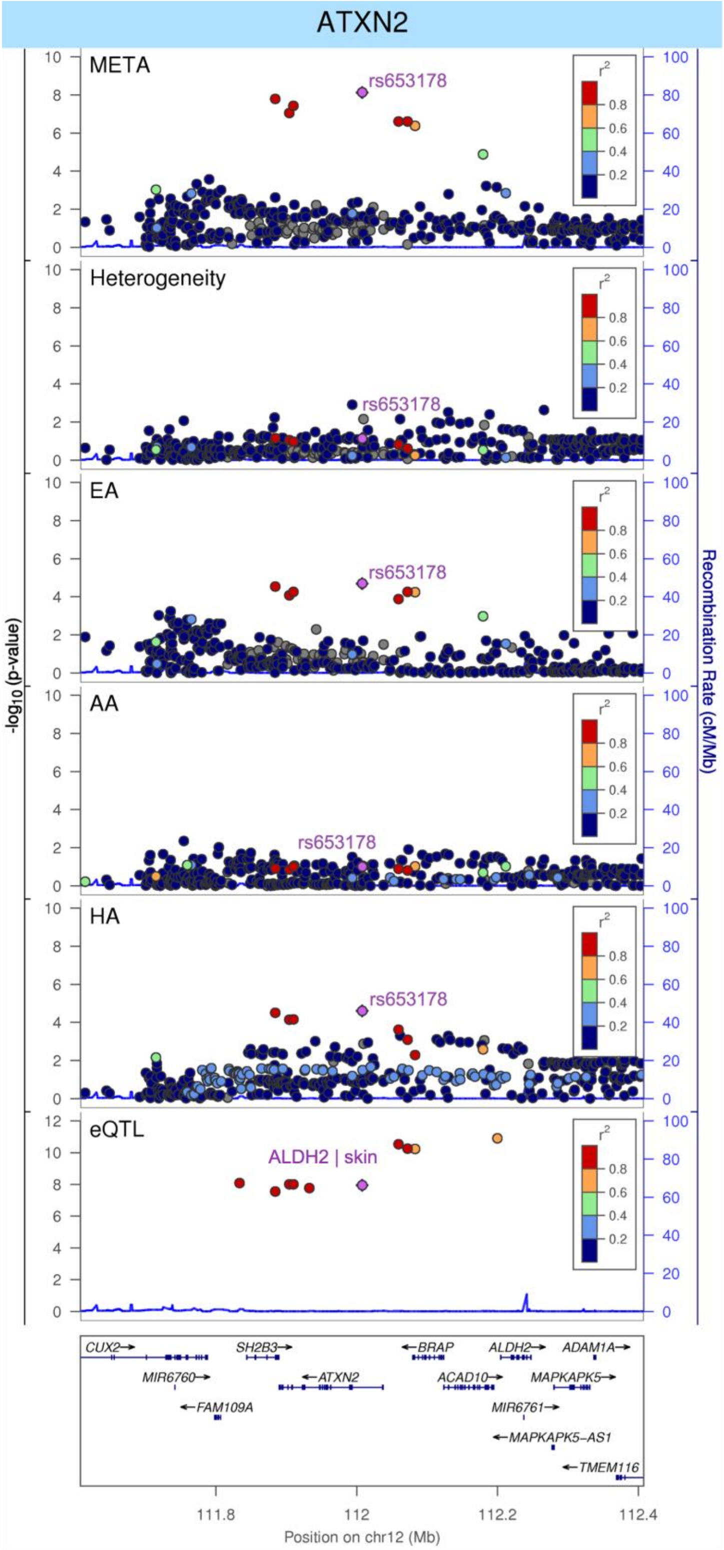

ATXN2
META
Heterogeneity
EA
AA
HA
eQTL
rs653178
ALDH2 | skin
r²
0.8
0.6
0.4
0.2
-log10 (p-value)
Recombination Rate (cM/Mb)
CUX2
SH2B3
BRAP
ALDH2
ADAM1A
MIR6760
ATXN2
ACAD10
MAPKAPK5
FAM109A
MIR6761
MAPKAPK5-AS1
TMEM116
111.8
112
112.2
112.4
Position on chr12 (Mb)

r. ITGAM

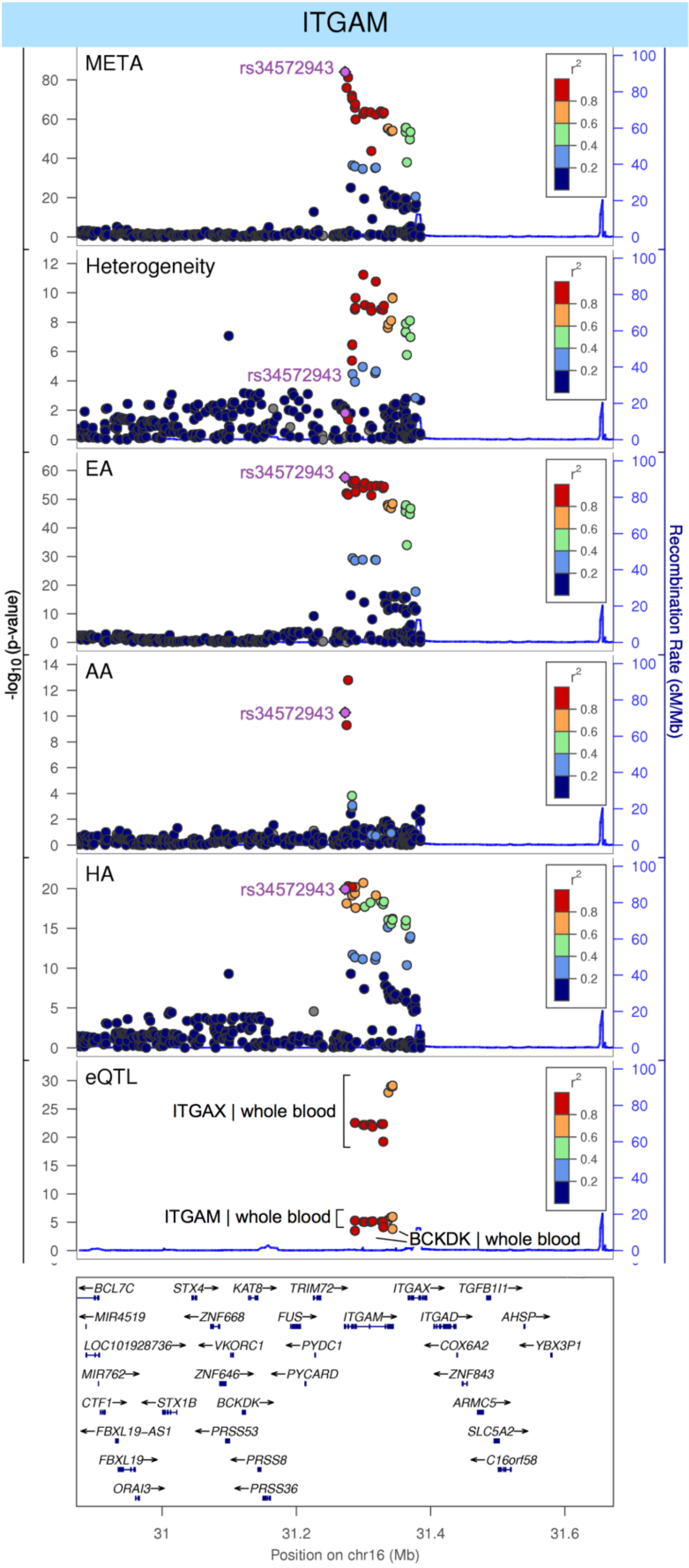

ITGAM
META
rs34572943
Heterogeneity
EA
AA
HA
eQTL
ITGAX | whole blood
ITGAM | whole blood
BCKDK | whole blood
-log10 (p-value)
Recombination Rate (cM/Mb)
r2
Position on chr16 (Mb)

s. UBE2L3

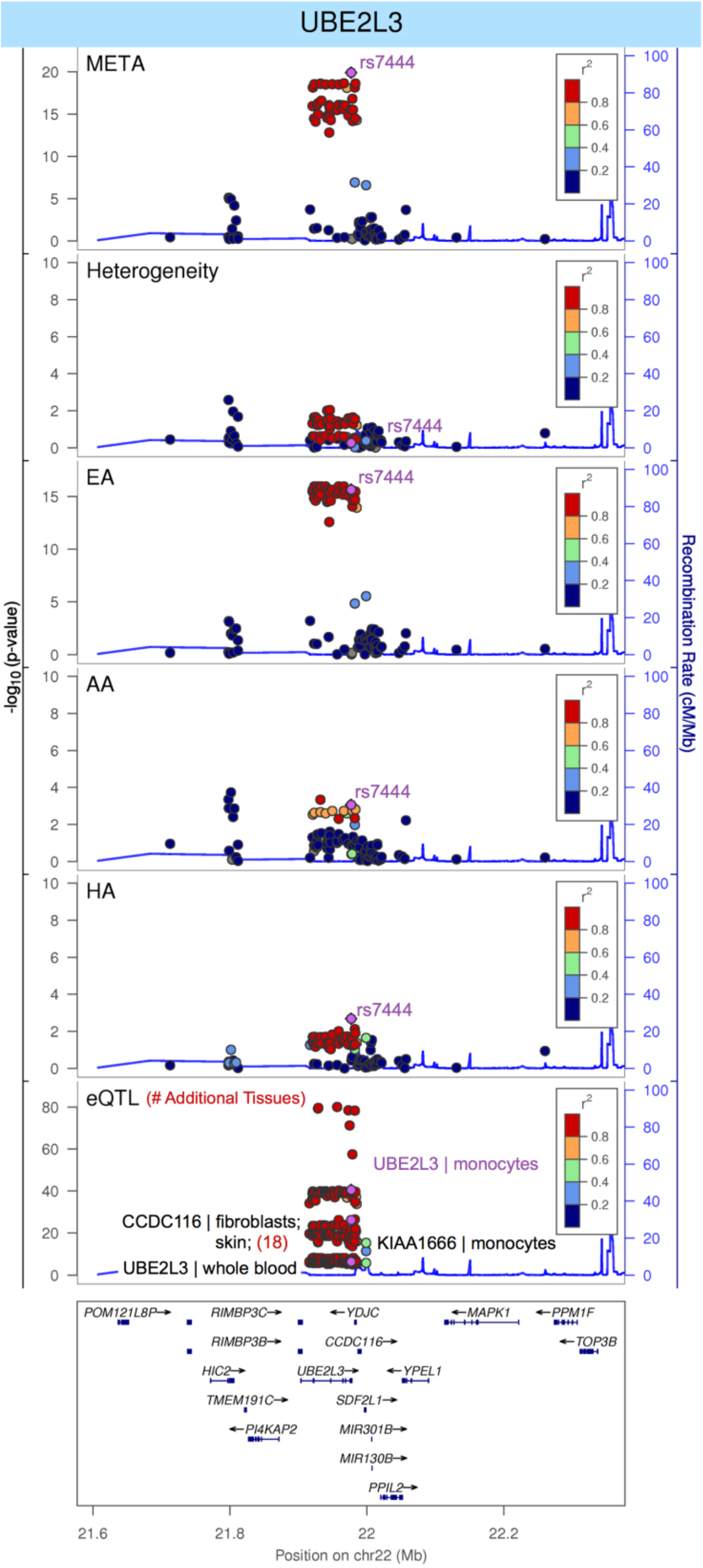
UBE2L3
META
rs7444
Heterogeneity
EA
AA
HA
eQTL (# Additional Tissues)
UBE2L3 | monocytes
CCDC116 | fibroblasts; skin; (18)
KIAA1666 | monocytes
UBE2L3 | whole blood
-log10(p-value)
Recombination Rate (cM/Mb)
r2
POM121L8P
RIMBP3C
YDJC
MAPK1
PPM1F
RIMBP3B
CCDC116
TOP3B
HIC2
UBE2L3
YPEL1
TMEM191C
SDF2L1
PI4KAP2
MIR301B
MIR130B
PPIL2
Position on chr22 (Mb)

Supplementary Figure 17. The non-additive effect of EA risk-allele genetic load on SLE risk (beta values). Plots of the beta (natural logarithm of the odds ratio), instead of the odds ratios, as found in **Figure 5**.

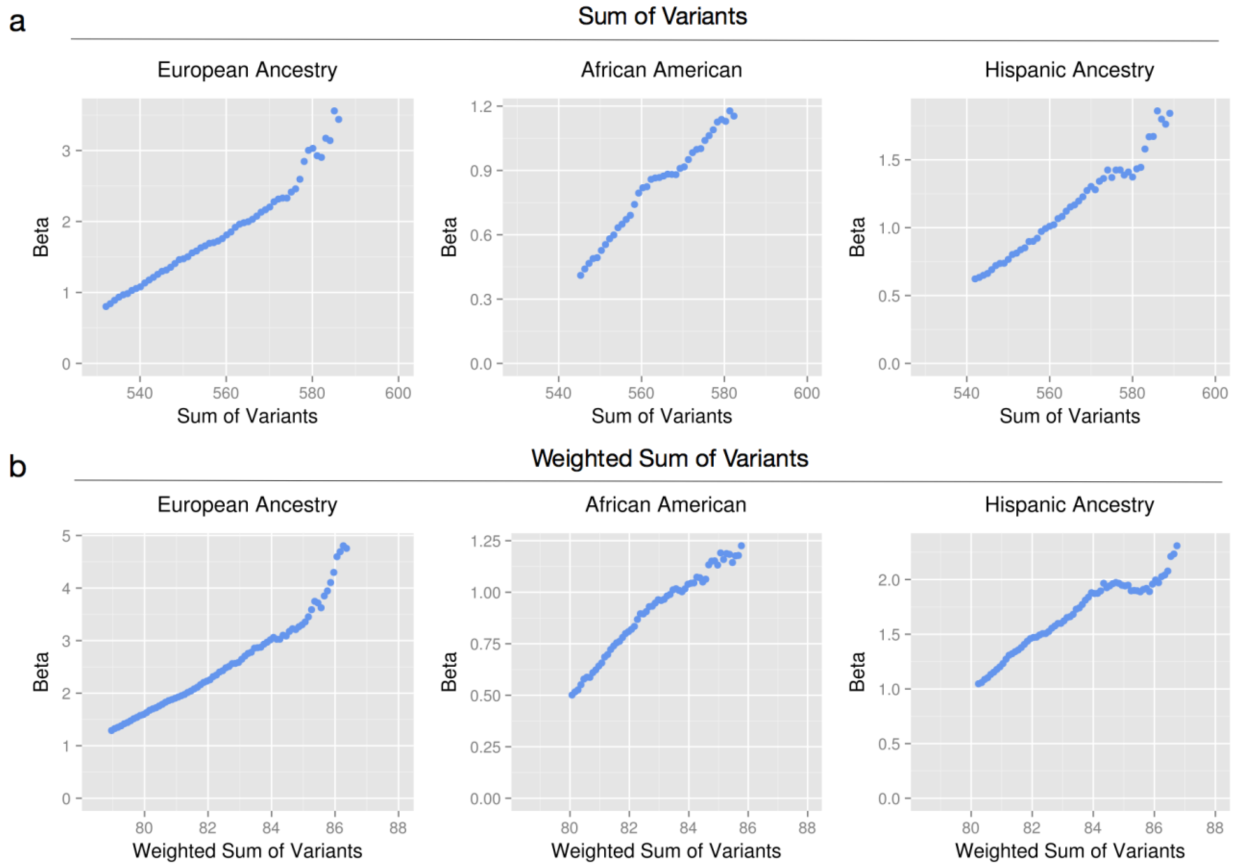

Supplementary Figure 18. Kaplan-Meier curve for age at SLE diagnosis.

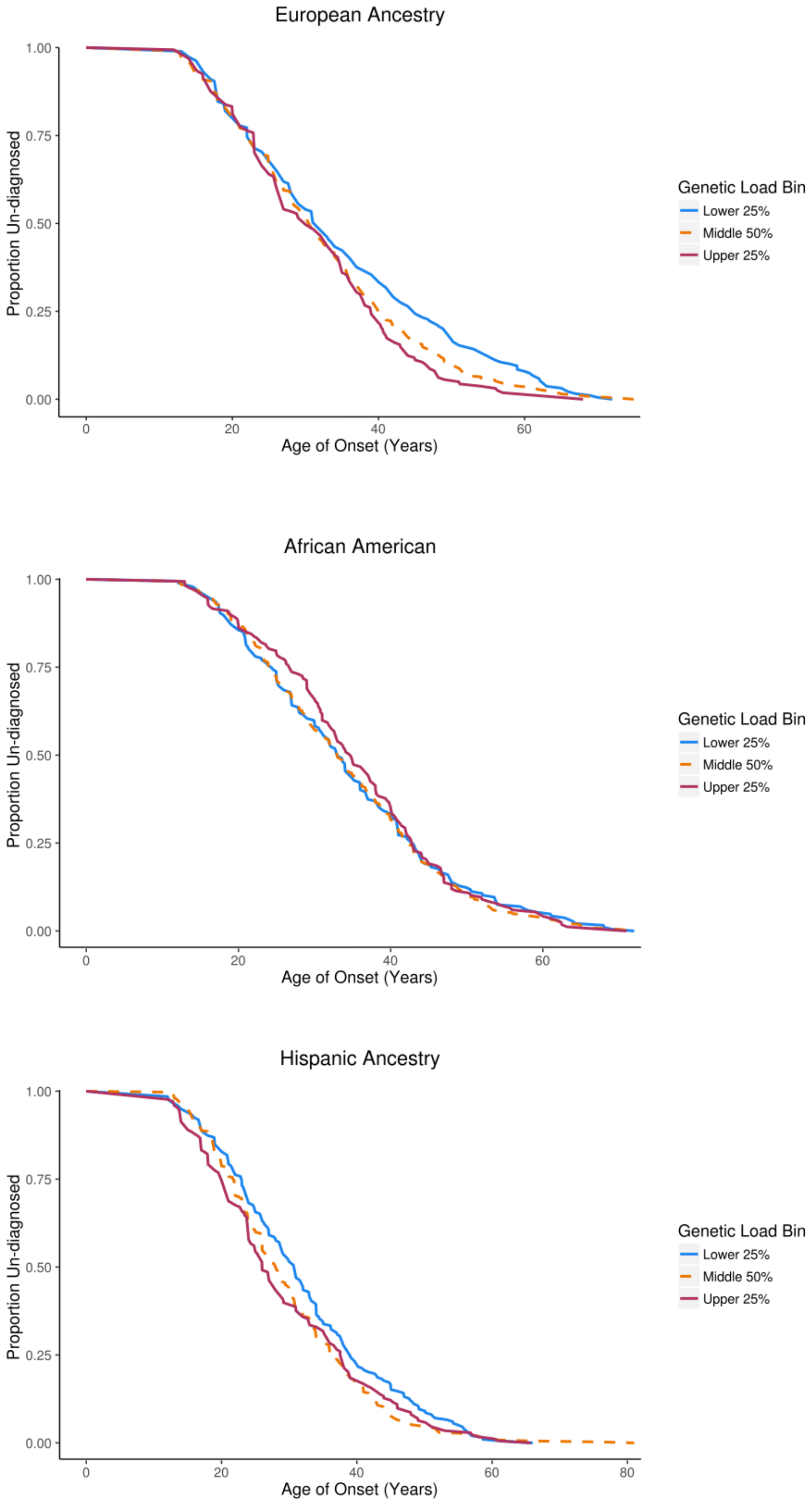

Supplement: Supplementary Information [file ncomms16021-s1.pdf]
